# Challenges in recurrent head and neck squamous cell cancer treatment: systematic review and *meta*-analysis comparing efficacy and toxicity between post-operative and definitive IMRT-based reirradiation

**DOI:** 10.1016/j.ctro.2025.101061

**Published:** 2025-10-25

**Authors:** Lukas Grajewski, Alicia Greiner, Georg Wurschi, Orlando Guntinas-Lichius, Alexander Rühle, Klaus Pietschmann, Maximilian Römer

**Affiliations:** a Department of Radiotherapy and Radiation Oncology, Jena University Hospital, Jena, Germany; b Comprehensive Cancer Center Central Germany, Partner Site Jena, Jena, Germany; c Department of Otorhinolaryngology, Jena University Hospital, Jena, Germany; d Department of Radiation Oncology, University Medical Center Leipzig, Leipzig, Germany; e Comprehensive Cancer Center Central Germany, Partner Site Leipzig, Leipzig, Germany

**Keywords:** IMRT, Reirradiation, Head and neck cancer, Post-operative Radiotherapy, Meta-analysis

## Abstract

**Background and purpose:**

Treatment of patients with recurrent head and neck squamous cell cancer in a previously irradiated field remains challenging. We performed a *meta*-analysis, comparing the efficacy and safety of definitive and post-operative intensity-modulated radiotherapy (IMRT) based reirradiation.

**Material and methods:**

MEDLINE, Cochrane Library, Web of Science, SCOPUS and PsycINFO were systematically searched. The Newcastle-Ottawa Scale was used to assess the risk of bias. A *meta*-analysis was performed using the random-effects model.

**Results:**

10 eligible studies with a median follow up of 24.9 months (9.6–78.1) and a total of 958 patients were identified, two of which are prospective trials. 455 were patients treated with post-operative/ adjuvant IMRT (aIMRT), while 503 patients received definitive IMRT (dIMRT). Median age was 62 years (48–63), 78 % were males and the rate of concomitant systemic therapy varied between 0 and 100 %.

Post-operative IMRT showed significantly higher 1-year overall survival of 68 %, compared to 55 % for dIMRT, with a risk reduction (RR) of 0.84 (95 % CI: 0.76–0.93). aIMRT achieved superior 1-year locoregional control (65 %) in comparison to dIMRT (58 %) with an RR of 0.89 (95 % CI: 0.798 to 0.997). All other endpoints did not reach statistical significance. The certainty of our findings was low, due to limitations in the included studies. Radiotoxicity was insufficiently reported and does not allow any conclusions.

**Conclusion:**

Post-operative IMRT achieves superior survival and tumor control and should be the preferred option for patients eligible for surgery. Careful patient evaluation and selection are fundamental to maximizing therapeutic efficacy while minimizing treatment-related toxicities.

## Highlights

• A *meta*-analysis comparing post-operative and definitive IMRT for HNSCC reirradiation.
• The results of 10 studies with 958 patients are included.
• 1-year overall survival is superior after post-operative IMRT.
• 1-year locoregional control is likewise superior after post-operative IMRT.

## Graphical abstract

**Figure:**
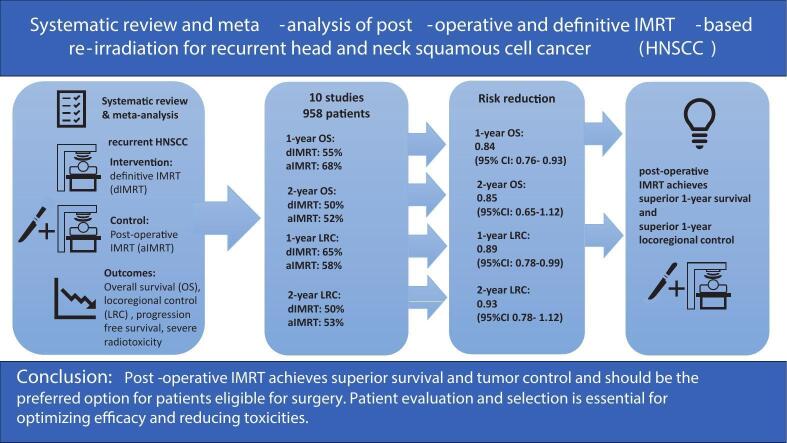

## 1 Introduction

Head and neck cancer is among the 10 most common cancers worldwide, according to the latest GLOBOCAN estimates [1]. Within this heterogeneous group, squamous cell carcinoma is the most important entity, accounting for approximately 90 % of diagnoses [2]. In recent decades, advances in diagnosis and treatment have led to a gradual increase in survival rates [3,4]. However, recent registry studies have shown that between 20 and 50 % of head and neck cancer patients will develop recurrent or secondary primary cancers [[5], [6], [7]]. Most of these recurrences are (loco)regional [8] and have been described to be more resistant to radiotherapy and chemotherapy [9].

Finding the optimal treatment for these patients remains a serious challenge for clinicians [10]. In general, the options for retreatment are radiotherapy (RT), surgery, chemotherapy, immunotherapy, or a combination of these options in a multimodal approach [11]. Complete surgical resection remains the recommended first-line treatment in both European [12] and US [13] guidelines. However, salvage surgery itself is associated with a relevant incidence of complications, especially in the often elderly recurrent head and neck squamous cell cancer (rHNSCC) population [14]. Positive resection margins and other risk factors, such as extracapsular spread and perineural invasion, also regularly indicate the need for additional adjuvant radiotherapy (aRT) [11]. However, not all patients are eligible for resection or refuse to undergo surgery [15]. For this population, a remaining curative treatment option is definitive radiotherapy (dRT), potentially in combination with systemic therapy [16,17]. When a high dose is delivered in a previously irradiated field, the potential benefits of better tumor control and survival must be weighed against the risk of serious adverse events [18,19], potentially resulting in complications with profound impact on the patient's quality of life [20] or even lethal outcomes [21,22].

Over the last few decades, huge developments in RT technology have led to the adoption of more precise conformal techniques, the most common of which is IMRT. With this technique, a sufficient dose can be delivered to the cancer site, while sparing organs at risk, such as the carotid artery [23,24], the parotid gland [[25], [26], [27]], the spinal cord [28,29]. The oncological and safety advantages of IMRT over three-dimensional reirradiation [[30], [31], [32]] have been described in numerous publications. As a result, IMRT has become the preferred approach to reirradiation (reRT) for rHNSCC [11,12]. A large *meta*-analysis published in 2020 demonstrated that IMRT-based reirradiation can provide effective tumor control and favourable survival outcomes [33]. Some studies found that surgery prior to reRT had a significant positive impact on prognosis [34,35] while others did not find this effect [[36], [37], [38]]. These results raise the question if surgery followed by adjuvant IMRT (aIMRT) might be superior to definitive IMRT-based reirradiation (dIMRT) for patients with previously irradiated recurrent and secondary primary head and neck squamous cell cancer? To address this question, a systematic review and *meta*-analysis comparing aIMRT with dIMRT in the context of reirradiation was performed.

## 2 Methods and materials

### 2.1 Protocol and search string

This systematic review and *meta*-analysis was conducted according to the 2020 Preferred Reporting Items for Systematic Reviews and Meta-analysis (PRISMA) guidelines [39]. A prospective analysis protocol was developed using the PRISMA-P template [40] and this review was prospectively registered on PROSPERO (CRD42025534269). The final protocol is available in the Supplements (B.1). MEDLINE, Cochrane Library, Web of Science, SCOPUS and PsycINFO were systematically searched. A professional librarian was involved in the construction of the search string. It consists of two general terms: a): ((Recurrent AND head and neck squamous cell cancer) AND radiotherapy) and b): (head and neck squamous cell carcinoma AND (reirradiation OR (repeat AND radiotherapy))). For each sub-concept, MeSH terms and all identified synonyms were used and the search strings were individually evaluated by two authors (LG and AG) according to the 2015 Peer Review of Electronic Search Strategies (PRESS) guidelines [41]. The completed search strings for each database are provided in the Supplementary Material (B.3a – B.3e). Search was restricted to articles in German or English language, published after 1 January 2005, as only recent studies using IMRT techniques that were mostly comparable to current standards should be included. EndNote 20 [42] was used as the citation manager of choice..

Two independent reviewers (LG, AG) performed de-duplication following the method described by Bramer et al. [43,44] as well as title and abstract screening in duplicate. Further attempts to identify additional articles were made by reference checking of all retrieved full text articles and searching on Clinicaltrial.gov [45], WHO International Clinical Trials Registry Platform (ICTRP) portal [46] and the Restoring invisible and abandoned trials (RIAT) Support Center [47].

Finally, possible overlap between cohorts was assessed. If there was an overlap between cohorts, only the larger cohort was included. In addition, study, selection, data extraction and Risk of bias (RoB) assessment were performed by two authors individually (AG and LG). Any discrepancies were resolved by discussion and re-evaluation of the literature. If consensus could not be reached, a third reviewer was consulted (MR).

### 2.2 Selection criteria

As rHNSCC is a heterogeneous group with variable prognosis between anatomic sites [48], the inclusion criteria were constructed to ensure maximum homogeneity of eligible studies while still identifying enough studies for quantitative synthesis. As nasopharyngeal cancer differs substantially in pathogenesis [49], treatment options [50,51] and outcome from other rHNSCC [52], studies with more than 20 % nasopharyngeal cancer patients, were excluded. The complete inclusion criteria can be seen in Supplementary Table A.2.

aIMRT was defined as postoperative IMRT-based radiotherapy, regardless of the surgical procedure, such as complete resection or salvage debulking. For dIMRT, only tumor biopsy was allowed. Primarily, only prospective trials were included because of their potentially lower risk of bias [53,54]. If fewer than three such articles could be identified, retrospective studies were also eligible.

### 2.3 Data extraction

Data extraction was performed using a standardised extraction template in Microsoft Excel [55]. The data extraction template included general study information, patient characteristics, and details pertaining to both the initial and re-treatment modalities. If this information was only available for the whole cohort and not for each arm separately, the values for the whole cohort were extracted. Trials were required to report at least the number of patients at baseline and the survival rate for at least one of the co-primary endpoints (1- or 2-year OS) to be included. If there was any uncertainty or if a missing value couldn't be found in the article and its supplements, the authors were contacted. When required, values were estimated from descriptive graphs. The method used to extract these graphs is described in the Supplementary Material. For loco-regional control, values stated as locoregional-progression-free-survival and the inverted values of loco-regional failure, were included.

### 2.4 Risk of bias assessment

The Newcastle-Ottawa-Scale (NOS) [56] Risk of bias (RoB) was independently assessed by LG and AG for each outcome domain using the Newcastle–Ottawa Scale (NOS). The NOS scores were subsequently converted to AHRQ standards based on predefined thresholds. Detailed information on the assessment procedure is provided in the Supplements (B.4).

If regression models were used to control for time to recurrence or reirradiation dose, only continuous regression was accepted as a sufficient approach. “Poor quality” studies were excluded from quantitative synthesis. Additionally, considerations for any conflict of interest, location- and language bias were assessed according to recommendations by the Cochrane handbook [57].

### 2.5 Statistical analysis

For planning and conducting the quantitative synthesis, a professional statistician was involved and the analysis was planned a priori. R version 4.4.2 “Pile of Leaves” was used for all analyses [58] with RStudio version 2025.05.0 as an integrated development environment [59]. A *meta*-analysis was only done, if at least three studies could be identified for each outcome. For analysis, “meta”- [60] and “metafor”-package [61] were primarily used. The function “metabin” was chosen and pooling was performed using the inverse variance weighting [62]. The relative risk for the desired effect (i.e. survival, control) was measured and the overall effect measure was risk ratio (RR) [63]. Outcomes (i.e. 1-year or 2-year OS) were dichotomized accordingly. If no direct data were available, values were extracted from Kaplan-Meier curves by two authors (LG and AG), individually. Then the average of both values was used. Individual patient data (IPD) was only available for one study (Rühle et al. [64]).

If retrospective studies were included, the random effects model was applied, only. Variance was estimated using the Paule-Mandel method [65] and heterogeneity among studies was assessed using *I*^2^ [66]. A contrast enhanced funnel plot [67] was constructed for each endpoint as well. More details can be found in the protocol (see Supplement Material B.1). If at least 10 studies were included, a *meta*-regression was performed using the “metareg” function. The influence of chemotherapy (below 50 % or above), reirradiation dose and postoperative risk-factors was assessed. The significance level was not adjusted because only a small number of pre-planned analyses were intended [68] and every endpoint would be assessed, individually. In this scenario, alpha adjustments were not needed to prevent spurious Type I errors [69]. So, a p-value <0.05 was considered significant for all analyses. The overall quality of evidence was assessed according to the GRADE approach using GRADEpro GDT [70] by AG and LG, individually. Any differences between the ratings were resolved by discussion, using recommendations by Prasad et al. [71] and the GRADE handbook [72].

## 3 Results

### 3.1 Characterisation of the included studies

**Fig. 1:**
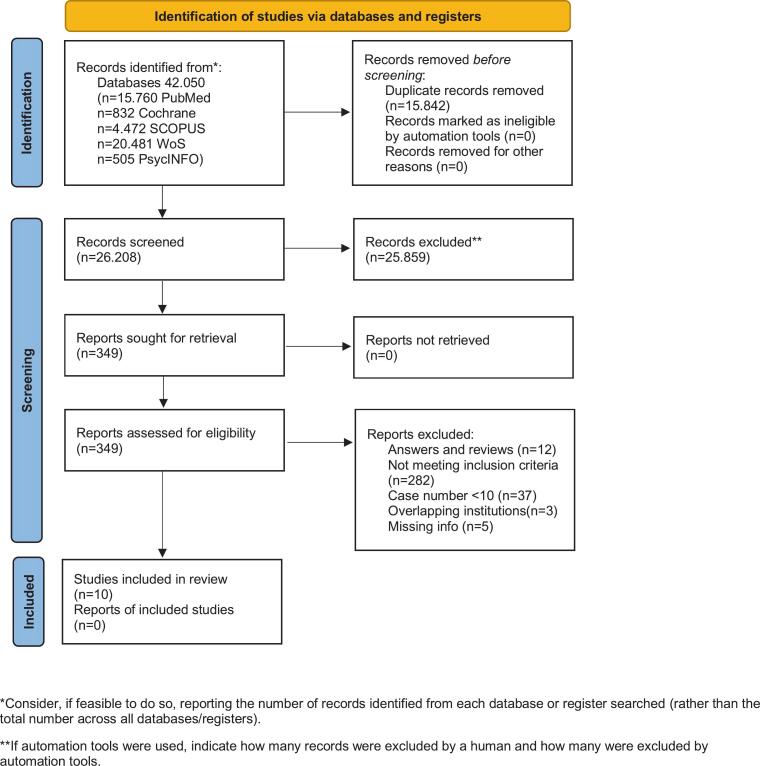
PRISMA flowchart visualizing the study selection process.

The initial systematic search, conducted on the 22nd of February 2025, identified 42.050 articles before de-duplication. The selection process is summarised in a PRISMA flowchart shown in Fig. 1 [39]. Comprehensive information on the screening of titles and abstracts is available in Supplementary Material B.5. A full list of all 349 retrieved studies and their assessment is provided in Supplementary Table A.1. Several studies were identified that compared post-operative and definitive reRT; however, they did not include a sufficient number of cases treated with IMRT [73,74] and were subsequently excluded from this review as it only focuses on state-of-the-art IMRT-based reRT.

As expected, some overlap was found in the cohorts. Research by Lee et al. [30] and Riaz et al. [75] reported on cohorts from the Memorial Sloan-Kettering Cancer Center, New York and compared LRC between aIMRT and dIMRT. As this institution was also included in a multi-institutional analysis by the MIRI collaborative, an overlap in cohorts between these three studies was suspected [76]. The multi-institutional work was chosen as it includes substantially more patients (412 compared to 105 [30] or 257 [75], respectively). If conference papers or abstracts for later published research were identified, the full-text article was included only, as it was peer-reviewed. Of the aforementioned study by Ward et al. [76], an abstract was identified, that was subsequently excluded, as well [77].

Dr. Alexander Rühle provided separate outcome data for patients without distant metastases. This subgroup comprises 44 of the originally published 58 patients, representing 71 % of the initial cohort [64]. These newly obtained values were be used for the main synthesis, as they result in substantially greater homogeneity among the eligible studies.

Finally, 10 eligible studies were identified, comprising the subgroup from Rühle et al. The included studies reported on a total of 958 patients, of whom 503 and 455 received dIMRT and aIMRT, respectively (see Table 2 for trial characteristics and Supplementary Tables A.3 and A.4 for further details). Only two prospective phase II trials [78,79] were eligible, so retrospective research was included as well. One trial was based on a prospective database [80], while the remaining seven were retrospective chart reviews [37,64,76,[81], [82], [83], [84]]. Two studies did not report the median follow-up period [64,84], the median follow-up time of all remaining trials was 15.3 months (range 9.6 [83] to 78.1 [83]). The median time between RT administrations ranged from 15 [84] to 48 [82] months, with one study not reporting this value [78]. 78 % of the included patients were male and the mean age was 62 years. The majority of patients presented with advanced disease at the time of reRT and ECOG performance status of 0 to 1. Most patients in the aIMRT group presented with surgical risk factors such as R1 or close margin status, perineural invasion, or extracapsular spread.

**Table 1:** Summary of findings.

Outcomes	Anticipated effects*		Relative effect(95 % CI)	No. of participants (No. of studies)	Certainty of the evidence(GRADE)	Comments
	adjuvant IMRT	definitive IMRT				
1-year overall survival	68 %	57 % (52 to 63)	RR 0.84 (0.76 to 0.93)	972(10 non-randomised studies)	⊕◯◯◯ Very low^a,b,c^	Definitive IMRT may result in lower 1-year overall survival than adjuvant IMRT, but the evidence is very uncertain.
2-year overall survival	47 %	40 % (30 to 52)	RR 0.85 (0.65 to 1.12)	972(10 non-randomised studies)	⊕◯◯◯ Very low^a,b,c,d^	There appears to be little to no difference in 2-year overall survival between definitive and adjuvant IMRT treatment, but the evidence is very uncertain.
1-year locoregional control	65 %	58 % (52 to 65)	RR 0.89 (0.798 to 0.997)	711(5 non-randomised studies)	⊕◯◯◯ Very low^a,b^	Definitive IMRT may result in lower 1-year locoregional control than adjuvant IMRT, but the evidence is very uncertain
2-year locoregional control	53 %	49 % (41 to 59)	RR 0.93 (0.78 to 1.12)	711(5 non-randomised studies)	⊕◯◯◯ Very low^a,f,g^	There appears to be little to no difference in 2-year locoregional control between definitive and adjuvant IMRT treatment, but the evidence is very uncertain.
1-year progression free survival	50 %	53 % (43 to 66)	RR 1.05 (0.85 to 1.31)	234(4 non-randomised studies)	⊕◯◯◯ Very low^a,e,f^	There appears to be little to no difference in 1-year progression free survival between definitive and adjuvant IMRT treatment, but the evidence is very uncertain.
2-year progression free survival	33 %	43 % (31 to 60)	RR 1.29 (0.92 to 1.81)	234(4 non-randomised studies)	⊕◯◯◯ Very low^a,f^	There appears to be little to no difference in 2-year progression free survival between definitive and adjuvant IMRT treatment, but the evidence is very uncertain.
Severe acute and late radiotoxicity	No conclusive findings can be reported on serious radiotoxicity between definitive and adjuvant IMRT-based re-irradiation			150(2 non-randomised studies)	⊕◯◯◯ Very low^a,h,i^	The evidence is very uncertain about the effect of definitive IMRT compared to adjuvant IMRT on severe acute and late radiotoxicity.
*The risk in the intervention (definitive IMRT) group (and its 95 % confidence interval) is based on the assumed risk in the comparison group and the relative effect of the intervention (and its 95 % CI). CI: confidence interval; RR: risk ratio						
GRADE Working Group grades of evidence High certainty: we are very confident that the true effect lies close to that of the estimate of the effect. Moderate certainty: we are moderately confident in the effect estimate: the true effect is likely to be close to the estimate of the effect, but there is a possibility that it is substantially different. Low certainty: our confidence in the effect estimate is limited: the true effect may be substantially different from the estimate of the effect. Very low certainty: we have very little confidence in the effect estimate: the true effect is likely to be substantially different from the estimate of effect.						

a. no randomisation was used. Important confounders were not controlled for.

b. wide 95% Confidence interval that cannot be attributed to heterogeneity among studies alone.

c. visual inspection of funnel plot shows asymmetry.

d. visually inconsistency and statistical analyses also showing heterogeneity.

e. visual inconsistency. Variation in chemotherapy regimen used. Too few studies identified.

f. very wide 95% Confidence interval. Only few studies with too few results identified.

g. visual inconsistency.

h. contrary results in narrative reviewi. Imprecise methods and report.

**Table 2:** Study characteristics. NR= Not reported, SCC= Squamous cell carcinoma, SIB= Simultaneous integrated boost, RT = Radiotherapy, ReRT = Reirradiation, IMRT = Intensity-Modulated Radiotherapy, 3DCRT = 3D Conformal Radiotherapy, 5-FU = Fluro-uracil, dIMRT = definitive IMRT-based therapy, aIMRT = adjuvant (post-operative) IMRT-based therapy, Pat.no = Patient number.

Study details				Patient number		median follow-up, months(range)	SCC %	median age, years (range)	Previous RT dose, Gy (range)	Interval between RT courses, months (range)	Reirradiation			
Author, year	Location	Study design	Inclusion period	dIMRT	aIMRT						Method used for re-irradiation	median ReRT dose, Gy (range)	median number of fractions (range)	Concurrent therapy % Agent (pat. no)
Awan, 2018	Multi Institution, US^a^	P Phase II	2009–2013	12	33	16.6(NR)	100	62 (36–85)	70 (63–75.6)	30 (6–220)	100 % IMRT	61.8 (60–70.3), 26.7 % SIB	30	100 % cetuximab and cisplatin
Biagioli, 2007	University of Miami Sylvester Comprehensive Cancer Center, US	R	2001–2006	24	17	14	86	63(19–82)	62.1 (NR)	25 (6–240)	100 % IMRT	60 (38–60.57)	30 (30–33)alternating weeks	100 % 63 % carboplatin, 37 % cisplatin +/- 5-fluorouracol
Chen, 2022	College of Medicine, Chang Gung University, Taoyuan, Taiwan	R	1999–2010	45	38	NR	100	48 (32–79)	64 (59.4–72)	15 (NR)	71 % IMRT (59), 29 % 3DCRT (24)	60 (28–80)	NR	88 % cisplatin or methotrexate (73), 4 %taxol-based (2)
Curtis, 2016	Mayo Clinic Scottsdale, US	R	2003–2011	39	42	78.1 (56–96.8)^d^	100	aIMRT 61 (34–83)dIMRT 65 (36–83)	dIMRT 66 (26.4–79.2) aIMRT 66 (50–72)	aIMRT (28.2 (20–48.6) CI,dIMRT 48.6 (25.1–78.2)	95 % IMRT (77), 5 % 3DCRT (4)	dIMRT: 69.6 (48–76.8) aIMRT: 60 (12–70)	NR	74.1 % cisplatin or cetuximab
Rühle, 2020	University of Freiburg Medical Center, Germany	R	2010–2019	31	17	NR	79.2	63 (27–96)	68 (30.8–72)	17 (4–176)	100 % IMRT, 31 % SIB	58.4 (3.7–66)	NR	58 % cetuximab (17), 17 % cisplatin (8), 6 % others (3)
Saba, 2024	Multi Institution, US^b^	P Phase II	2018–2021	13	38	24.5 (NR)	100	62 (56–67)	NR	NR	100 % IMRT	NR (60–66)	NR (30–33)	100 % nivolumab
Scolari, 2023	University Hospital Ruppin-Brandenburg, Germany	R x	2004–2021	31	30	9.8 (4.4–19-4)^e^	100	59.8(53.1–66.7)	NR	23.6 (8.4–76.6)	100 %IMRT	60 (54.6–60)^e^	50 (twice daily 1.2 Gy)	62 % platinum- based + 5-FU (38), 5 % Cetuximab (3)
Sulman, 2009	M.D. Anderson Cancer Center Houston, US	R	1999–2004	54	20	25 (0–81)	77	62 (20–84)	60 (16–75)	46 (3–445)	100 %IMRT	60 (15–70)	30 (NR)	42 % Platinum-based (31)
Velez, 2017	University of California, David Geffen Medical University, US	R	1998–2015	45	31	9.6 (NR)	79	57.5 (26.6–84.9)	68.4 (18.2–136)	25.3 (2–322)	93 % IMRT (71), 7 % SBRT (5)	60 (18–70)	30 (NR)	61.8 % cisplatin or cetuximab (47)
Ward, 2018	Multi-Institution, US^c^	R	1998–2015	217	195	10.4 (0–129.7)	100	62 (21–92)	66 (40–80)	29 (2–408)	100 % IMRT	60 (39.6–79.2)	33 (12–66) 20 % hyper-fractionated	75 % (309) 47 % platinum single (194), 30 % platinum-based combi (124), 30 % cetuximab (124)

a. Case Western Reserve University, University of Texas Southwestern; Rush University Medical Center; Medical College of Wisconsin

b. Winship Cancer Institute Emory University, Atlanta; Cleveland Clinic Foundation; Medical Collage of Wisconsin

c. Memorial Sloan-Kettering Cancer Center, Moffitt Cancer Center, the Josephine Ford Cancer Institute at Henry Ford Health System Detroit, University of Louisville, University Hospitals Case Medical Center Cleveland, Winship Cancer Institute at Emory University, Taussig Cancer Institute.

d. 95 % Confidence Interval used.

e. inter-quartal-range.

While the stage of recurrence was not consistently reported, the majority of patients presented with advanced disease at the time of reRT. A variety of systemic therapy regimens were encountered, the most common being platinum-based chemotherapy regimens using cisplatin or anti-EGFR-therapy [85] with cetuximab. Only Saba et al. administered an immunotherapy with nivolumab [78] (see Table 2). In most cases, conventionally fractionated radiotherapy was administered, although some studies employed hyperfractionated regimens [80].

Conflict of interest (COI) assessment can be found in Supplementary Table A.5. Most of the included trials did not have a serious risk of COI with one missing any statements on it [81]. The phase II trial by Saba et al. [78], was deemed to have a significant concern for COI as the pharmaceutical company providing the applied concomitant agent was involved during the trial design, reviewed the article and provided grant funding for the lead author.

The main results of the included trials are shown in Table 3 with the secondary endpoints provided in Supplementary Table A.6. Values were extracted directly or from graphical presentations (see Supplementary Table A.7).

**Table 3:** Study resultsOS = Overall survival.

Author, year	1-year OS		2-year OS	
	dIMRT	aIMRT	dIMRT	aIMRT
Awan, 2018	33 %	71 %	33 %	50 %
Biagioli, 2007	58 %	75 %	37 %	55 %
Chen, 2022	40 %	53 %	17 %	42 %
Curtis, 2016	69 %	76 %	48 %	53 %
Rühle, 2020	57 %	74 %	48 %	60 %
Saba, 2024	83 %	85 %	71 %	41 %
Scolari, 2023	40 %	42 %	25 %	13 %
Sulman, 2009	79 %	74 %	74 %	58 %
Velez, 2017	50 %	70 %	37 %	70 %
Ward, 2018	52 %	67 %	36 %	45 %

### 3.2 1-year and 2-year overall survival

Overall survival was reported by 10 studies including 958 patients. When assessing the RoB for OS, all articles were rated “good quality” with 8 to 9 stars. However, concerns about confounding remained because time between radiotherapy courses was not controlled for in some trials. An overview of the final NOS rating can be found in Supplementary Table A.8.

**Fig. 2:**
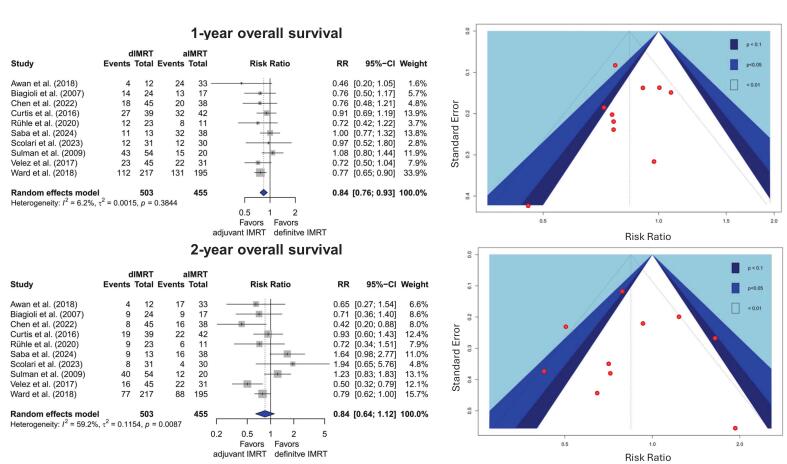
Meta-analysis and Funnel plot for 1-and 2-year overall survival. A random effects model was used.

The quantitative synthesis for 1-year OS showed a statistically significant survival advantage for aIMRT over dIMRT (see Fig. 1). In the surgically treated group, a total of 314 out of 455 patients were alive (68 %) compared to 282 out of 503 (55 %) in the definitively treated cohort. This corresponds to a relative risk (RR) for 1-year OS of 0.84 (95 % CI 0.76 to 0.93). The certainty of this result was still considered “very low” according to the GRADE criteria [72]. One factor for this rating was a significant asymmetry identified by examining the contrast-enhanced funnel plot (Fig. 2). Thus, publication bias must be strongly suspected. The full GRADE evidence table is shown in Supplementary Table A.9.

The aforementioned superiority of aIMRT at 12 months was not statistically significant for 2-year OS. After two years, 47 % of the aIMRT patients were reported to be alive compared to 40 % in the dIMRT group. This results in an RR of 0.85 (95 %CI: 0.65–1.12). Again, the certainty was rated as “very low”. Much higher heterogeneity with an I^2^ = 58.9 % (p = 0.009) was observed for 2-year OS, which different methods for outcome measurement could explain, as some studies have a median follow-up far below 24 months [37,80,83]. The proportion of patients receiving concomitant systemic therapy did not significantly predict 1-year or 2-year survival outcomes (p = 0.4178 and p = 0.6773) in *meta*-regression. As only six studies reported on margin status and the median reRT dose showed limited variability, these factors could not be analysed.

### 3.3 1-year and 2-year loco-regional control

**Fig. 3:**
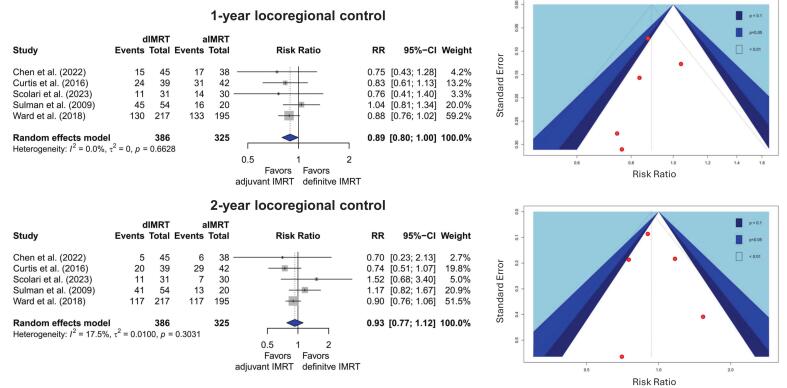
Meta-analysis and Funnel plot for 1-and 2-year locoregional control. A random effects model was used.

Five trials reporting on 711 patients were identified that reported on locoregional control [76,81,82] (Fig. 3). In the RoB assessment, control of the recurrence interval and exclusion of palliative intent reRT were assessed in the comparability domain (see Supplementary Table A.10). For 1-year LRC, a very small but statistically significant benefit was observed in favour aIMRT with an RR of 0.89 (95 % CI: 0.798 to 0.997, p = 0.045, random effects model) as seen in Fig. 2. The locoregional control rates after 12 months for aIMRT were 65 % and 58 % for dIMRT. Furthermore, with the exception of Sulman et al., this effect was consistent and comparable in magnitude across the four remaining included studies.

For 2-year local control, the results were more indecisive and inconsistent, with a non-significant pooled RR of 0.93 (95 % CI: 0.78–1.12). Adjuvant IMRT and dIMRT had a pooled 2-year locoregional control of 53 % and 50 %, respectively. For both, 1-year and 2-year LRC, the funnel plot appeared more symmetric upon inspection. As expected, the LRC data showed a higher number of losses to follow-up compared to the OS data [76,82]. It should be noted that these results were also assessed to be of low certainty (see Table 1 and Supplementary Table A.9).

### 3.4 1-year and 2-year progression-free survival

This endpoint was only reported in four trials [64,78,80,81] (see Supplementary Table A.6). All four studies were rated “good quality” although some did not sufficiently control for concurrent systemic therapy applied (Supplementary Table A.11). A total of 220 patients were included − 121 and 99 in the dIMRT and aIMRT cohorts, respectively (Table 2).

**Fig. 4:**
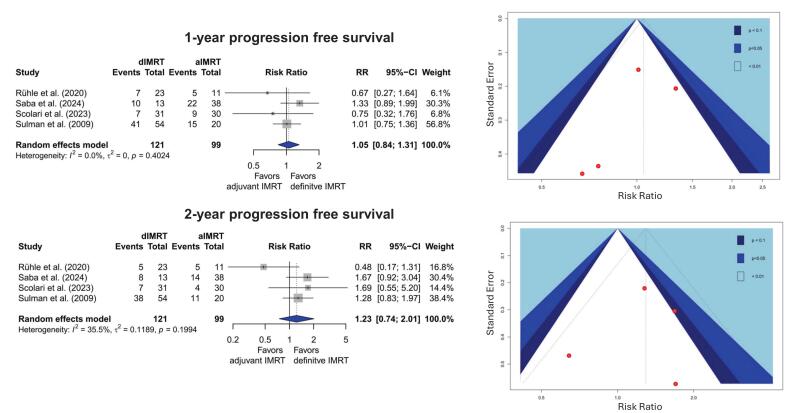
Meta-analysis and Funnel plot for 1-and 2-year progression free survival. A random effects model was used.

The results for 1-year progression-free survival (PFS), were very inconsistent within the identified studies, resulting in a non-significant RR in the aIMRT cohort of 1.05 (95 %CI: 0.85–1.31). Here, slightly higher values were achieved by dIMRT with 53 % compared to 51 % in the postoperative setting (Fig. 4).

For 2-year PFS, the results were somewhat more consistent, with three studies suggesting improved outcomes in the dIMRT group, whereas the trial by Rühle et al. indicated the contrary [64]. However, no statistically significant difference in 2-year PFS between the two groups was observed, with a relative risk (RR) of 1.29 (95 % CI: 0.92–1.81). 2-year PFS for aIMRT was 33 % compared to 47 % for dIMRT (Fig. 4). In addition to the inconsistency of the results described above, the sample size was very small, the overall confidence interval was very wide, and a funnel plot asymmetry towards better dIMRT results was noted, which was particularly pronounced for 2-year PFS. Furthermore, an imbalance for censored events was noted. Furthermore, Saba et al. reported a higher proportion of censored events in the dIMRT group (70 %) compared to the aIMRT cohort (40 %) [78], while Rühle et al. showed a more balanced distribution, with 30 % (dIMRT) and 27 %(aIMRT), respectively [64].The certainty of these results was rated as “very low” (see Table 1 and Supplementary Table A.9). In addition, concerns about a conflict of interest in one study [78] existed (see 3.1 or Supplementary Table A.5).

### 3.5 Severe radiotoxicity

Rtox was separately reported for aIMRT and dIMRT by only two studies [81,83]. The methodology used in these articles was considered to be noticeably different as seen in Supplementary Table A.12. Therefore, the extracted results can only be described qualitatively.

In total, 15 cases of severe Rtox (defined in this article as Rtox resulting in hospitalisation and/or corrective surgery or death), were reported by Sulman et al. [81], 3 of which occurred in the surgically treated group, representing 15 % of this cohort. Of these three, one patient died within 30 days of an unspecified cause, which was classified as a treatment-related death. The remaining 12 reported cases of severe Rtox occurred in patients treated with dIMRT, constituting 22 % of this group. No deaths were reported in this group.

Velez et al. reported the event rate of ≥Grade 3 late RTox for aIMRT and dIMRT separately. Late Rtox was defined as symptoms occurring more than 90 days after completion of treatment, using the RTOG/EORTC Radiation Morbidity Scoring Scheme. 29 % of the aIMRT cohort were described as having late Rtox ≥grade 3 compared to 36 % of the definitively treated patients. No treatment-related deaths were reported in this publication. On univariate and multivariate Cox regression, the influence of salvage surgery on ≥grade 3 late Rtox was insignificant [83].

However, all 10 eligible articles report Rtox for both groups combined. The identified cumulative radiotoxicity will be briefly presented.

Again, considerable variation in methodology was identified, with some studies reporting only toxicities likely to be attributable to reRT and the remainder reporting all adverse events: haematological and otherwise. For better comparability, all reported toxicities likely to be attributed to reRT, such as dysphagia, dermatitis, mucositis, xerostomia, osteoradionecrosis, carotid blowout, fistulas, pain, dysgeusia, hoarseness, nausea, trismus, oesophageal stricture, presence of feeding tube and tracheostomy (not attributed to surgery), were summarised (see Supplementary Table A.13). Note that the adverse events reported by Ward et al. [76] were excluded from this table as it was not possible to clearly extract the desired values.

In most reports, variants of the CTCAE-scale (V3.0 [79,84], V4.0 [76], V4.03 [64,78]) were used. For toxicity, the number of described severe Rtox ≥grade 3 events was extracted and described in relation to all treated patients of that cohort. Thus, values above 100 % were possible, as every patient could have experienced numerous toxicities. The reported rate of serious acute Rtox ≥grade 3 varies considerably between 10 % [64] and 100 % [80] while the rate of late radiotoxicity ≥grade 3 ranged from 11 % [79] to 42 % [84].

Under platinum-based therapy, Biagioli et al. reported an additional 14 grade 3 and 4 toxicities, representing 23 % of the whole cohort of 41 patients, while 25 patients experienced no serious adverse event [37]. Curtis et al. described an additional 17 acute events ≥grade 3 out of 83 patients analysed [82] in contrast to 60 additional serious events in 48 patients with a cisplatin and cetuximab combination presented by Awan et al. [79]. Under nivolumab treatment for 51 patients, 22 additional acute complications ≥grade 3 were observed [78]. Among these, typical immune-related adverse events [86] were reported with one case of colitis, Myasthenia gravis and myositis, each (See Supplementary Table A.14).

## 4 Discussion

Reirradiation for rHNSCC remains challenging, with concerns about treatment-related toxicity and an overall unfavourable prognosis. The present study is the first systematic approach to compare definitive and adjuvant reirradiation using modern, highly conformal techniques for rHNSCC.

A *meta*-analysis by Lee et al. of IMRT-based reRT reported 2-year OS and local control rates of 46 % and 53 %, respectively [33]. This *meta*-analysis included studies regardless of resection status. The range of aIMRT within the included studies varies between 0 % and 80 % with a median of 41.5 %. Comparable results were found for our aIMRT cohort (47 % 2-year OS, 53 % 2-year LRC) although somewhat lower values were noted for the definitively treated patients (40 % 2-year OS, 50 % 2-year LRC). The *meta*-analysis by Lee et al. reported significantly higher 2-year locoregional control (LRC) in a subgroup of patients with more than 42 % undergoing surgery for recurrence, compared to trials with lower aIMRT utilization [33]. This effect on two-year LRC could not be replicated in our analysis, but a significant advantage for aIMRT on one-year LRC was identified. Notably, our study included a different set of trials than the aforementioned *meta*-analysis [33], with only four trials represented in both papers [37,76,79,83].

The MIRI collaborative presented a model to predict survival after re-IMRT, the MIRI-RPA. It stratifies rHNSCC patients into three classes according to three factors: time to recurrence, pre-existing organ dysfunction and surgical resection. Class I consists of resected patients with late recurrence, regardless of margin status, having a significantly better prognosis than the unresected patients in class II [76]. A significantly better 1-year OS was achieved in the aIMRT cohort, which may support the validity of this risk stratification. However, it should be noted that the MIRI cohort, which was the basis for this risk model, was also included in our analysis. Interestingly, for early recurrences with baseline organ dysfunction, class III, surgery does not seem to substantially affect outcome [76]. However, beyond that observation, it was not possible to comprehensively apply the MIRI-RPA to our data as statements about organ dysfunction were often times missing and IPD was only available for one study, making it impossible to stratify our population into MIRI-RPA classes. However, further testing and validation of this prognostic model based on contemporary real-world data remains an interesting topic for future research.

Our work has some serious limitations that need to be considered by clinical practitioners and decision-makers.

A major limitation of this study is potential publication bias, evidenced by funnel plot asymmetry suggesting an overrepresentation of significant results in the OS analysis. For all the other outcomes of interest, there were too few studies to be sure about this source of bias. In addition, some values had to be extracted from graphical presentations, a method that could introduce rounding errors. IPD were only available for a single trial, which represents a substantial limitation and led our analyses to rely predominantly on aggregate data. The search was limited to results published in English or German, which may introduce a bias towards the treatment and outcomes of Western patients, as otherwise relevant studies published in other languages may have been overlooked.

As a systematic review, this work inherits the strengths and weaknesses of the included studies. The majority of the studies included were retrospective and have a significant risk of bias inherent in their design. In particular, the lack of randomisation must be taken into account. Most patients were likely to be referred to the dIMRT group because of contraindications to surgery. What excludes patients from salvage surgery is not uniformly defined [14] but could include cancer localisation adjacent to vital structures or severe medical comorbidity [87] which could be a significant confounder for worse OS and LRC in the dIMRT group. Cancer localisation varied between the trials and most of the included studies only reported the characteristics of the whole cohorts [76,78,81,83,88]. There might be some undetected confounding in the unequal distribution of cancer sites between aIMRT and dIMRT, with indications that prognosis is also influenced by tumor localisation [48,89]. HPV positive HNSCC is associated with a better prognosis [90,91], even in the recurrent setting [92,93] and the rates of HPV positive cancer in the compared groups were not sufficiently stated.

For PFS, neither approach seems to be superior to the other. However, the evidence base is very thin and prone to confounding, as only one of the included trials used the same concomitant systemic therapy for both groups [78]. In the other three trials it is possible that more resected patients did not receive systemic therapy compared to the dIMRT group. These values could also be unequally influenced by confounding due to missing data and loss to follow-up. Surgery may also have an independent effect on some types of concomitant therapy. Saba et al. hypothesised that neck dissection negatively influences the efficacy of nivolumab [78]. This is supported by the fact that non-metastatic lymph nodes still have an important immune function, especially when immunotherapy is admninisterd [94].

A major limitation of our work is our inability to adequately compare Rtox and treatment-related death between the two groups. The trial by Suman et al. and the one by Velez et al. showed roughly comparable rates of severe Rtox between aIMRT and dIMRT with an event rate of approximately 20–30 % in both groups [81,83]. Still, these studies used different methodologies and were too small to draw definitive conclusions. Publications by Lee et al. [95,96] and Phuong et al. [97,98] did not find a significant difference in long-term Rtox rates between aIMRT and dIMRT, while Margalit et al. [99] found lower toxicity rates in the surgically treated population. It is likely that surgery alone is not a sufficient predictor of severe late Rtox as seen in another work by Ward et al. Here the incidence of late Rtox was plotted against the MIRI RPA classes with insufficient results. This study presented a more complex risk nomogram. However, there was a clearer relationship between MIRI RPA classes and freedom from late toxicity, death or cancer progression. [100] Further research is needed to increase the certainty of these results.

The prevalence of surgical risk factors in the aIMRT-cohort, such as R1 margin status varied between the included studies (see Supplementary Table A.2). These factors could introduce serious confounding, as measurable residual disease is associated with a worse prognosis [101,102]. The benefit of adjuvant radiotherapy compared with surgical resection alone is beyond the scope of this paper but patients with residual disease after surgical resection or other high-risk features may especially benefit from post-operative reRT. A randomized trial by Janot et al. involving 130 patients, most of whom had histological risk factors, demonstrated that post-operative reRT significantly improved disease-free survival compared to salvage surgery alone. However, overall survival was not significantly affected, and a trend toward increased ≥grade 3 toxicities [103] was noted in aIMRT patients while a comparable trial investigating post-operative SBRT failed to recruit a sufficient number of patients [104]. Furthermore, Ramprasad et al. reported that multimodal treatment, including post-operative reRT, was associated with diminished patient-reported quality of life (QoL) compared to surgery alone [105].

Any treatment decision should take into account the patient's own wishes and preferences [106]. The impact of head and neck cancer on patients' lives is complex [107] and none of the identified trials assess QoL between aIMRT and dIMRT separately. Future studies are needed to address these uncertainties.

Finally, the optimal method of reRT also needs to be discussed. SBRT [108], proton therapy [109] and brachytherapy [110] all show promising results for reirradiation of rHNSCC as they allow for highly conformal dose delivery which may result in fewer complications [111,112]. SBRT in particular is of increasing interest as it is often more readily available than proton therapy or brachytherapy [113,114]. Ongoing rHNSCC trials using SBRT for reRT include NCT06211335 and the RTOG KEYSTROKE trial (NCT03546582).

## 5 Conclusion

Given the limited therapeutic options available for each individual patient, both dIMRT and aIMRT represent effective and feasible treatment strategies. Nevertheless, when clinically appropriate, surgical resection followed by post-operative reirradiation should be favoured, as it may offer superior outcomes. Clinicians should, however, remain mindful of the potential risks associated with each approach, as well as the low certainty of our results.

## CRediT authorship contribution statement

**Lukas Grajewski:** Conceptualization, Data curation, Formal analysis, Investigation, Methodology, Project administration, Software, Supervision, Validation, Visualization, Writing – original draft. **Alicia Greiner:** Investigation, Writing – review & editing. **Georg Wurschi:** Validation, Writing – review & editing. **Orlando Guntinas-Lichius:** Conceptualization, Writing – review & editing. **Alexander Rühle:** Writing – review & editing. **Klaus Pietschmann:** Conceptualization, Project administration, Resources, Supervision, Validation, Writing – review & editing. **Maximilian Römer:** Conceptualization, Investigation, Methodology, Resources, Supervision, Validation, Writing – review & editing.

## Informed consent

Informed consent and approval of an ethics committee were waived due to the nature of this analysis.

## Funding

No funding or other forms of financial support were received.

## Declaration of competing interest

The authors declare that they have no known competing financial interests or personal relationships that could have appeared to influence the work reported in this paper.

## Data Availability

The data supporting the findings of this review can be found in peer-reviewed articles, which are available on their journals’ websites. Authors‘ correspondence is available on reasonable request.
