# Supplementary material for: Challenges in recurrent head and neck squamous cell cancer treatment: systematic review and meta-analysis comparing efficacy and toxicity between post-operative and definitive IMRT-based reirradiation
Source: Clin Transl Radiat Oncol. 2025 Oct 25;56:101061. doi: 10.1016/j.ctro.2025.101061 (PMC12630038; doi:10.1016/j.ctro.2025.101061)
Supplement: Supplementary Data 1 [file mmc1.docx]

**PRISMA-P (Preferred Reporting Items for Systematic review and Meta-Analysis Protocols) 2015 checklist: recommended items to address in a systematic review protocol**

| Section and topic | Item No | Checklist item |
| --- | --- | --- |
| ADMINISTRATIVE INFORMATION | | |
| Title: |  | **Comparing results of definitive and postoperative Radiotherapy for recurrent head and neck cancer in the modern era. Analysis of toxicity and survival in a systematic review and meta-analysis of IMRT and VMAT treatment** |
| Identification | 1a | Systematic Review and Meta-analysis |
| Update | 1b | No |
| Registration | 2 | registered via PROSPERO; ID 534285 |
| Authors: |  |  |
| Contact | 3a | Universitätsklinikum Jena, Germany. Klinik für Strahlentherapie und Radioonkologie. Lukas Mark Grajewski, Friedrich-Schiller-Universität, Jena (Germany) mail: [lukas.grajewski@uni-jena.de](mailto:lukas.grajewski@uni-jena.de)  Alicia Sophie Greiner, Friedrich-Schiller-Universität Jena (Germany) mail: alicia.sophie.greiner@uni-jena.de  Dr. Maximilian Römer, Uniklinikum Jena (Germany) mail: [maximilian.roemer@med.uni-jena.de](mailto:maximilian.roemer@med.uni-jena.de) Dr. Klaus Pietschmann, Universitätsklinikum Jena (Germany) mail: klaus.pietschmann@med.uni-jena.de  Postal address:  Universitätsklinikum Jena, Germany. Klinik für Strahlentherapie und Radioonkologie,  Kastanienstraße1 - D-07747 Jena |
| Contributions | 3b | **Lukas Grajewski** (LG): Main author. Develops protocol, responsible for registering, designs search strategy (with librarian), selecting literature, extracting data, evaluating Risk of Bias (With AG), conducting Synthesis and Meta-analysis (with statistician), evaluating GRADE (with AG) and writing the final article.  **Alicia Greiner** (AG): selecting literature, extracting data, evaluating Risk of Bias and final GRADE-evidence (With LG) **Dr. Maximilian Römer** (MR): third author, to be consulted if disagreement between LG and AG.  **Dr. Klaus Pietschmann** (KP) contributes expertise and is consulted for peer reviewing and finalizing protocol and search strategy. Might be consulted when disagreement with Risk of Bias assessment occurs.  Guarantor of the review: Lukas Grajewski |
| Amendments | 4 | Amendment to this protocol will be clearly identified as such and all changes will be listed and explained. The updated protocol will be uploaded on PROSPERO and provided to the review. |
| Support: |  |  |
| Sources | 5a | No financial support was given to development of this review. |
| Sponsor | 5b | There is no review funder and/or sponsor |
| Role of sponsor or funder | 5c | - |
| INTRODUCTION | | |
| Rationale | 6 | Squamous cell carcinoma of the head and neck (SCCHN) is the sixth most common neoplasm worldwide and its incidence is expected to increase. Tobacco and alcohol abuse are consistently identified as major risk factors, followed by pollutants and viral infections (HPV and EBV).  Between 15 and 50% of these patients experience a recurrence or second primary HNC. This is a major contributor to head and neck cancer mortality. Current treatment regimens for these recurrent cancers consist primarily of salvage surgery, radio(chemo)therapy and treatment with checkpoint inhibitors. Highly conformal radiotherapy using Intensity Modulated Radiotherapy (IMRT) and Volumetric Arc Therapy (VMAT) offers new opportunities for previously irradiated patients. This systematic o evaluates the outcome of adjuvant versus definitive IMRT-VMAT based radiotherapy in this setting.  The relevance of this topic was positively assessed with the FINER- criteria (Farrugia et al. 2010) for suitability for meta-analysis.  Feasible: Yes, enough suitable trials are likely to exist.  Interesting: Yes, the authors have sufficient personal interest to conduct this review.  Novel: Yes, new evidence emerged and such a review has not yet been done.  Ethical: Yes, uses existing published and unpublished data.  Relevant: Yes, the prognosis of SCCHN recurrences still rather poor and additional research is reasonable.  This work may help to identify the patient groups that benefit most from re-irradiation, and thus lead to current clinical advice. |
| Objectives | 7 | The following PICO-scheme was developed:  P: Patients with recurrent or second primary SCCHN (at least 70% of the study population must have histologically proven squamous cell primary). Older than 18 years, received radiotherapy during initial treatment. Not diagnosed with nasopharyngeal cancer (trials with more than 20% nasopharyngeal cancer will be excluded) At least 50% of the irradiated cancers should be located in the following regions: Oral cavity, Oropharynx, Hypopharynx, Larynx, Neck.  I: IMRT/VMAT (>70% of the population must be treated for recurrence with this system) with at least fifteen fractions of at least 1 Gy each. Delivered in a previously irradiated field (>90% of the study population must have received radiotherapy in overlapping fields) with or without concurrent chemotherapy. Patients must have undergone surgery for their recurrence (at least 90% post-operative re-irradiation), no additional Brachytherapy or SBRT  C: IMRT/VMAT (>70% of the population must be treated for recurrence with this system) with at least fifteen fractions of at least 1 Gy each. Delivered in a previously irradiated field (>90% of the study population must have received radiotherapy in overlapping fields) with or without concurrent chemotherapy. Patients must not undergo surgery for their recurrence (max 10% post-operative re-irradiation in this population) no additional Brachytherapy or SBRT  O: Critical (primary) outcome: 1-year and 2-year overall survival (OS)  secondary outcomes: 1-year locoregional control, 2-year locoregional control 1-year progression-free survival, 2-year progression free survival, radiotherapy-related acute toxicities defined as ≥grade 3 (in grading system stated in each study), treatment related death. |
| METHODS | | |
| Eligibility criteria | 8 | We will primarily include prospective studies. If enough prospective studies cannot be identified (>5), retrospective studies, such as chart reviews, will also be included. These studies should follow patients from the start of an intervention to the achievement of relevant outcomes (survival, radiotoxicity, etc.).  The search will be limited to sources available in English and German and published or (in the case of unpublished literature) created after 1 January 2005.  Single case reports and case series with unsystematic clinical reporting will be excluded. Studies must contain at least 10 participants on each arm. |
| Information sources | 9 | A systematic search of MEDLINE, Web of Science, Cochrane Library, PsycINFO, Scopus, reference lists and journals will be conducted.  The search will be pre-run to identify missed synonyms to improve the final search strategy.  Unpublished and grey literature will be searched by reviewing conference abstracts, dissertations and other reports. RIAT will also be consulted for this purpose.  The final search strategies will be peer-reviewed by the AG according to the PRESS 2015 guidelines (McGowan et al. 2016). All search strategies will be documented and made available for transparency. A librarian will be involved in the refinement of the strategy. |
| Search strategy | 10 | The final search strategy for all websites used will be reported separately. It will be sufficiently detailed to allow reproduction. A professional librarian will be involved in the development of this strategy. |
| Study records: |  |  |
| Data management | 11a | A PRISMA flow-chart should be created(Page et al. 2021).  All found studies will be imported into the data management program **EndNote20(Team 2013).**  For data extraction **Microsoft Excel(2018)** should be used. For statistical analysis **R Studio(2024)** will be used. |
| Selection process | 11b | A test run of the eligibility criteria should be carried out on a sample of reports. Any inconveniences will be resolved and changes noted.  The selection of studies will be carried out by two different people in duplicate. (LG and AG). Duplicates in studies will be removed using a modified Bramer method(Bramer et al. 2016), titles and abstracts will be screened, and in case of uncertainty the full text will be obtained.  The results were discussed and disagreements were resolved with the involvement of a third person (MR). Authors may also be contacted if important data is unclear or missing.  If enough good-quality trials can be identified, only those should be included in meta-analysis  In the case of different articles involving the same cohort (i.e. same institution and overlapping time period), the larger cohort will be selected for inclusion. |
| Data collection process | 11c | The reviewers will be instructed on how to use the data form designed in Microsoft Excel and a pilot run will be carried out on some articles to resolve any uncertainties. Again, the results will be discussed and a third person (MR) will be consulted if there is still disagreement. Missing data will be dealt with by contacting the study authors. The correspondence is documented and made available. |
| Data items | 12 | A statistician is consulted when designing the data extraction sheet to ensure that all relevant data are identified for extraction. List of the extracted data:  Description of study methods/design:   1. Prospective vs. retrospective study, Setting: clinical, preclinical (phase I, II, III), unclear or retrospective 2. Region(s) and country(ies) from which study participants were recruited 3. Single/multi-centre (how many centres, where?) 4. Duration of the study, Follow-up period 5. Analysis per protocol or intention to treat 6. Methods used to prevent and control confounding; Methods used to prevent and deal with missing data (described in free text or "missing") 7. Important covariates and confounders controlled for 8. Source(s) of funding or other material support for the study 9. Financial relationships of authors and other potential conflicts of interest. 10. Other factors:     1. Was an Ethical approval obtained     2. Was a Protocol made before the trial began     3. Was a Sample-size caLRCulation done a priori? (only prospective trials)     4. Was a Power analysis performed? (only prospective trials)   Describe population   - 1. Age   2. Gender   3. Comorbidity (according to what measurement system)   4. reported cancer stage and grading (according to which version of which staging system)   5. MIRI-RPA(Ward et al. 2018), if used   6. smoking history: current/ formally/ none   7. aLRCohol consumption, betel nut chewing   8. HPV status (p16 or viral DNA PCR), PD-1L status   9. time of recurrent neoplasia <2 years; >2 year   10. GTV and PTV re-irradiated   11. cumulative radiation dose   12. previous therapy (surgery %, chemotherapy % and drugs used)   13. recurrent or second primary cancer (according to what definition)   14. primary site   15. site of recurrence   Treatment characteristics   1. Fractionation scheme(s) used and total re-irradiation dose separately for <60Gy and above 2. What linear-accelerator and radiation method was used? 3. Means of identifying target volume, process of planning the radiation 4. Was a simultaneous- integrated-boost applied? 5. Where statements for organ-at-risk sparing given? 6. Additional surgery (% and R1 status) 7. Induction chemotherapy (% and drugs used) 8. Integrity of interventions   Relevant co-interventions are:   1. Additional Chemotherapy (5-Flouro-uracil etc.) 2. Additional treatment with Checkpoint inhibitors 3. immunotherapy   All relevant missing data should be extracted or collected via contacting the authors |
| Outcomes and prioritization | 13 | **Critical primary outcome** will be 1-year and 2-year overall survival. They will be measured separately as relative risk for desired effect (OS).  Additional outcomes: 1-year locoregional control (LRC), 1-year progression free survival (PFS) as well as 2-year PFS, represent important parameters to evaluate treatment effectiveness. They will be measured separately as relative risk for desired effect (OS, LRC, PFS) and as a percentage.  Severe Radiotoxicity is defined as => Grade 3 (CTCAE V5) as well as treatment related death are important parameters for safety. They will be measured separately as the relative risk for toxicity. |
| Risk of bias in individual studies | 14 | Risk of bias will be assessed at the study level.  Risk of bias (RoB) for randomised trials will be assessed using the Cochrane RoB tool 2.  Non-randomised studies will be assessed using the Newcastle-Ottawa-scale. A brief explanation is provided for each rating.  RoB using the Cochrane RoB Tool 2 or Newcastel-Ottawa Scale will be assessed individually by two different people (LG and AG) and the tools will then be piloted on some articles. The final results will be compared and discussed. Disagreements will be resolved by consulting a third experienced person (MR or KP). The study authors could be contacted if there are still uncertainties. If consensus is still not reached, this will be noted in the review.  The following additional factors must be characterised and discussed by one author (LG) and peer reviewed and checked by another author (AG or MR):   - 1. Location bias   2. Language bias (especially as we only search the German and English literature)   3. publication bias could be assessed using a funnel plot. (see below).   4. Conflicts of interest should be assessed. |
| Data synthesis | 15a | A meta-analysis will only be done when at least 4 studies are identified that report the outcome of interest. If the non-randomized trials features are too different to combine, the pooled effect should be eliminated from the Forrest plot. |
|  | 15b | If non-randomised trials are included, high heterogeneity is expected, so the random-effects model variables is the default choice. Only studies that analyse the relevant outcome are included. Consistency will be evaluated with I^2^  I^2^ will be evaluated against the following thresholds proposed by Cochrane Handbook(Boutron et al.):  0% to 40%: might not be important;  40% to 60%: may represent moderate heterogeneity;  50% to 80%: may represent substantial heterogeneity;  80% to 100%: considerable heterogeneity  The meta-analysis will compare surgery and adjuvant radio-chemotherapy with definitive IMRT  The following outcomes will be compared:   1. 1 year OS 2. 2-year OS 3. 1-year PFS 4. 2-year PFS 5. 1-year LRC 6. 2-year LRC 7. Incidence of treatment related death 8. Incidence of severe acute Radiotoxicity 9. Incidence of severe late Radiotoxicity |
|  | 15c | Sub-group analysis via meta-regression will only be done when sufficient number of studies are included in comparison (>10) a statistician should be consulted.   1. Analyse if concurrent chemotherapy has an impact on 1-year overall survival in the intervention (surgery) group. 2. Analyse Studies with mean overall re-radiation dose (> 60Gy) versus lower for both groups (intervention and control). 1-and 2-year overall survival should be assessed individually. 3. Analyse 1-and 2-year overall in populations with patients with close/ positive margins to those threated without these risk factors   The significance level will not adjusted because only a small number of pre-planned analyses is intended and every endpoint will be assessed, individually(Armstrong 2014). In this scenario, alpha adjustments are not be needed to prevent spurious Type I errors(García-Pérez 2023). A p value of <0.05 will be considered significant. |
|  | 15d | In case a quantitative summary of any endpoint is not appropriate a qualitative summary will be given in accordance to (Campbell et al. 2020) |
| Meta-bias(es) | 16 | Many ways of Bias are already evaluated on an individual study level. (See above).  Publication bias is assessed using a contour-mapped funnel plot(Sterne und Egger 2001). If more than 10 trials can be included and the trials are of different sizes, a test for asymmetry (Eggers regression) is performed((Egger et al. 1997)  The funnel plot is then discussed.  It will be reported which study is missing from which synthesis due to missing / unreported data. This should include a description of which specific data are missing.  It should be discussed whether the missing data leads to bias within the synthesis. |
| Confidence in cumulative evidence | 17 | All relevant results are then presented in a table for ease of reading.  This includes the most important outcomes and the overall quality as assessed by GRADE for randomised or non-randomised trials. GRADEpro(2025) software will be used to produce good tables.  If possible: For dichotomous outcomes, a relative and absolute effect measure will be used: Odds ratio and odds reduction.  The GRADE assessment should be carried out by two individuals (LG and AG). The results will be discussed and if there is no consensus, a third person (MR) will be involved. |

*From: Shamseer L, Moher D, Clarke M, Ghersi D, Liberati A, Petticrew M, Shekelle P, Stewart L, PRISMA-P Group. Preferred reporting items for systematic review and meta-analysis protocols (PRISMA-P) 2015: elaboration and explanation. BMJ. 2015 Jan 2;349(jan02 1):g7647.*

This protocol completed on 29^th^ of January 2025. Edited on 20^th^ of April: Included references.

References (not complete)

20.04.2025. Restoring Invisible & Abandoned Trials Support Center <https://restoringtrials.org/>.

18.04.2025. GRADE handbook for grading quality of evidence and strength of recommendations. <https://gdt.gradepro.org/app/handbook/handbook.html>.

2018. Microsoft Excel Redmond, WA: Microsoft Corporation.

2024. R: A Language and Environment for Statistical Computing. R Foundation for Statistical Computing R Core Team

2025. GRADEpro GDT: GRADEpro Guideline Development Tool gradepro.org: McMaster University and Evidence Prime.

Armstrong RA. 2014. When to use the Bonferroni correction. Ophthalmic Physiol Opt, 34 (5):502-508.

Boutron I, Page M, Higgins J, Altman D, Lundh A, Hróbjartsson A 20.04.2025. Chapter 7: Considering bias and conflicts of interest among the included studies <https://training.cochrane.org/handbook/current/chapter-07>.

Bramer WM, Giustini D, de Jonge GB, Holland L, Bekhuis T. 2016. De-duplication of database search results for systematic reviews in EndNote. J Med Libr Assoc, 104 (3):240-243.

Campbell M, McKenzie JE, Sowden A, Katikireddi SV, Brennan SE, Ellis S, Hartmann-Boyce J, Ryan R, Shepperd S, Thomas J, Welch V, Thomson H. 2020. Synthesis without meta-analysis (SWiM) in systematic reviews: reporting guideline. Bmj, 368:l6890.

Egger M, Davey Smith G, Schneider M, Minder C. 1997. Bias in meta-analysis detected by a simple, graphical test. Bmj, 315 (7109):629-634.

Farrugia P, Petrisor BA, Farrokhyar F, Bhandari M. 2010. Practical tips for surgical research: Research questions, hypotheses and objectives. Can J Surg, 53 (4):278-281.

García-Pérez MA. 2023. Use and misuse of corrections for multiple testing. Methods in Psychology, 8:100120.

McGowan J, Sampson M, Salzwedel DM, Cogo E, Foerster V, Lefebvre C. 2016. PRESS Peer Review of Electronic Search Strategies: 2015 Guideline Statement. J Clin Epidemiol, 75:40-46.

Page MJ, McKenzie JE, Bossuyt PM, Boutron I, Hoffmann TC, Mulrow CD, Shamseer L, Tetzlaff JM, Akl EA, Brennan SE, Chou R, Glanville J, Grimshaw JM, Hróbjartsson A, Lalu MM, Li T, Loder EW, Mayo-Wilson E, McDonald S, McGuinness LA, Stewart LA, Thomas J, Tricco AC, Welch VA, Whiting P, Moher D. 2021. The PRISMA 2020 statement: an updated guideline for reporting systematic reviews. Bmj, 372:n71.

Sterne JA, Egger M. 2001. Funnel plots for detecting bias in meta-analysis: guidelines on choice of axis. J Clin Epidemiol, 54 (10):1046-1055.

Team TE. 2013. EndNote [64 bit]. Philadelphia, PA: Clarivate.

Ward MC, Riaz N, Caudell JJ, Dunlap NE, Isrow D, Zakem SJ, Dault J, Awan MJ, Vargo JA, Heron DE, Higgins KA, Beitler JJ, Marcrom S, Boggs DH, Hassanzadeh C, Reddy CA, Bonner JA, Yao M, Machtay M, Siddiqui F, Trotti AM, Lee NY, Koyfman SA. 2018. Refining Patient Selection for Reirradiation of Head and Neck Squamous Carcinoma in the IMRT Era: A Multi-institution Cohort Study by the MIRI Collaborative. Int J Radiat Oncol Biol Phys, 100 (3):586-594.
