# Supplementary material for: Challenges in recurrent head and neck squamous cell cancer treatment: systematic review and meta-analysis comparing efficacy and toxicity between post-operative and definitive IMRT-based reirradiation
Source: Clin Transl Radiat Oncol. 2025 Oct 25;56:101061. doi: 10.1016/j.ctro.2025.101061 (PMC12630038; doi:10.1016/j.ctro.2025.101061)
Supplement: Supplementary Data 2 [file mmc2.pdf]

Checklist according to PRESS Guidelines 2015

The following search strategy was assessed:

Peer reviewer:
Date:

Criteria	Question	Fulfilled?
**Translation of the research question**	Does the search strategy match the research question/PICO?<br>Are the search concepts clear?<br>Are there too many or too few PICO elements included?<br>Are the search concepts too narrow or too broad?<br>Does the search retrieve too many or too few records?<br>Are unconventional or complex strategies explained?	
**Boolean and proximity operators (these vary based on search service)**	Are Boolean or proximity operators used correctly?<br>Is the use of nesting with brackets appropriate and effective for the search?<br>If NOT is used, is this likely to result in any unintended exclusions?<br>Could precision be improved by using proximity operators (eg, adjacent, near, within) or phrase searching instead of AND?<br>Is the width of proximity operators suitable (eg, might adj5 pick up more variants than adj2)?	
**Subject headings (database specific)**	Are the subject headings relevant?<br>Are any relevant subject headings missing; for example, previous index terms?<br>Are any subject headings too broad or too narrow?<br>Are subject headings exploded where necessary and vice versa?<br>Are major headings ("starring" or restrict to focus) used?<br>If so, is there adequate justification?	

Modified by Lukas Grajewski after McGowen et al.: "PRESS Peer Review of Electronic Search Strategies: 2015 Guideline Statement",2016 DOI: 10.1016/j.jclinepi.2016.01.021

	Are subheadings missing?<br>Are subheadings attached to subject headings? (Floating subheadings may be preferred.)<br>Are floating subheadings relevant and used appropriately?<br>Are both subject headings and terms in free text (see the following) used for each concept?	
**Text word searching (free text)**	Does the search include all spelling variants in free text (eg, UK vs. US spelling)?<br>Does the search include all synonyms or antonyms (eg, opposites)?<br>Does the search capture relevant truncation (ie, is truncation at the correct place)?<br>Is the truncation too broad or too narrow?<br>Are acronyms or abbreviations used appropriately?<br>Do they capture irrelevant material?<br>Are the full terms also included?<br>Are the keywords specific enough?<br>Are too many or too few keywords used? Are stop words used?<br>Have the appropriate fields been searched; for example, is the choice of the text word fields (.tw.) or all fields (.af.) appropriate?<br>Are there any other fields to be included or excluded (database specific)?<br>Should any long strings be broken into several shorter search statements?	
**Spelling, syntax, and line numbers**	Are there any spelling errors?<br>Are there any errors in system syntax; for example, the use of a truncation symbol from a different search interface?<br>Are there incorrect line combinations or orphan lines (ie, lines that are not referred to in the final summation that could indicate an error in an AND or OR statement)?	
**Limits and filters**	Are all limits and filters used appropriately and are they relevant given the research question?<br>Are all limits and filters used appropriately and are they relevant for the database?<br>Are any potentially helpful limits or filters missing?<br>Are the limits or filters too broad or too narrow?<br>Can any limits or filters be added or taken away?<br>Are sources cited for the filters used?	

Notes:

Modified by Lukas Grajewski after McGowen et al.: “PRESS Peer Review of Electronic Search Strategies: 2015 Guideline Statement”,2016 DOI: 10.1016/j.jclinepi.2016.01.021
