# Supplementary material for: Challenges in recurrent head and neck squamous cell cancer treatment: systematic review and meta-analysis comparing efficacy and toxicity between post-operative and definitive IMRT-based reirradiation
Source: Clin Transl Radiat Oncol. 2025 Oct 25;56:101061. doi: 10.1016/j.ctro.2025.101061 (PMC12630038; doi:10.1016/j.ctro.2025.101061)
Supplement: Supplementary Data 3 [file mmc3.pdf]

Search strategy for Cochrane Libary.

1st Concept

((MeSH descriptor: [Head and Neck Neoplasms] explode all trees
OR Head And Neck Squamous Cell Carcinoma*:ti,ab,kw
OR HNSCC :ti,ab,kw
OR Squamous Cell Carcinoma of the Head and Neck :ti,ab,kw
OR Carcinoma, Squamous Cell of Head and Neck :ti,ab,kw
OR Squamous Cell Carcinoma of Larynx :ti,ab,kw
OR Laryngeal Squamous Cell Carcinoma*:ti,ab,kw
OR Hypopharyngeal Squamous Cell Carcinoma*:ti,ab,kw
OR Oral Squamous Cell Carcinoma* :ti,ab,kw
OR Squamous Cell Carcinoma of the Mouth :ti,ab,kw
OR Oropharyngeal Squamous Cell Carcinoma :ti,ab,kw
OR Oral Tongue Squamous Cell Carcinoma :ti,ab,kw
OR HNSC :ti,ab,kw
OR Squamous Cell Head and Neck Tumor* :ti,ab,kw
OR SCC of the Head and Neck :ti,ab,kw
OR Head and Neck Epithelial Cancer* :ti,ab,kw
OR Head and Neck Cancer of Squamous Origin :ti,ab,kw
OR Head, Neck Neoplasm*:ti,ab,kw
OR Neck Cancer*:ti,ab,kw
OR Upper Aerodigestive Tract Neoplasm*:ti,ab,kw
OR UADT Neoplasm*:ti,ab,kw
OR Head Neoplasm*:ti,ab,kw
OR Head and neck tumor [tw]
OR Craniofacial neoplasm*:ti,ab,kw
OR Craniofacial tumor* [tw]
OR Cervicofacial cancer*:ti,ab,kw
OR Orofacial neoplasm*:ti,ab,kw
OR Head and neck malignanc*:ti,ab,kw
OR Orofacial cancer*:ti,ab,kw
OR Craniofacial malignanc*:ti,ab,kw
OR Cervicofacial malignanc* :ti,ab,kw)

AND

(MeSH descriptor: [Re-Irradiation] explode all trees
OR Repeat irradiation*:ti,ab,kw
OR Reirradiation* :ti,ab,kw
OR Re-Irradiation* :ti,ab,kw
OR Second irradiation*:ti,ab,kw
OR Repeated radiation therap*:ti,ab,kw
OR Radiation re-treatment* :ti,ab,kw
OR Re-radiation* :ti,ab,kw
OR Repeated irradiation procedure* :ti,ab,kw
OR Salvage radiation therap* :ti,ab,kw)

2nd Concept

((MeSH descriptor: [Recurrence] explode all trees
OR recurrence* :ti,ab,kw
OR recurrent :ti,ab,kw
OR return of :ti,ab,kw
OR relapse* :ti,ab,kw
OR second* :ti,ab,kw)
AND
(MeSH descriptor: [Head and Neck Neoplasms] explode all trees
OR Head And Neck Squamous Cell Carcinoma*:ti,ab,kw
OR HNSCC :ti,ab,kw
OR Squamous Cell Carcinoma of the Head and Neck :ti,ab,kw
OR Carcinoma, Squamous Cell of Head and Neck :ti,ab,kw
OR Squamous Cell Carcinoma of Larynx :ti,ab,kw
OR Laryngeal Squamous Cell Carcinoma*:ti,ab,kw
OR Hypopharyngeal Squamous Cell Carcinoma*:ti,ab,kw
OR Oral Squamous Cell Carcinoma* :ti,ab,kw
OR Squamous Cell Carcinoma of the Mouth :ti,ab,kw
OR Oropharyngeal Squamous Cell Carcinoma' :ti,ab,kw
OR Oral Tongue Squamous Cell Carcinoma* :ti,ab,kw
OR HNSC :ti,ab,kw
OR Squamous Cell Head and Neck Tumor* :ti,ab,kw
OR SCC of the Head and Neck :ti,ab,kw
OR Head and Neck Epithelial Cancer* :ti,ab,kw
OR Head and Neck Cancer of Squamous Origin :ti,ab,kw
OR Head, Neck Neoplasm*:ti,ab,kw
OR Neck Cancer*:ti,ab,kw
OR Upper Aerodigestive Tract Neoplasm*:ti,ab,kw
OR UADT Neoplasm*:ti,ab,kw
OR Head Neoplasm*:ti,ab,kw
OR Head and neck tumor* :ti,ab,kw
OR Craniofacial neoplasm*:ti,ab,kw
OR Craniofacial tumor* :ti,ab,kw
OR Cervicofacial cancer*:ti,ab,kw
OR Orofacial neoplasm*:ti,ab,kw
OR Head and neck malignanc*:ti,ab,kw
OR Orofacial cancer*:ti,ab,kw
OR Craniofacial malignanc*:ti,ab,kw
OR Cervicofacial malignanc* :ti,ab,kw))
AND

(MeSH descriptor: [Radiotherapy] explode all trees
OR Radiotherap* :ti,ab,kw
OR Radiation Therap*:ti,ab,kw
OR Radiation Treatment*:ti,ab,kw
OR Targeted Radiotherap*:ti,ab,kw
OR Radiation :ti,ab,kw
OR Image-Guided Radiotherap* :ti,ab,kw
OR Image Guided Radiation Therap*:ti,ab,kw
OR IMRT :ti,ab,kw

OR Target Organ Alignment Radiotherapy :ti,ab,kw
OR Modulated radiation therap*:ti,ab,kw
OR Intensity Modulated radiation therap*:ti,ab,kw
OR IMXT :ti,ab,kw
OR Intensity-modulated beam therapy :ti,ab,kw
OR Conformal radiation therap*:ti,ab,kw
OR Intensity Modulated radiation treatment :ti,ab,kw
OR Precision radiation therap*:ti,ab,kw
OR Volumetric-Modulated Arc Therap*:ti,ab,kw
OR Intensity-Modulated Arc Therap*:ti,ab,kw
OR Helical Tomotherap*:ti,ab,kw
OR Gamma Knife Radiosurger*:ti,ab,kw
OR Stereotactic Radiation*:ti,ab,kw
OR Stereotactic Radiosurger*:ti,ab,kw
OR Linear Accelerator Radiosurger*:ti,ab,kw
OR LINAC Radiosurger*:ti,ab,kw
OR Stereotactic Body Radiotherap*:ti,ab,kw
OR CyberKnife Radiosurger*:ti,ab,kw
OR Stereotactic Radiation Therap*:ti,ab,kw
OR SBRT :ti,ab,kw
OR Radiological therap* :ti,ab,kw
OR SABR :ti,ab,kw
OR Stereotactic ablative radiotherap*:ti,ab,kw
OR SRS :ti,ab,kw
OR Stereotactic external beam radiotherap*:ti,ab,kw
OR Cyber Knife :ti,ab,kw
OR Focused radiation therap*:ti,ab,kw
OR Radiosurgical ablation :ti,ab,kw
OR Radiation oncology treatment* :ti,ab,kw
OR External beam therap* :ti,ab,kw)

Final search Strategy: 1st Concept OR 2nd Concept

Filters applied: Language German, English and publication: 2005-now
