# Supplementary material for: Challenges in recurrent head and neck squamous cell cancer treatment: systematic review and meta-analysis comparing efficacy and toxicity between post-operative and definitive IMRT-based reirradiation
Source: Clin Transl Radiat Oncol. 2025 Oct 25;56:101061. doi: 10.1016/j.ctro.2025.101061 (PMC12630038; doi:10.1016/j.ctro.2025.101061)
Supplement: Supplementary Data 4 [file mmc4.pdf]

Via EBSCOhost: eBook Collection, APA PycINFO and PsycArticles as well as Psycology and Behavioral siences collection will be searched.

1st Concept

Head Neck Cancer
OR Squamous Cell Carcinoma of Head and Neck
OR Head and Neck Neoplasm
OR Head And Neck Squamous Cell Carcinoma
OR HNSCC
OR Squamous Cell Carcinoma of the Head and Neck
OR Carcinoma, Squamous Cell of Head and Neck
OR Squamous Cell Carcinoma of Larynx
OR Laryngeal Squamous Cell Carcinoma*
OR Hypopharyngeal Squamous Cell Carcinoma*
OR Oral Squamous Cell Carcinoma*
OR Squamous Cell Carcinoma of the Mouth
OR Oropharyngeal Squamous Cell Carcinoma*
OR Oral Tongue Squamous Cell Carcinoma*
OR HNSC
OR Squamous Cell Head and Neck Tumor*
OR SCC of the Head and Neck
OR Head and Neck Epithelial Cancer*
OR Head and Neck Cancer of Squamous Origin
OR Head, Neck Neoplasm
OR Neck Cancer*
OR Upper Aerodigestive Tract Neoplasm
OR UADT Neoplasm
OR Head Neoplasm
OR Head and neck tumor*
OR Craniofacial neoplasm
OR Craniofacial tumor*
OR Cervicofacial cancer*
OR Orofacial neoplasm
OR Head and neck malignanc*
OR Orofacial cancer*
OR Craniofacial malignanc*
OR Cervicofacial malignanc* (AB)

AND

Repeat irradiation*
OR Reirradiation*
OR Re-Irradiation*
OR Second irradiation
OR Repeated radiation therap*
OR Radiation re-treatment*
OR Re-radiation*
OR Repeated irradiation procedur*
OR Salvage radiation therap*
OR Repeat Concurrent Chemoradiotherap*
OR Repeat Concomitant Chemoradiotherap*
OR Repeat Chemoradiotherap*, Concomitant

OR Repeat Chemoradiotherap*, Concurrent
OR Repeat Synchronous Chemoradiotherap*
OR Repeat Chemoradiotherap*, Synchronous
OR Repeat Radiochemotherap*, synchronous
OR Salvage Radiochemotherap*
OR Salvage Concurrent Chemoradiotherap*
OR Salvage Concomitant Chemoradiotherap*
OR "Salvage Chemoradiotherap*, Concomitant
OR Salvage Chemoradiotherap*, Concurrent
OR Salvage Synchronous Chemoradiotherap*
OR Salvage Chemoradiotherap*, Synchronous
OR Salvage Radiochemotherap*, synchronous (AB)

2nd Concept

(recurrence
OR recurrent
OR return of
OR relapse*
OR second* (AB)
AND
SSCHNC)

AND

Radiation Therapy
OR Radiotherapy, Image-Guided
OR Radiosurgery
OR Radiotherapy, Intensity-Modulated
OR Radiotherap*
OR Radiation Therap*
OR Radiation Treatment
OR Targeted Radiotherap*
OR Radiation
OR Image-Guided Radiotherap*
OR Image Guided Radiation Therap*
OR IMRT
OR Target Organ Alignment Radiotherap*
OR Modulated radiation therap*
OR Intensity Modulated radiation therap*
OR IMXT
OR Intensity-modulated beam therap*
OR Conformal radiation therap*
OR Intensity Modulated radiation treatment*
OR Precision radiation therap*
OR Volumetric-Modulated Arc Therap*
OR Intensity-Modulated Arc Therap*
OR Helical Tomotherap*

OR Gamma Knife Radiosurger*
OR Stereotactic Radiation
OR Stereotactic Radiosurger*
OR Linear Accelerator Radiosurger*
OR LINAC Radiosurger*
OR Stereotactic Body Radiotherap*
OR CyberKnife Radiosurger*
OR Stereotactic Radiation Therap*
OR SBRT
OR Radiological therap*
OR SABR
OR Stereotactic ablative radiotherapy*
OR SRS*
OR Stereotactic external beam radiotherapy*
OR Cyber Knife
OR Focused radiation therap*
OR Radiosurgical ablation
OR Radiation oncology treatment*
OR External beam therap*
OR Radiochemotherap*
OR Concurrent Chemoradiotherap*
OR Concomitant Chemoradiotherap*
OR Chemoradiotherap*, Concomitant
OR Chemoradiotherap*, Concurrent
OR Synchronous Chemoradiotherap
OR Chemoradiotherap*, Synchronous
OR Radiochemotherap* , synchronous (AB)

Final search Strategy: 1st Concept OR 2nd Concept

Filters applied: Language German, English and publication: 2005-now
