# Supplementary material for: Challenges in recurrent head and neck squamous cell cancer treatment: systematic review and meta-analysis comparing efficacy and toxicity between post-operative and definitive IMRT-based reirradiation
Source: Clin Transl Radiat Oncol. 2025 Oct 25;56:101061. doi: 10.1016/j.ctro.2025.101061 (PMC12630038; doi:10.1016/j.ctro.2025.101061)
Supplement: Supplementary Data 5 [file mmc5.pdf]

Search strategy for PubMed.

1st Concept

("Squamous Cell Carcinoma of Head and Neck"[Mesh]
OR "Head and Neck Neoplasms"[Mesh]
OR “Head And Neck Squamous Cell Carcinoma*”[tw]
OR HNSCC [tw]
OR “Squamous Cell Carcinoma of the Head and Neck”[tw]
OR “Carcinoma, Squamous Cell of Head and Neck”[tw]
OR ”Squamous Cell Carcinoma of Larynx”[tw]
OR ”Laryngeal Squamous Cell Carcinoma*”[tw]
OR ” Hypopharyngeal Squamous Cell Carcinoma*”[tw]
OR “Oral Squamous Cell Carcinoma*“[tw]
OR “Squamous Cell Carcinoma of the Mouth“[tw]
OR “Oropharyngeal Squamous Cell Carcinoma*“[tw]
OR “Oral Tongue Squamous Cell Carcinoma*”[tw]
OR HNSC [tw]
OR “Squamous Cell Head and Neck Tumor”[tiab:~0]
OR “SCC of the Head and Neck”[tw]
OR “Head and Neck Epithelial Cancer”[tiab:~0]
OR “Head and Neck Cancer of Squamous Origin”[tiab:~0]
OR “Head, Neck Neoplasm*”[tw]
OR “Neck Cancer*”[tw]
OR “Upper Aerodigestive Tract Neoplasm*”[tw]
OR “UADT Neoplasm*”[tw]
OR “Head Neoplasm*”[tw]
OR “Head and neck tumor*”[tw]
OR “Craniofacial neoplasm*”[tw]
OR “Craniofacial tumor*”[tw]
OR “Cervicofacial cancer*”[tw]
OR “Orofacial neoplasm*”[tw]
OR “Head and neck malignanc*”[tw]
OR “Orofacial cancer*”[tw]
OR “Craniofacial malignanc*”[tw]
OR “Cervicofacial malignancy”[tiab:~0])

AND

("Re-Irradiation"[Mesh]
OR “Repeat irradiation*”[tw]
OR Reirradiation*[tw]
OR Re-Irradiation*[tw]
OR “Second irradiation*”[tw]
OR “Repeated radiation therap*”[tw]
OR “Radiation re-treatment”[tiab:~0]
OR Re-radiation*[tw]
OR “Repeated irradiation procedure”[tiab:~0]
OR “Salvage radiation therap*”[tw]
OR “Repeat Concurrent Chemoradiotherapy”[tiab:~0]
OR “Repeat Concomitant Chemoradiotherapy”[tiab:~0]
OR ”Repeat Chemoradiotherapy, Concomitant” [tiab:~0]
OR “Repeat Chemoradiotherapy, Concurrent” [tiab:~0]
OR “Repeat Synchronous Chemoradiotherapy”[tiab:~0]
OR “Repeat Chemoradiotherapy, Synchronous” [tiab:~0]

OR “Repeat Radiochemotherapy, synchronous” [tiab:~0]
OR “Salvage Radiochemotherap*”[tw]
OR “Salvage Concurrent Chemoradiotherapy”[tw]
OR “Salvage Concomitant Chemoradiotherapy”[tiab:~0]
OR ”Salvage Chemoradiotherapy, Concomitant” [tiab:~0]
OR “Salvage Chemoradiotherapy, Concurrent” [tiab:~0]
OR “Salvage Synchronous Chemoradiotherapy”[tiab:~0]
OR “Salvage Chemoradiotherapy, Synchronous” [tiab:~0]
OR “Salvage Radiochemotherapy, synchronous” [tiab:~0])

2nd Concept

((recurrence[tw]
OR recurrent[tw]
OR “return of” [tw]
OR relapse*[tw]
OR second*[tw])
AND
("Squamous Cell Carcinoma of Head and Neck"[Mesh]
OR “Head and Neck Neoplasms”[Mesh]
OR “Head And Neck Squamous Cell Carcinoma*”[tw]
OR HNSCC[tw]
OR “Squamous Cell Carcinoma of the Head and Neck”[tw]
OR “Carcinoma, Squamous Cell of Head and Neck” [tw]
OR ”Squamous Cell Carcinoma of Larynx”[tw]
OR ”Laryngeal Squamous Cell Carcinoma*”[tw]
OR ” Hypopharyngeal Squamous Cell Carcinoma*”[tw]
OR “Oral Squamous Cell Carcinoma*“[tw]
OR “Squamous Cell Carcinoma of the Mouth“[tw]
OR “Oropharyngeal Squamous Cell Carcinoma*“[tw]
OR “Oral Tongue Squamous Cell Carcinoma*”[tw]
OR HNSC[tw]
OR “Squamous Cell Head and Neck Tumor”[tiab:~0]
OR “SCC of the Head and Neck”[tw]
OR “Head and Neck Epithelial Cancer”[tiab:~0]
OR “Head and Neck Cancer of Squamous Origin”[tiab:~0]
OR “Head, Neck Neoplasm*”[tw]
OR “Neck Cancer*”[tw]
OR “Upper Aerodigestive Tract Neoplasm*”[tw]
OR “UADT Neoplasm*”[tw]
OR “Head Neoplasm*”[tw]
OR “Head and neck tumor*”[tw]
OR “Craniofacial neoplasm*”[tw]
OR “Craniofacial tumor*”[tw]
OR “Cervicofacial cancer*”[tw]
OR “Orofacial neoplasm*”[tw]
OR “Head and neck malignanc*”[tw]
OR “Orofacial cancer*”[tw]
OR “Craniofacial malignanc*”[tw]
OR “Cervicofacial malignancy”[tiab:~0]))
AND

("Radiotherapy, Image-Guided"[Mesh]
OR “Radiosurgery"[Mesh]
OR "Radiotherapy, Intensity-Modulated"[Mesh]
OR Radiotherap*[tw]
OR “Radiation Therap*”[tw]
OR “Radiation Treatment*”[tw]
OR “Targeted Radiotherap*”[tw]
OR “Radiation”[tw]
OR “Image-Guided Radiotherap*”[tw]
OR “Image Guided Radiation Therap*”[tw]
OR IMRT[tw]
OR “Target Organ Alignment Radiotherapy”[tiab:~0]
OR “Modulated radiation therap*”[tw]
OR “Intensity Modulated radiation therap*”[tw]
OR IMXT[tw]
OR “Intensity-modulated beam therapy”[tiab:~0]
OR “Conformal radiation therap*”[tw]
OR “Intensity Modulated radiation treatment*”[tw]
OR “Precision radiation therap*”[tw]
OR “Volumetric-Modulated Arc Therap*”[tw]
OR “Intensity-Modulated Arc Therap*”[tw]
OR “Helical Tomotherap*”[tw]
OR “Gamma Knife Radiosurger*”[tw]
OR “Stereotactic Radiation*”[tw]
OR “Stereotactic Radiosurger*”[tw]
OR “Linear Accelerator Radiosurger*”[tw]
OR “LINAC Radiosurger*”[tw]
OR “Stereotactic Body Radiotherap*”[tw]
OR “CyberKnife Radiosurger*”[tw]
OR “Stereotactic Radiation Therap*”[tw]
OR SBRT[tw]
OR “Radiological therap*”[tw]
OR SABR[tw]
OR “Stereotactic ablative radiotherap*”[tw]
OR SRS[tw]
OR “Stereotactic external beam radiotherap*”[tw]
OR “Cyber Knife”[tw]
OR “Focused radiation therap*”[tw]
OR “Radiosurgical ablation”[tw]
OR “Radiation oncology treatment*”[tw]
OR “External beam therap*”[tw]
OR” Radiochemotherap*”[tw]
OR “Concurrent Chemoradiotherap*”[tw]
OR “Concomitant Chemoradiotherap*”[tw]
OR” Chemoradiotherap*, Concomitant”[tw]
OR “Chemoradiotherap*, Concurrent”[tw]
OR “Synchronous Chemoradiotherap*”[tw]
OR “Chemoradiotherapy, Synchronous” [tiab:~0]
OR “Radiochemotherapy , synchronous” [tiab:~0])

Final search Strategy: 1st Concept OR 2nd Concept

Filters applied: Language German, English and publication: 2005-now

13.945 results as of 22nd of October
