# Supplementary material for: Challenges in recurrent head and neck squamous cell cancer treatment: systematic review and meta-analysis comparing efficacy and toxicity between post-operative and definitive IMRT-based reirradiation
Source: Clin Transl Radiat Oncol. 2025 Oct 25;56:101061. doi: 10.1016/j.ctro.2025.101061 (PMC12630038; doi:10.1016/j.ctro.2025.101061)
Supplement: Supplementary Data 6 [file mmc6.pdf]

Search strategy for Scopus

1st Concept

(TITLE-ABS-KEY(Head and Neck Neoplasm*)
OR TITLE-ABS-KEY(Head And Neck Squamous Cell Carcinoma*)
OR TITLE-ABS-KEY(HNSCC)
OR TITLE-ABS-KEY(Squamous Cell Carcinoma of the Head and Neck)
OR TITLE-ABS-KEY(Carcinoma, Squamous Cell of Head and Neck)
OR TITLE-ABS-KEY(Squamous Cell Carcinoma of Larynx)
OR TITLE-ABS-KEY(Laryngeal Squamous Cell Carcinoma*)
OR TITLE-ABS-KEY(Hypopharyngeal Squamous Cell Carcinoma*)
OR TITLE-ABS-KEY(Oral Squamous Cell Carcinoma*)
OR TITLE-ABS-KEY(Squamous Cell Carcinoma of the Mouth)
OR TITLE-ABS-KEY(Oropharyngeal Squamous Cell Carcinoma*)
OR TITLE-ABS-KEY(Oral Tongue Squamous Cell Carcinoma*)
OR TITLE-ABS-KEY(HNSC)
OR TITLE-ABS-KEY(Squamous Cell Head and Neck Tumor)
OR TITLE-ABS-KEY(SCC of the Head and Neck)
OR TITLE-ABS-KEY(Head and Neck Epithelial Cancer)
OR TITLE-ABS-KEY(Head and Neck Cancer of Squamous Origin)
OR TITLE-ABS-KEY(Head, Neck Neoplasm*)
OR TITLE-ABS-KEY(Neck Cancer*)
OR TITLE-ABS-KEY(Upper Aerodigestive Tract Neoplasm*)
OR TITLE-ABS-KEY(UADT Neoplasm*)
OR TITLE-ABS-KEY(Head Neoplasm*)
OR TITLE-ABS-KEY(Head and neck tumor*)
OR TITLE-ABS-KEY(Craniofacial neoplasm*)
OR TITLE-ABS-KEY(Craniofacial tumor*)
OR TITLE-ABS-KEY(Cervicofacial cancer*)
OR TITLE-ABS-KEY(Orofacial neoplasm*)
OR TITLE-ABS-KEY(Head and neck malignanc*)
OR TITLE-ABS-KEY(Orofacial cancer*)
OR TITLE-ABS-KEY(Craniofacial malignanc*)
OR TITLE-ABS-KEY(Cervicofacial malignanc*))

AND

(TITLE-ABS-KEY(Repeat irradiation*)
OR TITLE-ABS-KEY(Reirradiation)
OR TITLE-ABS-KEY(Re-Irradiation*)
OR TITLE-ABS-KEY(Second irradiation*)
OR TITLE-ABS-KEY(Repeated radiation therap*)
OR TITLE-ABS-KEY(Radiation re-treatment)
OR TITLE-ABS-KEY(Re-radiation*)
OR TITLE-ABS-KEY(Repeated irradiation procedure)
OR TITLE-ABS-KEY(Salvage radiation therap*)
OR TITLE-ABS-KEY(Repeat Concurrent Chemoradiotherap*)
OR TITLE-ABS-KEY(Repeat Concomitant Chemoradiotherap*)
OR TITLE-ABS-KEY(Repeat Chemoradiotherap*, Concomitant)
OR TITLE-ABS-KEY(Repeat Chemoradiotherap*, Concurrent)
OR TITLE-ABS-KEY(Repeat Synchronous Chemoradiotherap*)
OR TITLE-ABS-KEY(Repeat Chemoradiotherap*, Synchronous)
OR TITLE-ABS-KEY(Repeat Radiochemotherap*, synchronous)

OR TITLE-ABS-KEY(Salvage Radiochemotherap*)
OR TITLE-ABS-KEY(Salvage Concurrent Chemoradiotherap*)
OR TITLE-ABS-KEY(Salvage Concomitant Chemoradiotherap*)
OR TITLE-ABS-KEY(Salvage Chemoradiotherap*, Concomitant)
OR TITLE-ABS-KEY(Salvage Chemoradiotherap*, Concurrent)
OR TITLE-ABS-KEY(Salvage Synchronous Chemoradiotherap*)
OR TITLE-ABS-KEY(Salvage Chemoradiotherap*, Synchronous)
OR TITLE-ABS-KEY(Salvage Radiochemotherap*, synchronous))

2nd Concept

(((TITLE-ABS-KEY (recurrence)
OR TITLE-ABS-KEY(return of)
OR TITLE-ABS-KEY (relapse*)
OR TITLE-ABS-KEY(second*))
AND
(TITLE-ABS-KEY (Squamous Cell Carcinoma of Head and Neck"[Mesh]
OR TITLE-ABS-KEY(Head and Neck Neoplasm"[Mesh]
OR TITLE-ABS-KEY(Head And Neck Squamous Cell Carcinoma*)
OR TITLE-ABS-KEY(HNSCC)
OR TITLE-ABS-KEY(Squamous Cell Carcinoma of the Head and Neck)
OR TITLE-ABS-KEY(Carcinoma, Squamous Cell of Head and Neck" [tw]
OR TITLE-ABS-KEY(Squamous Cell Carcinoma of Larynx)
OR TITLE-ABS-KEY(Laryngeal Squamous Cell Carcinoma*)
OR TITLE-ABS-KEY(Hypopharyngeal Squamous Cell Carcinoma*)
OR TITLE-ABS-KEY(Oral Squamous Cell Carcinoma*)
OR TITLE-ABS-KEY(Squamous Cell Carcinoma of the Mouth)
OR TITLE-ABS-KEY(Oropharyngeal Squamous Cell Carcinoma*)
OR TITLE-ABS-KEY(Oral Tongue Squamous Cell Carcinoma*)
OR TITLE-ABS-KEY(HNSC)
OR TITLE-ABS-KEY(Squamous Cell Head and Neck Tumor)
OR TITLE-ABS-KEY(SCC of the Head and Neck)
OR TITLE-ABS-KEY(Head and Neck Epithelial Cancer*)
OR TITLE-ABS-KEY(Head and Neck Cancer of Squamous Origin)
OR TITLE-ABS-KEY(Head, Neck Neoplasm*)
OR TITLE-ABS-KEY(Neck Cancer*)
OR TITLE-ABS-KEY(Upper Aerodigestive Tract Neoplasm*)
OR TITLE-ABS-KEY(UADT Neoplasm*)
OR TITLE-ABS-KEY(Head Neoplasm*)
OR TITLE-ABS-KEY(Head and neck tumor*)
OR TITLE-ABS-KEY(Craniofacial neoplasm*)
OR TITLE-ABS-KEY(Craniofacial tumor*)
OR TITLE-ABS-KEY(Cervicofacial cancer*)
OR TITLE-ABS-KEY(Orofacial neoplasm*)
OR TITLE-ABS-KEY(Head and neck malignanc*)
OR TITLE-ABS-KEY(Orofacial cancer*)
OR TITLE-ABS-KEY(Craniofacial malignanc*)
OR TITLE-ABS-KEY(Cervicofacial malignanc*)))
AND

(TITLE-ABS-KEY (Radiotherapy, Image-Guided)
OR TITLE-ABS-KEY(Radiosurgery"[Mesh]
OR TITLE-ABS-KEY(Radiotherapy, Intensity-Modulated)

OR TITLE-ABS-KEY (Radiotherap*)
OR TITLE-ABS-KEY(Radiation Therap*)
OR TITLE-ABS-KEY(Radiation Treatment*)
OR TITLE-ABS-KEY(Targeted Radiotherap*)
OR TITLE-ABS-KEY(Radiation)
OR TITLE-ABS-KEY(Image-Guided Radiotherap*)
OR TITLE-ABS-KEY(Image Guided Radiation Therap*)
OR TITLE-ABS-KEY (IMRT)
OR TITLE-ABS-KEY(Target Organ Alignment Radiotherapy)
OR TITLE-ABS-KEY(Modulated radiation therap*)
OR TITLE-ABS-KEY(Intensity Modulated radiation therap*)
OR TITLE-ABS-KEY (IMXT)
OR TITLE-ABS-KEY(Intensity-modulated beam therapy)
OR TITLE-ABS-KEY(Conformal radiation therap*)
OR TITLE-ABS-KEY(Intensity Modulated radiation treatment*)
OR TITLE-ABS-KEY(Precision radiation therap*)
OR TITLE-ABS-KEY(Volumetric-Modulated Arc Therap*)
OR TITLE-ABS-KEY(Intensity-Modulated Arc Therap*)
OR TITLE-ABS-KEY(Helical Tomotherap*)
OR TITLE-ABS-KEY(Gamma Knife Radiosurger*)
OR TITLE-ABS-KEY(Stereotactic Radiation*)
OR TITLE-ABS-KEY(Stereotactic Radiosurger*)
OR TITLE-ABS-KEY(Linear Accelerator Radiosurger*)
OR TITLE-ABS-KEY(LINAC Radiosurger*)
OR TITLE-ABS-KEY(Stereotactic Body Radiotherap*)
OR TITLE-ABS-KEY(CyberKnife Radiosurger*)
OR TITLE-ABS-KEY(Stereotactic Radiation Therap*)
OR TITLE-ABS-KEY (SBRT)
OR TITLE-ABS-KEY(Radiological therap*)
OR TITLE-ABS-KEY (SABR)
OR TITLE-ABS-KEY(Stereotactic ablative radiotherap*)
OR TITLE-ABS-KEY(SRS)
OR TITLE-ABS-KEY(Stereotactic external beam radiotherap*)
OR TITLE-ABS-KEY(Cyber Knife)
OR TITLE-ABS-KEY(Focused radiation therap*)
OR TITLE-ABS-KEY(Radiosurgical ablation)
OR TITLE-ABS-KEY(Radiation oncology treatment*)
OR TITLE-ABS-KEY(External beam therap*)
OR TITLE-ABS-KEY(Radiochemotherap*)
OR TITLE-ABS-KEY(Concurrent Chemoradiotherap*)
OR TITLE-ABS-KEY(Concomitant Chemoradiotherap*)
OR TITLE-ABS-KEY(Chemoradiotherap*, Concomitant)
OR TITLE-ABS-KEY(Chemoradiotherap*, Concurrent)
OR TITLE-ABS-KEY(Synchronous Chemoradiotherap*)
OR TITLE-ABS-KEY(Chemoradiotherap*, Synchronous)
OR TITLE-ABS-KEY(Radiochemotherap* , synchronous))

Final search Strategy: 1st Concept OR 2nd Concept

Filters applied: Language German, English and publication: 2005-now
