# Supplementary material for: Challenges in recurrent head and neck squamous cell cancer treatment: systematic review and meta-analysis comparing efficacy and toxicity between post-operative and definitive IMRT-based reirradiation
Source: Clin Transl Radiat Oncol. 2025 Oct 25;56:101061. doi: 10.1016/j.ctro.2025.101061 (PMC12630038; doi:10.1016/j.ctro.2025.101061)
Supplement: Supplementary Data 7 [file mmc7.pdf]

Search strategy for Web of science.

All databases provided by WoS except MEDLINE are selected.

1st Concept

((TS=(Head and Neck Neoplasm*)
OR TS=(Head And Neck Squamous Cell Carcinoma*)
OR TS=(HNSCC)
OR TS=(Squamous Cell Carcinoma of the Head and Neck)
OR TS=(Carcinoma, Squamous Cell of Head and Neck)
OR TS=(Squamous Cell Carcinoma of Larynx)
OR TS=(Laryngeal Squamous Cell Carcinoma*)
OR TS=(Hypopharyngeal Squamous Cell Carcinoma*)
OR TS=(Oral Squamous Cell Carcinoma*)
OR TS=(Squamous Cell Carcinoma of the Mouth)
OR TS=(Oropharyngeal Squamous Cell Carcinoma*)
OR TS=(Oral Tongue Squamous Cell Carcinoma*)
OR TS=(HNSC)
OR TS=(Squamous Cell Head and Neck Tumor)
OR TS=(SCC of the Head and Neck)
OR TS=(Head and Neck Epithelial Cancer)
OR TS=(Head and Neck Cancer of Squamous Origin)
OR TS=(Head, Neck Neoplasm*)
OR TS=(Neck Cancer*)
OR TS=(Upper Aerodigestive Tract Neoplasm*)
OR TS=(UADT Neoplasm*)
OR TS=(Head Neoplasm*)
OR TS=(Head and neck tumor*)
OR TS=(Craniofacial neoplasm*)
OR TS=(Craniofacial tumor*)
OR TS=(Cervicofacial cancer*)
OR TS=(Orofacial neoplasm*)
OR TS=(Head and neck malignanc*)
OR TS=(Orofacial cancer*)
OR TS=(Craniofacial malignanc*)
OR TS=(Cervicofacial malignanc*))
AND
(TS=(Repeat irradiation*)
OR TS=(Reirradiation)
OR TS=(Re-Irradiation*)
OR TS=(Second irradiation*)
OR TS=(Repeated radiation therap*)
OR TS=(Radiation re-treatment)
OR TS=(Re-radiation*)
OR TS=(Repeated irradiation procedure)
OR TS=(Salvage radiation therap*)
OR TS=(Repeat Concurrent Chemoradiotherap*)
OR TS=(Repeat Concomitant Chemoradiotherap*)
OR TS=(Repeat Chemoradiotherap*, Concomitant)
OR TS=(Repeat Chemoradiotherap*, Concurrent)
OR TS=(Repeat Synchronous Chemoradiotherap*)
OR TS=(Repeat Chemoradiotherap*, Synchronous)
OR TS=(Repeat Radiochemotherap*, synchronous)
OR TS=(Salvage Radiochemotherap*)
OR TS=(Salvage Concurrent Chemoradiotherap*)

OR TS=(Salvage Concomitant Chemoradiotherap*)
OR TS=(Salvage Chemoradiotherap*, Concomitant)
OR TS=(Salvage Chemoradiotherap*, Concurrent)
OR TS=(Salvage Synchronous Chemoradiotherap*)
OR TS=(Salvage Chemoradiotherap*, Synchronous)
OR TS=(Salvage Radiochemotherap*, synchronous)))

2nd Concept

((((TS= (recurrence)
OR TS=(return of)
OR TS= (relapse*)
OR TS=(second*))
AND
(TS= (Squamous Cell Carcinoma of Head and Neck"[Mesh]
OR TS=(Head and Neck Neoplasm"[Mesh]
OR TS=(Head And Neck Squamous Cell Carcinoma*)
OR TS=(HNSCC)
OR TS=(Squamous Cell Carcinoma of the Head and Neck)
OR TS=(Carcinoma, Squamous Cell of Head and Neck" [tw]
OR TS=(Squamous Cell Carcinoma of Larynx)
OR TS=(Laryngeal Squamous Cell Carcinoma*)
OR TS=(Hypopharyngeal Squamous Cell Carcinoma*)
OR TS=(Oral Squamous Cell Carcinoma*)
OR TS=(Squamous Cell Carcinoma of the Mouth)
OR TS=(Oropharyngeal Squamous Cell Carcinoma*)
OR TS=(Oral Tongue Squamous Cell Carcinoma*)
OR TS=(HNSC)
OR TS=(Squamous Cell Head and Neck Tumor)
OR TS=(SCC of the Head and Neck)
OR TS=(Head and Neck Epithelial Cancer*)
OR TS=(Head and Neck Cancer of Squamous Origin)
OR TS=(Head, Neck Neoplasm*)
OR TS=(Neck Cancer*)
OR TS=(Upper Aerodigestive Tract Neoplasm*)
OR TS=(UADT Neoplasm*)
OR TS=(Head Neoplasm*)
OR TS=(Head and neck tumor*)
OR TS=(Craniofacial neoplasm*)
OR TS=(Craniofacial tumor*)
OR TS=(Cervicofacial cancer*)
OR TS=(Orofacial neoplasm*)
OR TS=(Head and neck malignanc*)
OR TS=(Orofacial cancer*)
OR TS=(Craniofacial malignanc*)
OR TS=(Cervicofacial malignanc*)))
AND
(TS= (Radiotherapy, Image-Guided)
OR TS=(Radiosurgery)
OR TS=(Radiotherapy, Intensity-Modulated)
OR TS= (Radiotherap*)
OR TS=(Radiation Therap*)
OR TS=(Radiation Treatment*)

OR TS=(Targeted Radiotherap*)
OR TS=(Radiation)
OR TS=(Image-Guided Radiotherap*)
OR TS=(Image Guided Radiation Therap*)
OR TS= (IMRT)
OR TS=(Target Organ Alignment Radiotherapy)
OR TS=(Modulated radiation therap*)
OR TS=(Intensity Modulated radiation therap*)
OR TS= (IMXT)
OR TS=(Intensity-modulated beam therapy)
OR TS=(Conformal radiation therap*)
OR TS=(Intensity Modulated radiation treatment*)
OR TS=(Precision radiation therap*)
OR TS=(Volumetric-Modulated Arc Therap*)
OR TS=(Intensity-Modulated Arc Therap*)
OR TS=(Helical Tomotherap*)
OR TS=(Gamma Knife Radiosurger*)
OR TS=(Stereotactic Radiation*)
OR TS=(Stereotactic Radiosurger*)
OR TS=(Linear Accelerator Radiosurger*)
OR TS=(LINAC Radiosurger*)
OR TS=(Stereotactic Body Radiotherap*)
OR TS=(CyberKnife Radiosurger*)
OR TS=(Stereotactic Radiation Therap*)
OR TS= (SBRT)
OR TS=(Radiological therap*)
OR TS= (SABR)
OR TS=(Stereotactic ablative radiotherap*)
OR TS=(SRS)
OR TS=(Stereotactic external beam radiotherap*)
OR TS=(Cyber Knife)
OR TS=(Focused radiation therap*)
OR TS=(Radiosurgical ablation)
OR TS=(Radiation oncology treatment*)
OR TS=(External beam therap*)
OR TS=(Radiochemotherap*)
OR TS=(Concurrent Chemoradiotherap*)
OR TS=(Concomitant Chemoradiotherap*)
OR TS=(Chemoradiotherap*, Concomitant)
OR TS=(Chemoradiotherap*, Concurrent)
OR TS=(Synchronous Chemoradiotherap*)
OR TS=(Chemoradiotherap*, Synchronous)
OR TS=(Radiochemotherap* , synchronous)))

Final search Strategy: 1st Concept OR 2nd Concept

Filters applied: Language German, English and publication: 2005-now
