# Supplementary material for: Challenges in recurrent head and neck squamous cell cancer treatment: systematic review and meta-analysis comparing efficacy and toxicity between post-operative and definitive IMRT-based reirradiation
Source: Clin Transl Radiat Oncol. 2025 Oct 25;56:101061. doi: 10.1016/j.ctro.2025.101061 (PMC12630038; doi:10.1016/j.ctro.2025.101061)
Supplement: Supplementary Data 9 [file mmc9.docx]

Guidelines for title and abstract screening

Eligible for full-text retrieval

- Report, trial, study, or comment on radiotherapy for recurrent or metastatic head and neck cancer
- Report, trial, study, or comment on treatment for recurrent or metastatic head and neck cancer if it is unclear in the abstract whether radiation was applied
- Report, trial, study, or comment on treatment for recurrent or metastatic head and neck cancer with proton therapy, or other methods if it was suspected that IMRT-and SBRT-based radiation was also applied (i.e., if a comparator cohort was mentioned)
- Report, trial, study, or comment on the pattern of failure, incidence, risk stratification, and surveys on clinical practice in the setting of recurrent head and neck cancer
- Report, trial, study, or comment on quality of life after radiotherapy or multimodal treatment for recurrent or metastatic head and neck cancer
- Dosimetry reports where survival data was suspected to be presented as well

Not eligible

- Report, trial, study, or comments specifically on nasopharyngeal head and neck cancer where no comparator cohort is suspected
- Report, trial, study, or comments specifically on skull base cancer where no comparator cohort is suspected
- Case reports or case series where it was clear that fewer than 10 patients were presented
- Report, trial, study, or comments specifically on primary/first diagnosed head and neck cancer, where no recurrent cases are suspected
- Guidelines, reviews where no original patient data was expected to be presented
- Any work on other cancers
- Any work that otherwise likely does not meet the inclusion criteria stated in the protocol, like histo-pathological work, scientific reports on genetics
