# Supplementary material for: Challenges in recurrent head and neck squamous cell cancer treatment: systematic review and meta-analysis comparing efficacy and toxicity between post-operative and definitive IMRT-based reirradiation
Source: Clin Transl Radiat Oncol. 2025 Oct 25;56:101061. doi: 10.1016/j.ctro.2025.101061 (PMC12630038; doi:10.1016/j.ctro.2025.101061)
Supplement: Supplementary Data 10 [file mmc10.docx]

| **Author** | **Title, Journal (Year)** | **Assessment** |
| --- | --- | --- |
| Abbasi | "Reirradiation of Head and Neck Cancers with Imrt." International Journal of Radiation Oncology Biology Physics 96.3 (2016) | Comment. No patient data reported |
| Abu Jawad | "Re-Irradiation of Tumor Recurrence in Head-Neck Area by Helical Tomotherapy." Strahlentherapie Und Onkologie 186 (2010) | only included 12 patients (at least 10 must be included in each arm), no info about 1-year OS |
| Adam | "Combining External Beam Radiotherapy and Radiopharmaceutical Therapy Dosimetry for Recurrent Head and Neck Cancer Treatment" Original Disseration (2022) | Radiotherapy in combination with I131 Brachytherapy, thus not allowed co-intervention |
| Aguirre | "Survival Outcomes of Re-Irradiation with Intensity Modulated Radiation Therapy (Imrt) for Recurrent Head and Neck Squamous Cell Carcinoma." International Journal of Radiation Oncology Biology Physics 87.2 (2013) | results from patient with adjuvant and definitive radiotherapy were not reported separately, hence no comparison between aIMRT and dIMRT could be made |
| Ahlawat | "Tumor Volume as Prognostic Factor for Recurrent Head and Neck Cancers Treated with Re-Irradiation." Radiotherapy and Oncology 161 (2021) | Missing info on tumor localisation, only included unresected cancer, no comparison to aIMRT was possible |
| Ahlawat | "Reirradiation with Imrt for Recurrent Head and Neck Cancer: A Single-Institutional Report on Disease Control, Survival and and Toxicity." Rep Pract Oncol Radiother 22.4 (2017) | surgery was found as significant factor but no separate analysis for 1- and 2-year OS or other endpoints of interest was done |
| Ahn | "Risk Factors for Subsequent Development of Distant Metastatic Disease in a Cohort of Patients Treated with Reirradiation for Recurrent Head and Neck Cancer." International Journal of Radiation Oncology Biology Physics 96.2 (2016) | unclear if IMRT was used (2-2,5Gy/fraction) and GTV=PTV, no localisation stated, no endpoint of interest stated for aIMRT and dIMRT, separatly |
| Akmansu | "Cetuximab Concomitant with Second-Line Radiation Therapy in Patients with Locally Advanced Recurrent Squamous Cell Head and Neck Cancer." Case Rep Oncol 3.3 (2010) | only 9 patients reported (at least 10 patients in each arm required) |
| Altay-Langguth | "Re-Irradiation with Concurrent and Maintenance Nivolumab in Locally Recurrent and Inoperable Squamous Cell Carcinoma of the Head and Neck: A Single-Center Cohort Study." Clin Transl Radiat Oncol 28 (2021) | only reports outcomes for definitive IMRT, no comparison to aIMRT possible |
| Al-Wassia | "A Retrospective Study of Head and Neck Re-Irradiation for Patients with Recurrent or Second Primary Head and Neck Cancer: The Mcgill University Experience." J Otolaryngol Head Neck Surg 44.1 (2015). | 26% Nasopharyngeal Cancer (max 20% allowed), 44% adjuvant radiotherapy, results from patient with adjuvant and definitive radiotherapy were not reported separately, hence no comparison between aIMRT and dIMRT could be made |
| Amdal | "Impact of Hyperfractionated Re-Irradiation on Quality of Life in Patients with Recurrent or Second Primary Head and Neck Cancer a Prospective Single Institutional Study." Clin Transl Radiat Oncol 42 (2023) | no endpoint of interest stated (only reports median survival for aIMRT and dIMRT but not 1-year and 2-year OS) |
| Appold | "High Dose Hyperfractionated Re-Irradiation of Head and Neck Tumors." Radiotherapy and Oncology 81 (2006)´ | 41% adjuvant radiotherapy, results from patient with adjuvant and definitive radiotherapy were not reported separately, hence no comparison between aIMRT and dIMRT could be made |
| Appold | "Feasibility of high-dose hyperfractionated re-irradiation in patient with recurrent or second head and neck tumors" Radiotherapy and Oncology (2007) | 46% adjuvant radiotherapy, results from patient with adjuvant and definitive radiotherapy were not reported separately, hence no comparison between aIMRT and dIMRT could be made |
| Argiris | "A New Prognostic Model in Chemotherapy-Treated Patients with Recurrent or Metastatic Head and Neck Cancer: An Analysis of ECOG-ACRIN E1305." Eur J Cancer (2024) | no radiotherapy applied |
| Argiris | "Phase I Trial of Pemetrexed in Combination with Cetuximab and Concurrent Radiotherapy in Patients with Head and Neck Cancer." Ann Oncol 22.11 (2011) | only 9 patients re-irradiated, (at least 10 required) |
| Awan | "Final results of a multi-institutional phase II trial of reirradiation with concurrent weekly cisplatin and cetuximab for recurrent or second primary squamous cell carcinoma of the head and neck" Ann Oncol (2018) | included |
| Babiker | "Phase I Trial of Cemiplimab, Radiotherapy, Cyclophosphamide and Granulocyte Macrophage Colony-Stimulating Factor in Patients with Recurrent or Metastatic Head and Neck Squamous Cell Carcinoma." Oncologist 26.9 (2021) | no info on Cancer Site, Histology, only including 15 patients (at least 10 patients per arm are required) |
| Bagley | "Highly Conformal Reirradiation in Patients with Prior Oropharyngeal Radiation: Clinical Efficacy and Toxicity Outcomes." Head Neck 42.11 (2020) | includes both IMRT and SBRT cohort, 32% adjuvant IMRT without comparison between dIMRT and aIMRT |
| Bahl | "Evaluation of Reirradiation in Locally Advanced Head and Neck Cancers: Toxicity and Early Clinical Outcomes." J Oncol (2018) | No info about cancer histology, only includes definitive IMRT, no comparison to aIMRT possible |
| Balermpas | "Combined Cetuximab and Reirradiation for Locoregional Recurrent and Inoperable Squamous Cell Carcinoma of the Head and Neck." Strahlenther Onkol 185.12 (2009) | only 7 patients included, (at least 10 required per arm) |
| Balermpas | "Reirradiation with Cetuximab in Locoregional Recurrent and Inoperable Squamous Cell Carcinoma of the Head and Neck: Feasibility and First Efficacy Results." Int J Radiat Oncol Biol Phys 83.3 (2012) | 3D Conformal radiotherapy used (ineligible re-irradiation technique) |
| Balermpas | "Re-Irradiation in Combination with Cetuximab in the Treatment of Recurrent Hno-Squamous." Strahlentherapie Und Onkologie 185 (2009) | only 8 patients included, (at least 10 required per arm) |
| Balermpas | "Re-irradiation in combination with Cetuximab in the treatment of metastatic squamous cell carcinoma in the Head-Neck area (Update)" Strahlentherapie Und Onkologie 186 (2010) | 3D Conformal radiotherapy used, only |
| Barros | "Re-Irradiation in Squamous Cell Carcinoma of the Head and Neck." Forum of Clinical Oncology 13.3 (2022) | 26% adjuvant radiotherapy, results from patient with adjuvant and definitive radiotherapy were not reported separately, hence no comparison between aIMRT and dIMRT could be made |
| Batham | "To Analyze the Feasibility, Effectiveness and Toxicity of Re-Irradiation in Head and Neck Cancers." Radiotherapy and Oncology 152 (2020) | 93% adjuvant radiotherapy, only 5 definitively treated patients (at least 10 required per arm) |
| Bauman | "FIERCE-HN: a Multicenter, Randomized, Placebo-controlled, Phase 3 Study of ficlatuzumab + cetuximab in pts w/ recurrent or Metastatic (R/M) HPV-negative Head and Neck Squamous Cell Carcinoma (HNSCC)" International journal of radiation oncology biology physics(2024) | Study protocol only |
| Bauman | "Randomized Phase Ii Trial of Ficlatuzumab with or without Cetuximab in Pan-Refractory Recurrent/Metastatic Head and Neck Cancer." Journal of Clinical Oncology 41.22 (2023) | no radiotherapy applied |
| Becherini | "Hypofractionated Re-Irradiation for Locoregionally Recurrent Head and Neck Cancer." Radiotherapy and Oncology 182 (2023) | Tumor localisation unknown, SBRT was used only |
| Beddok | "Predictors of Toxicity after Curative Reirradiation with Intensity Modulated Radiotherapy or Proton Therapy for Recurrent Head and Neck Carcinoma: New Dose Constraints for Pharyngeal Constrictors Muscles and Oral Cavity." Strahlenther Onkol 199.10 (2023): | 30,5% nasopharyngeal Carcinoma (max 20% allowed), 47% proton therapy (max 30% allowed) |
| Beddok | "Curative High-Dose Reirradiation for Patients with Recurrent Head and Neck Squamous Cell Carcinoma Using Imrt or Proton Therapy: Outcomes and Analysis of Patterns of Failure." Head Neck 44.11 (2022) | 39% adjuvant radiotherapy, results from patient with adjuvant and definitive radiotherapy were not reported separately, hence no comparison between aIMRT and dIMRT could be made |
| Berger | "Reirradiation with Alternating Docetaxel-Based Chemotherapy for Recurrent Head and Neck Squamous Cell Carcinoma." Strahlentherapie Und Onkologie 186.5 (2010) | 18% of patients received IMRT (at least 70% required), results from patient with adjuvant and definitive radiotherapy were not reported separately, hence no comparison between aIMRT and dIMRT could be made |
| Biagioli | "Re-Irradiation of Recurrent Head and Neck Cancers with Imrt Technique and Concurrent Chemotherapy." International Journal of Radiation Oncology Biology Physics 63.2 (2005) | 45% adjuvant IMRT, results from patient with adjuvant and definitive radiotherapy were not reported separately, hence no comparison between aIMRT and dIMRT could be made |
| Biagioli | "Intensity-modulated radiotherapy with concurrent chemotherapy for previously irradiated, recurrent head and neck cancer" Int J Radiat Oncol Biol Phys (2007) | included |
| Biau | "Reirradiation for Head and Neck Squamous Cell Carcinoma: Indications and Results." Cancer/Radiotherapie 23.6-7 (2019) | review only, no original patient data presented |
| Blanchard | "Reirradiation in head and neck cancer" Radiotherapy and Oncology (2021) | review only, no original patient data presented |
| Bonomo | "Salvage stereotactic re-irradiation with CyberKnife for locally recurrent head and neck cancer: a single center experience" Tumori (2014) | definitive SBRT only |
| Bonomo | "Stereotactic re-irradiation for recurrent head and neck cancer: predictive factors of efficacy from a single-institution experience" European Journal of Cancer (2017) | definitive SBRT only |
| Bots | "Re-Irradiation for Head and Neck Tumors: Efficacy Versus Late Toxicity in 137 Patients." Radiotherapy and Oncology 119 (2016) | 44% IMRT, results from patient with adjuvant and definitive radiotherapy were not reported separately, hence no comparison between aIMRT and dIMRT could be made |
| Bots | "Reirradiation of Head and Neck Cancer: Long-Term Disease Control and Toxicity." Head Neck 39.6 (2017) | in the post-operative group, IMRT was used in 49% of patients (at least 70% required), in definitive group, only 24% received IMRT |
| Bratland | "REPORT; REirradiation and PD-1 blockade On Recurrent squamous cell head and neck Tumors" Radiotherapy and Oncology 161 (2021) | unclear how many patients received salvage surgery, no endpoint of interest stated |
| Bruce | "Safety and Toxicity of Lopofosine 1-131 with External Beam Radiation Therapy (Ebrt) in Recurrent or Metastatic Head and Neck Cancer (Hnc): Results of a Phase 1 Study." International Journal of Radiation Oncology Biology Physics 118.5 (2024) | CLR 131 with EBRT, thus ineligible co-intervention applied |
| Bruce | "Safety and toxicity of Iopofosine I 131 (CLR 131) with external beam radiation therapy in recurrent or metastatic head and neck cancer: results of a phase 1 single-centre, open-label, single-arm, dose escalation and dose expansion study" eBioMedicine (2025) | CLR 131 and IMRT, thus ineligible co-intervention applied |
| Buglione | "Reirradiation in Head and Neck Recurrent or Second Primary Tumor: Efficacy, Safety and Prognostic Factors." Tumori 101.5 (2015) | SBRT vs non-SBRT. In the non-SBRT group 83% received IMRT-based re-irradiation. In the whole cohort 11 patients underwent resection. results from patient with adjuvant and definitive radiotherapy were not reported separately, hence no comparison between aIMRT and dIMRT could be made. |
| Bush | "Long-Term Outcomes for Re-Irradiation of Recurrent Head-and-Neck Cancers: Mature Data from a Large Series." International Journal of Radiation Oncology Biology Physics 102.3 (2018) | SBRT was used, only |
| Cacicedo Fernandez de Bobadilla | "Re-Irradiation for Recurrent Head and Neck Carcinomas (by Dr. Peponi et al.)." J buon (2013) | Correspondence, no endpoint of interest stated |
| Campbell | "Head and Neck Stereotactic Body Radiation Therapy ReIrradiation for Patients With Carotid or Skin Involvement Ineligible for RTOG 3507" International Journal of Radiation Oncology Biology Physics (2021) | No info on cancer localisation, SBRT was used |
| Castro | "Recurrent Oropharyngeal Squamous Cell Carcinomas Maintain Anti-Tumor Immunity and Multinucleation Levels Following Completion of Radiation." Head Neck Pathol 17.4 (2023) | Immuno-histochemical analysis, no endpoint of interest stated |
| Caudell | "Multi-Institution Analysis of Intensity Modulated Radiation Therapy-Based Reirradiation for Head and Neck Cancer: Improved Risk-Benefit Profile in the Modern Era." International Journal of Radiation Oncology Biology Physics 96.2 (2016) | Includes both aIMRT and dIMRT. Only 2-year OS for dIMRT was stated separately. No comparison between aIMRT and dIMRT possible. Overlap to Ward et al. |
| Caudell | "Volume, Dose, and Fractionation Considerations for IMRT-based Reirradiation in Head and Neck Cancer: A Multi-institution Analysis" Int J Radiat Oncol Biol Phys 100.3 (2018) | analysis of toxicity in post-operative and definitive IMRT, overlap to Ward et al. 2018 |
| Chang | "Locoregionally Recurrent Head and Neck Squamous Cell Carcinoma: Incidence, Survival, Prognostic factors and Treatment Outcomes." Oncotarget 8.33 (2017) | unclear how many patients of group "Surgery +/-(Chemo)radiotherapy" received re-irradiation |
| Chatterjee | "Second primary cancers: Incidence and outcomes from a population of patients accrued in six prospective controlled trials at a tertiary cancer institute" Indian J Cancer (2024) | Oesophageal and head and neck cancer included, no exact info on re-treatment modality |
| Chen | "Magnetic Resonance Imaging Guided Reirradiation of Recurrent and Second Primary Head and Neck Cancer." Adv Radiat Oncol 2.2 (2017) | only 6 IMRT and 7 SBRT patients included |
| Chen | "Prospective Trial of High-Dose Reirradiation Using Daily Image Guidance with Intensity-Modulated Radiotherapy for Recurrent and Second Primary Head-and-Neck Cancer." Int J Radiat Oncol Biol Phys 80.3 (2011) | only reports on definitive IMRT, no comparison to aIMRT possible |
| Chen | "Re-Irradiation Versus Systemic Therapy for the Management of Local-Regionally Recurrent Head and Neck Cancer." Radiother Oncol 196 (2024) | only reports on definitive IMRT, no comparison to aIMRT possible |
| Chen | "Functional and Quality-of-Life Outcomes after Reirradiation for Head and Neck Cancer." Laryngoscope 124.8 (2014) | Quality of life analysis, no endpoint of interest stated |
| Chen | "Dose Sparing of Brainstem and Spinal Cord for Re-Irradiating Recurrent Head and Neck Cancer with Intensity-Modulated Radiotherapy." Med Dosim 36.1 (2011) | includes only 7 patients (at least 10 required) |
| Chen | "Re-irradiation with concurrent pembrolizumab for local-regionally recurrent squamous cell carcinoma of the head and neck" Oral Oncology Reports (2025) | 16 patients, only definitive re-irradiation applied, no comparison possible |
| Chen | "Outcomes of re-irradiation for oral cavity squamous cell carcinoma" Biomed J (2022) Included | included |
| Chin | "Salvage Re-Irradiation Using Intensity-Modulated Radiotherapy for Squamous Cell Carcinoma of the Head and Neck." International Journal of Radiation Oncology Biology Physics 72.1 (2008) | 30% nasopharyngeal cancer (max 20% allowed), four cases with SBRT boost, thus ineligible co-intervention |
| Choe | "Prior Chemoradiotherapy Adversely Impacts Outcomes of Recurrent and Second Primary Head and Neck Cancer Treated with Concurrent Chemotherapy and Reirradiation." Cancer 117.20 (2011) | unknown if IMRT was used for re-irradiation, surgery was significant factor but no 1-year and 2-Year survival stated |
| Choe | "Previous Chemoradiotherapy Predicts for Worse Survival in Patients Undergoing Chemo-Reirradiation for Recurrent and Second Primary Head and Neck Cancer." International Journal of Radiation Oncology Biology Physics 72.1 (2008) | unknown if IMRT was used for re-irradiation, results from patient with adjuvant and definitive radiotherapy were not reported separately, hence no comparison between aIMRT and dIMRT could be made |
| Choi | "Outcomes of Salvage Therapy for Oropharyngeal Cancer Recurrence Following Upfront Radiation Therapy and Prognostic Factors." Cancer Res Treat 55.4 (2023) | Missing info on tumor localisation, no clear statement about endpoints of interest |
| Choi | "Re-Irradiation Using Intensity-Modulated Radiotherapy for Recurrent and Second Primary Head and Neck Cancer." Anticancer Res 38.5 (2018) | 38% adjuvant IMRT, 23% nasopharyngeal cancer (max 20% allowed) |
| Curtis | "Outcomes of patients with loco-regionally recurrent or new primary squamous cell carcinomas of the head and neck treated with curative intent reirradiation at Mayo Clinic" Radiat Oncol (2016) | included |
| Cohen | "Phase I Trial of Tirapazamine, Cisplatin and Concurrent Accelerated Boost Reirradiation in Patients with Recurrent Head and Neck Cancer." Int J Radiat Oncol Biol Phys 67.3 (2007) | Only using 3D-Conformal Radiation, only including unresected patients, no comparison to post-operative treatment possible |
| Colantonio | "Re-Irradiation with Cetuximab in Relapsed Squamous Cell Carcinoma of the Head and Neck (Hnc)." Ejc Supplements 7.2 (2009) | only 9 patients included (at least 10 patients must be in each arm) |
| Conley | "Proteasome Inhibitor Bortezomib & Re-Irradiation (Rrt) with Scheduled Treatment Break in Patients (Pts) with Head/Neck Squamous Carcinoma (Hnscc)." Journal of Clinical Oncology 23.16 (2005) | only 6 included (at least 10 patients must be in each arm) |
| Creak | "Treatment of recurrent head and neck cancer: re-irradiation or chemotherapy?" Clin Oncol (R Coll Radiol) 17.3 (2005) | Review no original patient data on re-irradiation presented |
| Cvek | “Hyperfractionated stereotactic reirradiation for recurrent head and neck cancer” Strahlenther Onkol (2016) | Only including definitive radiotherapy. Twice daily administered fraction of 3 Gy, each. This dose was too high. |
| Dale | "Clinical Outcomes after Re-Irradiation with Dose Painting of Head and Neck Cancer." Radiotherapy and Oncology 182 (2023) | reports only on definitive IMRT |
| de Ridder | "Disease Course after the First Recurrence of Head and Neck Squamous Cell Carcinoma Following (Chemo)Radiation." Eur Arch Otorhinolaryngol 277.1 (2020) | unlear, what salvage therapy was applied, Results from patient with adjuvant and definitive radiotherapy were not reported separately, hence no comparison between aIMRT and dIMRT could be made |
| de Ridder | "Recurrent Oropharyngeal Cancer after Organ Preserving Treatment: Pattern of Failure and Survival." Eur Arch Otorhinolaryngol 274.3 (2017) | all IMRT re-irradiation, not clear what additional salvage therapy applied, Results from patient with adjuvant and definitive radiotherapy were not reported separately, hence no comparison between aIMRT and dIMRT could be made |
| Deeken | "Effect of Multimodality Treatment on Overall Survival for Patients with Metastatic or Recurrent Hpv-Positive Head and Neck Squamous Cell Carcinoma." Head Neck 37.5 (2015) | only between 64 and 83% overlapping volumes (90% at least required) |
| DePaoli | "Regional Recurrence after Carotid Sparing Imrt for Early Stage Glottic Cancer." Pract Radiat Oncol 13.2 (2023) | Case Report only |
| Diaz Gomez | "Efficacy of Reirradiation in Head and Neck Cancer." European Journal of Cancer 49 (2013) | unclear, if IMRT was used, cancer site and % of surgery unknown, Results from patient with adjuvant and definitive radiotherapy were not reported separately, hence no comparison between aIMRT and dIMRT could be made |
| Dibs | "Carotid Blowout after Standard Fractionation Re-Irradiation: The Ohio State University Experience." International Journal of Radiation Oncology Biology Physics 118.5 (2024) | 76% surgery, 13% Intra-operative radiotherapy, histology unknown, Results from patient with adjuvant and definitive radiotherapy were not reported separately, hence no comparison between aIMRT and dIMRT could be made |
| Dibs | "Brachial Plexus Tolerance to Standard Fractionation Reirradiation: The Ohio State University Experience." International Journal of Radiation Oncology Biology Physics 117.2 (2023) | 13% intra-operative radiotherapy, 80% adjuvant radiotherapy, Results from patient with adjuvant and definitive radiotherapy were not reported separately, hence no comparison between aIMRT and dIMRT could be made |
| Dornoff | "Re-Irradiation with Cetuximab or Cisplatin-Based Chemotherapy for Recurrent Squamous Cell Carcinoma of the Head and Neck." Strahlenther Onkol 191.8 (2015) | unclear, how many received IMRT - see correspondence, only 6 patients treated post-operatively (at least 10 needed) |
| Du | "Nivolumab for Recurrent or Metastatic Head and Neck Squamous Cell Carcinoma: A Retrospective Tertiary Centre's Real-World Experience." Curr Oncol 30.10 (2023) | no radiotherapy was used |
| Duprez | "High-Dose Reirradiation with Intensity-Modulated Radiotherapy for Recurrent Head-and-Neck Cancer: Disease Control Survival and Toxicity." Radiother Oncol 111.3 (2014) | no sufficient comparison between aIMRT and dIMRT |
| Duprez | "Intensity-Modulated Radiotherapy for Recurrent and Second Primary Head and Neck Cancer in Previously Irradiated Territory." Radiother Oncol 93.3 (2009) | no sufficient comparison between aIMRT and dIMRT |
| Embring | "Doses and Overlapping Volumes in Re-Irradiation for Head and Neck Cancer." Radiotherapy and Oncology 152 (2020) | no info about tumor localisation, Results from patient with adjuvant and definitive radiotherapy were not reported separately, hence no comparison between aIMRT and dIMRT could be made |
| Embring | "Re-Irradiation for Head and Neck Cancer: Cumulative Dose and the Correlation to Carotid Blowout." Radiotherapy and Oncology 161 (2021) | some patients received Brachytherapy after IMRT, this co-intervention was not allowed |
| Embring | "Re-irradiation for head and neck cancer: Cumulative dose and the correlation to carotid blowout" Radiotherapy and Oncology (2021) | some patients received Brachytherapy after IMRT, this co-intervention was not allowed |
| Embring | "Overlapping Volumes in Re-Irradiation for Head and Neck Cancer - an Important Factor for Patient Selection." Radiat Oncol 15.1 (2020) | some patients received Brachytherapy after IMRT, this co-intervention was not allowed |
| Engelmann | "Re-Irradiation with Curative Intent of Squamous Cell Carcinomas of the Head and Neck in Denmark." Radiotherapy and Oncology 122 (2017) | Tumor localisation not clearly stated, comparison to aIMRT possible |
| Eswaran | "Toxicity and Response Assessment with Prolonged Infusion Gemcitabine and Gefitinib with Intensity Modulated Radiation Therapy in Recurrent Head-and-Neck Squamous Cell Carcinoma -- a Pilot Study." International Journal of Radiation Oncology, Biology Physics 84.3 (2012) | unclear how many patients received adjuvant radiotherapy, only 18 patients included (at least 10 required in each arm) |
| Fong | "Patterns of Recurrence in Hnscc Patients Treated Definitively with Upfront Surgery Chemoradiation." European Archives of Oto-Rhino-Laryngology 281.5 (2024) | no info about treatment of recurrent cancers |
| Fujisawa | "Long-Term Outcomes of Patients with Oral Cavity Cancer Receiving Postoperative Radiotherapy after Salvage Neck Dissection for Cervical Lymph Node Recurrence." Head Neck 46.3 (2024) | only adjuvant radiotherapy, no comparison to dIMRT possible |
| Fury | "A Phase Ii Study of Su5416 in Patients with Advanced or Recurrent Head and Neck Cancers." Invest New Drugs 25.2 (2007) | No IMRT-based radiotherapy used |
| Garg | "Reirradiation for Second Primary or Recurrent Cancers of the Head and Neck: Dosimetric and Outcome Analysis." Head Neck 38 Suppl 1.Suppl 1 (2016). | Results from patient with adjuvant and definitive radiotherapy were not reported separately, hence no comparison between aIMRT and dIMRT could be made |
| Gogineni | "Quality of Life Outcomes Following Organ-Sparing SBRT in Previously Irradiated Recurrent Head and Neck Cancer" Front Oncol (2019) | SBRT was used only, comparison adjuvant SBRT vs definitive |
| Goldstein | "Outcomes Following Reirradiation of Patients with Head and Neck Cancer." Head Neck 30.6 (2008) | unclear if IMRT was used, compares curative and palliative intent - no comparison adjuvant and definitive |
| Gondi | "Reirradiation after osteocutaneous free flap reconstruction for locally recurrent head and neck cancer" Am J Otolaryngol 45.5 (2024) | all adjuvant radiotherapy, only including 5 patients included (at least 10 required) |
| Gondi | "Re-irradiation After Osteocutaneous Free Flap Reconstruction for Locally Recurrent Head and Neck Cancer: A Case Series" International Journal of Radiation Oncology Biology Physics (2024) | all adjuvant radiotherapy after osteocutaneous free flap, 6 patients included at least 10 required) |
| Gorphe | "Disease-Free Time Stratification in Locally Recurrent Head and Neck Carcinoma after Definitive Radiotherapy or Chemoradiotherapy." Eur Arch Otorhinolaryngol 279.6 (2022) | no info about re-irradiation technique, surgery significant factor but no 1-year and 2-year OS stated |
| Gosh Laskar | "Re-Irradiation in Head and Neck Cancers: Results of Single Institution Prospective Cohort Study." International Journal of Radiation Oncology Biology Physics 105.1 (2019) | Results from patient with adjuvant and definitive radiotherapy were not reported separately, hence no dIMRT data could be extracted |
| Gosh Laskar | "Patient Selection and Outcomes in Reirradiation for Head and Neck Cancers: A Prospective Cohort Study" Clinical oncology (2025) | no info how many patients had nasopharyngeal cancer, although surgery was found to be significant on analysis, no endpoint of interest was stated |
| Granado | "Head and Neck Re-Irradiation: Retrospective Single Institution Study of 60 Patients Treated with Curative Intention." Radiotherapy and Oncology 192 (2024) | no info on tumor localisation, Results from patient with adjuvant and definitive radiotherapy were not reported separately, hence no comparison between aIMRT and dIMRT could be made |
| Granado | "Reirradiation in Head and Neck Cancer. Who Would Benefit? Retrospective Analysis of Our Experience." Radiotherapy and Oncology 182 (2023) | no info on Nasopharyngeal Carcinoma and results from patient with adjuvant and definitive radiotherapy were not reported separately, hence no comparison between aIMRT and dIMRT could be made |
| Guigay | "Adapted Extreme Regimen in the First-Line Treatment of Fit, Older Patients with Recurrent or Metastatic Head and Neck Squamous Cell Carcinoma (Elan-Fit): A Multicentre, Single-Arm, Phase 2 Trial." Lancet Healthy Longev 5.6 (2024) | no radiotherapy, no surgery for recurrence |
| Gulati | "Durvalumab Plus Cetuximab in Patients with Recurrent or Metastatic Head and Neck Squamous Cell Carcinoma: An Open-Label, Nonrandomized, Phase Ii Clinical Trial." Clinical Cancer Research 29.10 (2023) | no radiotherapy, no surgery for recurrence |
| Gupta | "Feasibility of Reirradiation and Treatment Outcome in a Previously Irradiated Territory in Head-and-Neck Malignancies." Egyptian Journal of Otolaryngology 34.4 (2018) | 41,7% adjuvant IMRT but results from patient with adjuvant and definitive radiotherapy were not reported separately, hence no comparison between aIMRT and dIMRT could be made |
| Gutierrez Bayard | "Reirradiation in head and neck recurrent cancer or second primary. Our experience" Oral Oncology 118 (2021) | only 48% IMRT re-irradiation (at least 70% required) |
| Gutierrez Bayard | "Mandibular Osteoradionecrosis (Orn) after Curative Reirradiation in Head and Neck Cancer." Radiotherapy and Oncology 127 (2018) | only comparing Osteoradionecrosis between IMRT and 3d-Conformal Radiotherapy |
| Gutierrez Bayard | "Chronic Radiation-Associated Dysphagia (Rad) after Curative Reirradiation in Head and Neck Cancer." Radiotherapy and Oncology 123 (2017) | only assessing Dysphagia, unclear how many patients treated with IMRT, Results from patient with adjuvant and definitive radiotherapy were not reported separately, hence no comparison between aIMRT and dIMRT could be made |
| Gutierrez Bayard | "Reirradiation in Head and Neck Tumors." European Journal of Cancer 47 (2011) | only used 3D Conformal radiotherapy |
| Gutierrez Bayard | "Evaluation of the use of reirradiation in head and neck recurrent cancer or second primary tumor" Radiotherapy and Oncology 161 (2021) | 48,2% IMRT-re-irradiation (at least 70% required), histology unclear |
| Hanna | "Updated Dose Expansion Results of a Phase 1/1b Study of the Bifunctional Egfr/Tgfβ Inhibitor Bca101 with Pembrolizumab in Patients with Recurrent/Metastatic Head and Neck Squamous Cell Carcinoma." International Journal of Radiation Oncology Biology Physics 118.5 (2024) | no radiotherapy used |
| Harrington | "Lenvatinib ± Pembrolizumab Versus Chemotherapy for Recurrent/Metastatic (R/M) Head and Neck Squamous Cell Carcinoma (Hnscc) That Progressed after Platinum and Immunotherapy: The Phase 2 Leap-009 Study." International journal of radiation oncology biology physics 118.5 (2024) | no radiotherapy used |
| Harrington | "Pembrolizumab (Pembro) for Recurrent Head and Neck Squamous Cell Carcinoma (Hnscc): Post Hoc Analyses of Phase 3 Keynote-040 Prior Radiation Treatment (Rt) and Disease State." Journal of clinical oncology 37 (2019) | no radiotherapy used |
| He | "Dosimetric Evaluation of Sbrt and Imrt for Reirradiation of Recurring Head and Neck Cancer." International Journal of Radiation Oncology Biology Physics 108.3 (2020) | Dosimetry comparison IMRT vs SBRT only, no comparison aIMRT and dIMRT |
| Hecht | "Cetuximab in Combination with Platinum-Based Chemotherapy or Radiotherapy in Recurent and/or Metastatic Scchn in a Non-Selected Patient Cohort (Interimanalysis of the Phase Iv Soccer Trial)." Annals of oncology 27.no pagination (2016) | only used 3D-Conformal radiotherapy |
| Hecht | "A Prospective Real-World Multi-Center Study to Evaluate Progression-Free and Overall Survival of Radiotherapy with Cetuximab and Platinum-Based Chemotherapy with Cetuximab in Locally Recurrent Head and Neck Cancer." Cancers (Basel) 13.14 (2021) | only used 3D-Conformal radiotherapy |
| Hehr | "Docetaxel/Cisplatin Alternating with Re-Irradiation in Recurrent Head and Neck Cancer." Journal of Clinical Oncology 23.16 (2005) | unclear, if IMRT was used (likely only using 3D-Conformal) |
| Hehr | "Reirradiation Alternating with Docetaxel and Cisplatin in Inoperable Recurrence of Head-and-Neck Cancer: A Prospective Phase I/Ii Trial." Int J Radiat Oncol Biol Phys 61.5 (2005) | only used 3D-Conformal radiotherapy |
| Ho | "Impact of Flap Reconstruction on Radiotoxicity after Salvage Surgery and Reirradiation for Recurrent Head and Neck Cancer." Ann Surg Oncol 23.Suppl 5 (2016) | all flap reconstruction before radiotherapy, no comparison post-operative vs. definitive treatment |
| Hoebers | "Reirradiation for Head-and-Neck Cancer: Delicate Balance between Effectiveness and Toxicity." Int J Radiat Oncol Biol Phys 81.3 (2011) | 47% adjuvant radiotherapy, max 52% IMRT used (70% required) |
| Hoell | "Efficacy of Multimodality Approach in Patients with Recurrent Head and Neck Squamous Cell Carcinoma." Anticancer Res 43.3 (2023) | maximum 89% overlapping fields (90% required) |
| Holm | "Dose Planning Study of Proton Versus Photon Radiotherapy for Head and Neck Squamous Cell Carcinoma of Unknown Primary in the Primary and Recurrent Setting." Acta Oncol 62.11 (2023) | Dose planning study, no outcome of interest stated |
| Hong | "Predictive Factors for Locoregional Control after Salvage Reirradiation of Recurrent Head-and-Neck Cancer." International Journal of Radiation Oncology Biology Physics 84.3 (2012) | 43% surgery, IMRT vs other methods, no comparison aIMRT vs dIMRT possible |
| Honig | "The Patterns of Failure after Re-Irradiation (Re-Rt) of Head and Neck Cancer (Hnc) and Their Implications for Defining the Targets." International Journal of Radiation Oncology Biology Physics 72.1 (2008) | unknown, If IMRT was used, Results from patient with adjuvant and definitive radiotherapy were not reported separately, hence no comparison between aIMRT and dIMRT could be made |
| Horichi | "Platinum Resistance and Sensitivity in Recurrent/Metastatic Head and Neck Squamous Cell Carcinoma." Auris Nasus Larynx 51.1 (2024) | unclear, if IMRT was used, unclear how many patients received re-irradiation |
| Horwitz | "Phase Ii Study of Paclitaxel and Cisplatin in Combination with Split Course Concomitant Hyperfractionated Re-Irradiation in Patients with Recurrent Squamous Cell Cancer of the Head and Neck: Results ofradiotherapyog 99-11." International Journal of Radiation Oncology Biology Physics 63.2 (2005) | no IMRT used |
| Hughes | "Quad-Shot-Immunotherapy: Quad-Shot Radiotherapy with Pembrolizumab for Advanced/Recurrent Head and Neck Cancer." Future Oncol 19.22 (2023) | Study protocol only, no patient endpoint stated |
| Iseli | "Postoperative Reirradiation for Mucosal Head and Neck Squamous Cell Carcinomas." Arch Otolaryngol Head Neck Surg 135.11 (2009) | unclear, how many patients received IMRT but unlikely to be above the required 70% |
| Janot | "Randomized Trial of Postoperative Reirradiation Combined with Chemotherapy after Salvage Surgery Compared with Salvage Surgery Alone in Head and Neck Carcinoma." J Clin Oncol 26.34 (2008) | 100% adjuvant radiotherapy, no comparison to dIMRT possible |
| Janssen | "Re-Irradiation of Head-Neck-Tumor - Effect of the Total Dose and Its Further Course of Disease." Strahlentherapie Und Onkologie 186 (2010) | only used 3D-Conformal radiotherapy |
| Janssen | "Re-Irradiation of Head and Neck Cancer-Impact of Total Dose on Outcome." Anticancer Res 30.9 (2010) | only used 3D-Conformal radiotherapy |
| Jeong | "Re-Irradiation of Unresectable Recurrent Head and Neck Cancer: Using Helical Tomotherapy as Image-Guided Intensity-Modulated Radiotherapy." Radiat Oncol J 31.4 (2013). | only 9 patients included (10 required at least) |
| Jereczek-Fossal | "Re-Irradiation: Analysis of Consecutive Patients." Ejc Supplements 3.2 (2005) | only 17% head and neck cancer included |
| Johnny | "Swallowing Outcomes in Re Radiation for Second Primary and Recurrent Head Neck Cancers." Radiotherapy and Oncology 152 (2020) | no outcome of interest stated, unclear if IMRT was used |
| Jones | "Re-Irradiation of the Head and Neck Using Highly Conformal Tomotherapy Intensity-Modulated Radiation Therapy." Minn Med 97.5 (2014) | only 3 patients received definitive IMRT (at least 10 required) |
| Joseph | "Reirradiation after Radical Radiation Therapy: A Survey of Patterns of Practice among Canadian Radiation Oncologists." International Journal of Radiation Oncology Biology Physics 72.5 (2008) | survey of re-RT pattern, no endpoint of interest stated |
| Joshi | "Reirradiation in Head-and-Neck Cancers: An Indian Tertiary Cancer Center Experience." International Journal of Radiation Oncology Biology Physics 84.3 (2012) | only used 3D-Conformal radiotherapy (some in combination with IMRT) |
| Kakria | "Retrospective Analysis of Treatment Outcomes Following Reirradiation in Locoregionally Recurrent Head and Neck Cancer Patients: A Single Institutional Study." Asia Pac J Clin Oncol 11.2 (2015) | 55% IMRT (at least 70% required), unclear, how many received adjuvant radiotherapy (max 23%) |
| Kanakamedala | "Re-Irradiation of Head-and-Neck Cancers: Volume Re-Irradiated Predicts for Long-Term Complications." International Journal of Radiation Oncology Biology Physics 87.2 (2013) | no re-irradiation applied |
| Kao | "Phase 1 Trial of Concurrent Erlotinib, Celecoxib and Reirradiation for Recurrent Head and Neck Cancer." Cancer 117.14 (2011) | 43% adjuvant IMRT |
| Kao | "Phase I Trial of Concurrent Erlotinib, Celecoxib and Reirradiation for Recurrent Head and Neck Cancer." Journal of Clinical Oncology 28.15 (2010) | 6 patients received adjuvant IMRT, 8 patients received definitive IMRT (at least 10 required) |
| Kasperts | "Results of Postoperative Reirradiation for Recurrent or Second Primary Head and Neck Carcinoma." Cancer 106.7 (2006) | 100% postoperative radiotherapy, no comparison to dIMRT possible |
| Katodritis | "Chemo re-irradiation in recurrent head and neck cancer: a single institution experience" J buon 17.2 (2012) | unclear, if IMRT was used, only reports 15 patients, 4 had surgery (at least 10 required) |
| Kaur | "Role of Salvage Surgery in Recurrent and Second Primary Head and Neck Squamous Cell Carcinoma" Indian Journal of Surgical Oncology (2025) | at maximum 7/40 patients re-irradiated, thus patient number too low |
| Kazemian | "Outcomes of definitive radiotherapy vs. laryngectomy followed by adjuvant radiotherapy in patients with locally advanced laryngeal squamous cell carcinoma: real-world experience in a referral cancer center" Radiation Oncology (2024) | no recurrent cancers included |
| Kharofa | "Continuous-Course Reirradiation with Concurrent Carboplatin and Paclitaxel for Locally Recurrent Nonmetastatic Squamous Cell Carcinoma of the Head-and-Neck." Int J Radiat Oncol Biol Phys 83.2 (2012) | only states median OS for aIMRT and dIMRT, no info on 1- and 2-year OS |
| Kil | "Pharyngeal Constrictor-Sparing Salvage Stereotactic Body Radiation Therapy With Tongue-Out for In-Field Recurrence After Definitive Radiation Therapy for Head and Neck Cancer: Guide to Tongue-Out Radiation Therapy" Practical Radiation Oncology (2025) | SBRT review |
| Kodama | "Paclitaxel plus cetuximab versus nivolumab for patients with platinum-refractory recurrent or metastatic head and neck squamous cell carcinoma: a retrospective analysis" International Journal of Clinical Oncology (2025) | no re-irradiation applied |
| Kornek | "Phase Ii Study of Capecitabine (X) Plus Reirradiation in Patients (Pts) with Recurrent Squamous Cell Carcinoma of the Head and Neck (Scchn)." Ejc Supplements 3.2 (2005) | Cancer localisation unclear, only included unresected cancer, no comparison to adjuvant treatment possible |
| Koukourakis | "Anti-PD-1 immunotherapy with dose-adjusted ultra-hypofractionated re-irradiation in patients with locoregionally recurrent head and neck cancer" Clin Transl Oncol 25.10 (2023) | only definitive SBRT |
| Kramer | "Toxicity and Outcome Analysis of Patients with Recurrent Head and Neck Cancer Treated with Hyperfractionated Split-Course Reirradiation and Concurrent Cisplatin and Paclitaxel Chemotherapy from Two Prospective Phase I and Ii Studies." Head Neck 27.5 (2005) | no IMRT used |
| Krstevska | "Prognostic Factors in Patients with Recurrent Head and Neck Cancer Treated with Reirradiation." J buon 13.3 (2008) | no IMRT used |
| Kumar | "The Feasibility of Comprehensive Volume Hyperfractionated (Hfx) Re-Irradiation (Re-Rt) for Recurrent Head and Neck (H/N) Cancer." Journal of Clinical Oncology 28.15 (2010) | only 3D conformal-radiotherapy used |
| Langendijk | "A Phase Ii Study of Primary Reirradiation in Squamous Cell Carcinoma of Head and Neck." Phase Ii Study of Primary Reirradiation in Squamous Cell Carcinoma of Head and Neck (2006) | no IMRT used |
| Langer | "Phase Ii Study of Low-Dose Paclitaxel and Cisplatin in Combination with Split-Course Concomitant Twice-Daily Reirradiation (Xrt) in Recurrent Squamous Cell Carcinoma of the Head and Neck (Scchn): Long-Term Follow-up of Radiation Therapy Oncology Group (Rtog) Protocol 9911." Journal of Clinical Oncology 30.15 (2012) | unclear if IMRT used |
| Langer | "Phase Ii Study of Low-Dose Paclitaxel and Cisplatin in Combination with Split-Course Concomitant Twice-Daily Reirradiation in Recurrent Squamous Cell Carcinoma of the Head and Neck: Results of Radiation Therapy Oncology Group Protocol 9911." J Clin Oncol 25.30 (2007) | no IMRT used |
| Lee | "Outcomes of Reirradiation in Head and Neck Cancers: Two Institutional Experience." Radiotherapy and Oncology 152 (2020) | 25% nasopharyngeal cancer (maximum 20% allowed) |
| Lee | "Re-Irradiation for Recurrent or Second Primary Head and Neck Cancer." Radiat Oncol J 39.4 (2021) | 35,7 nasopharyngeal cancer (maximum 20% allowed) |
| Lee | "Intensity-Modulated Radiotherapy-Based Reirradiation for Head and Neck Cancer: A Multi-Institutional Study by Korean Radiation Oncology Group (Krog 1707)." Cancer Res Treat 52.4 (2020) | 25% Nasopharynx carcinoma (maximum 20% allowed) |
| Lee | "Shorter Reirradiation Intervals for Head and Neck Cancer Are Associated With Severe Long-Term Toxicity" International Journal of Radiation Oncology Biology Physics 94.4 (2016) | Results from patient with adjuvant and definitive radiotherapy were not reported separately, hence no comparison between aIMRT and dIMRT could be made but stated that long toxicity was insignificant |
| Lee | "Predictors of Severe Long-Term Toxicity after Re-Irradiation for Head and Neck Cancer." Oral Oncol 60 (2016) | Results from patient with adjuvant and definitive radiotherapy were not reported separately, hence no comparison between aIMRT and dIMRT could be made but stated that long toxicity was insignificant and surgery improved loco-regional control |
| Lee | "Salvage Re-Irradiation for Recurrent Head and Neck Cancer." Int J Radiat Oncol Biol Phys 68.3 (2007) | eligible but overlap to Ward et al. 2018 as the same institution contributed to the MIRI collaborative and the inclusion periods overlap |
| Leeman | "Patterns of Treatment Failure and Postrecurrence Outcomes among Patients with Locally Advanced Head and Neck Squamous Cell Carcinoma after Chemoradiotherapy Using Modern Radiation Techniques." JAMA Oncol 3.11 (2017) | unclear if IMRT used, % of adjuvant radiotherapy is also unknown, Results from patient with adjuvant and definitive radiotherapy were not reported separately, hence no comparison between aIMRT and dIMRT could be made |
| León | "Prognostic Significance of Extracapsular Spread in Isolated Neck Recurrences in Head and Neck Squamous Cell Carcinoma Patients." Eur Arch Otorhinolaryngol 274.1 (2017) | unclear if IMRT was used, all patients had neck dissection so no comparison to definitive IMRT is possible |
| Li | "Initial Analysis of the Synergy of Programmed Cell Death-1 (Pd-1) Inhibitor and Concurrent Chemoradiotherapy Treatment for Recurrent/Metastatic Head and Neck Squamous Cell Carcinoma Patients." Radiat Oncol 18.1 (2023) | 60% recurrence, 40% distant metastasis, so not enough overlapping fields (at least 90% required), for recurrences only 1 of 24 patients received surgery so no comparison aIMRT to dIMRT possible, as at least 10 post-operative cases needed |
| Lin | "HyperArcTM volumetric modulated arc therapy for hypopharyngeal cancer with solitary recurrence in the cervical vertebra: A case report and literature review" Medicine (Baltimore) 103.23 (2024) | case report only (at least 10 patients required in each arm) |
| Lindegaard | "Outcome in Patients with Isolated Regional Recurrence after Primary Radiotherapy for Head and Neck Cancer." Head Neck 42.11 (2020) | only 2 patients received definitive IMRT, 82 received surgery, so no comparison was possible as at least 10 patients should have definitive IMRT |
| Lopater | "Re-Irradiation of Head-and-Neck Cancer with Highly Conformal Tomotherapy Imrt - Management of Recurrent Head-and-Neck Squamous Cell Carcinoma." International Journal of Radiation Oncology Biology Physics 88.2 (2014) | only 3 patients received definitive IMRT, 21 received surgery, so no comparison was possible as at least 10 patients should have definitive IMRT |
| Lucas | "Phase 1/2 Clinical Trial of Re-Irradiation with Pemetrexed and Erlotinib Followed by Maintenance Erlotinib for Recurrent and Second Primary Squamous Cell Carcinoma of the Head and Neck (Scchn)." International Journal of Radiation Oncology Biology Physics 90 (2014) | No info on tumor localisation, only including unresected patients, no comparison to aIMRT possible, NCT00573989 |
| Ma | "High Recurrence for Hpv-Positive Oropharyngeal Cancer with Neoadjuvant Radiation Therapy to Gross Disease Plus Immunotherapy: Analysis from a Prospective Phase Ib/Ii Clinical Trial." Int J Radiat Oncol Biol Phys 117.2 (2023) | only primary tumor treatment, no re-irradiation applied |
| Machtay | "Postoperative Radiotherapy ± Cetuximab for Intermediate-Risk Head and Neck Cancer" Journal of clinical oncology (2025) | only initial diagnosed cancer, no re-irradiation applied, Results from patient with adjuvant and definitive radiotherapy were not reported separately, hence no comparison between aIMRT and dIMRT could be made |
| Mallick | "Re-Irradiation in Head and Neck Cancers: An Indian Tertiary Cancer Centre Experience." J Laryngol Otol 128.11 (2014) | only 9% of the included patients received IMRT re-irradiation (at least 70% required) |
| Malukar | "Re-Irradiation in Head and Neck Cancers: Real-World Experience from a Tertiary Cancer Institute." Radiotherapy and Oncology 192 (2024) | No info on histology, tumor localisation |
| Margalit | "Patterns of Failure after Reirradiation (Rert) with Intensity Modulated Radiation Therapy (Imrt): Not Just a Local Issue." International Journal of Radiation Oncology Biology Physics 93.3 (2015) | only describes pattern of failure, no endpoint of interest stated for aIMRT and dIMRT, separately |
| Margalit | "Toxicity and Survival after Reirradiation (Rert) with Imrt for Recurrent Squamous Cell Cancer of the Head and Neck (Scchn)." International Journal of Radiation Oncology Biology Physics 88.2 (2014) | Results from patient with adjuvant and definitive radiotherapy were not reported separately, hence no comparison between aIMRT and dIMRT could be made, found that aIMRT had significantly lower toxicity rates but no absolute number given |
| Margalit | "Patterns of Failure after Reirradiation with Intensity-Modulated Radiation Therapy and the Competing Risk of out-of-Field Recurrences." Oral Oncol 61 (2016) | describes pattern of failure between aIMRT and dIMRT but no 1-year and 2-year LRC was stated for these groups |
| Margalit | "Patient-Oriented Toxicity Endpoints after Head and Neck Reirradiation with Intensity Modulated Radiation Therapy." Oral Oncol 73 (2017) | 45% adjuvant IMRT, radiotherapy not clearly defined, surgery was no significant prognostic factor, although no absolute number given |
| Martin | "Patterns of Failure for Recurrent Head and Neck Squamous Cell Carcinoma Treated with Salvage Surgery and Reirradiation Using Imrt." International Journal of Radiation Oncology Biology Physics 93.3 (2015) | 100% adjuvant IMRT, no comparison to dIMRT possible |
| Martin | "Patterns of Failure for Recurrent Head and Neck Squamous Cell Carcinoma Treated With Salvage Surgery and Reirradiation using IMRT" International Journal of Radiation Oncology Biology Physics 94.4 (2016) | 100% adjuvant IMRT, no comparison to dIMRT possible |
| Martin | "Reirradiation with Concomitant Chemotherapy in Platinum Refractory Head and Neck Cancers." Radiotherapy and Oncology 82 (2007) | only used 3D-Conformal radiotherapy |
| Martinez-Trufero | "Sequential Therapy with Induction Chemotherapy and Chemoradiotherapy in Pre-Irradiated Recurrent Head and Neck Squamous Cell Carcinoma Incorporating Nab-Paclitaxel—a Commentary of the Phase I Trial of Afhx Followed by Concomitant Fhx Scheme." Translational Cancer Research 12.4 (2023) | Commentary, not providing original patient data |
| Maruo | "Comparison of Salvage Surgery for Recurrent or Residual Head and Neck Squamous Cell Carcinoma." Japanese Journal of Clinical Oncology 50.3 (2020): 288-95. Print. | Salvage surgery alone vs. other treatment, no info about re-irradiation technique, no comparison aIMRT to dIMRt possible |
| Marwaha | "Clinical Efficacy and Tolerability of Continuous Course Reirradiation with Concurrent Weekly Carboplatin-Paclitaxel for Locally Recurrent, Nonmetastatic Squamous Cell Carcinoma of the Head and Neck (Scchn)." Journal of Clinical Oncology 28.15 (2010) | No info about method re-irradiation |
| Massa | "Phase II Study of Vinorelbine/Cetuximab in Patients with Recurrent/Metastatic Squamous Cell Carcinoma of the Head and Neck Progressing after at Least Two Chemotherapy Regimens." Oral Oncol 46.11 (2010) | 17% of patients received radiation for primary cancer, so at maximum 83% overlapping fields (90% required) |
| Matsuo | "Interval to Recurrence Affects Survival in Recurrent Head and Neck Squamous Cell Carcinoma." Cancer Diagn Progn 4.5 (2024) | no info on salvage treatment for recurrence |
| Megahed | "Re-irradiation of recurrent head and neck cancers using pulsed reduced dose rate radiotherapy: An institutional series" Oral Oncology (2024) | only 9 patients, 8 received adjuvant IMRT, at least 10 required for each arm |
| Mehta | "Optimising Reirradiation Strategies in Head and Neck Cancers: Assessing Acute Adverse Events Through Overlapping Treatment Plan Volumes"Clinical Oncology 36.9 (2024) | no info on re-irradiation method used, no info on tumor location, histology and rate of salvage surgery |
| Mendenhall | "Re-Irradiation of Head and Neck Carcinoma." Am J Clin Oncol 31.4 (2008) | review only, no original patient data presented |
| Merlano | "Fast Tumor Re-Growth of Head and Neck Cancer (Hnc) after Compassionate Treatment (Ctr) with Re-Irradiation, Carboplatin and Concurrent Cetuximab." Annals of Oncology 16 (2005) | 4 patients included (at least 10 required) |
| Mesko | "Head and Neck Reirradiation with Proton Therapy (Pbt), Imrt or Stereotactic Radiotherapy (Sabr): Clinical Outcomes of a Prospective Registry." International Journal of Radiation Oncology Biology Physics 105.1 (2019) | no info on tumor localisation, compares IMRT to SBRT and proton but not aIMRT to dIMRT |
| Micera | "Re-Irradiation for Recurrence and Second Primary Head and Neck Cancer: A Single Center Experience." Radiotherapy and Oncology 111 (2014) | no info on histology and tumor localisation, Results from patient with adjuvant and definitive radiotherapy were not reported separately, hence no comparison between aIMRT and dIMRT could be made |
| Mikhailov | "Reirradiation in Patients with Locoregional Recurrence of Head and Neck Cancer - Single Institution Experience." International Journal of Radiation Oncology Biology Physics 106.5 (2020) | 30 % SBRT was used, only definitive IMRT and no info on tumor localisation and only stated toxicities, no comparison to aIMRT possible |
| Mikhailov | "Reirradiation with Simultaneously Integrated Boost (Sib) in Patients with Local Recurrence of Squamous Cell Carcinoma of the Head and Neck: Own Experience." International Journal of Radiation Oncology Biology Physics 94.4 (2016) | no info on tumor sites, no info, how many received surgery before reirradiation, only toxicities stated, Results from patient with adjuvant and definitive radiotherapy were not reported separately, hence no comparison between aIMRT and dIMRT could be made |
| Mikhaylov | "Chemo-Reirradiation with Simultaneously Integrated Boost in Patients with Local Recurrence of Hnscc." Radiotherapy and Oncology 123 (2017) | no info on tumor sites, no info, how many received surgery before reirradiation, Results from patient with adjuvant and definitive radiotherapy were not reported separately, hence no comparison between aIMRT and dIMRT could be made |
| Milano | "Twice-Daily Reirradiation for Recurrent and Second Primary Head-and-Neck Cancer with Gemcitabine, Paclitaxel and 5-Fluorouracil Chemotherapy." Int J Radiat Oncol Biol Phys 61.4 (2005) | no info on re-irradiation technique, compares 5-year OS between measurable disease and non-measurable, but not all surgery resulted in R0 margin, thus Results from patient with adjuvant and definitive radiotherapy were not reported separately, hence no comparison between aIMRT and dIMRT could be made |
| Milanovic | "Re-Irradiation and Erbitux by Patients with Recurrent Head and Neck Cancer." Radiotherapy and Oncology 96 (2010) | only includes in-operable patients, no info if IMRT was used, hence no comparison to aIMRT possible |
| Milanovic | „Reirradiation plus EGFR inhibition in locally recurrent and unresectable head and neck cancer: final results from a single institution” Strahlenther Onkol (2013) | Only dIMRT, no comparison to aIMRT possible |
| Milanovic | "Reirradiation and Cetuximab in Patients with Locally Recurrent and Unresectable Head and Neck Cancer - Is there any Impact on Survival? - Final Results from Single Institution and Literature Review" Oncology Research and Treatment 37 (2014) | only includes in-operable patients, no info if IMRT was used, no comparison to aIMRT possible |
| Milanovic | "Reirradiation, cetuximab and itraconazole in locally recurrent and unresectable head and neck cancer" Radiotherapy and Oncology (2015) | only 5 patients included (at least 10 per arm required) |
| Mireștean | "Immunotherapy with Pd-1 Inhibitor Nivolumab in Recurrent/Metastatic Platinum Refractory Head and Neck Cancers-Early Experiences from Romania and Literature Review." Diagnostics (Basel) 13.16 (2023) | only 11% had re-irradiation (at least 90% required) |
| Mohamad | "Re-Irradiation for Recurrent Head and Neck Cancer: Freedom from Cancer Recurrence Rate." J Clin Med 12.8 (2023) | 25% nasopharyngeal cancer (maximum 20% allowed) |
| Mohamed | "Patterns of Failure for Recurrent Head and Neck Squamous Cell Carcinoma Treated with Salvage Surgery and Postoperative Imrt Reirradiation." Clin Transl Radiat Oncol 44 (2024) | 100% had adjuvant IMRT, no comparison to dIMRT possible |
| Moreno | "Reirradiation with Dynamic Adaptative Radiation Therapy (Dart) and Image Guided Technique (Igrt) in Head and Neck Tumors." Radiotherapy and Oncology 98 (2011) | no info how many pat. received salvage surgery, Results from patient with adjuvant and definitive radiotherapy were not reported separately, hence no comparison between aIMRT and dIMRT could be made |
| Morris | "Phase I Study of Proteasome Inhibitor Bortezomib (B) Concurrent with Re-Irradiation Therapy (Re-Rt) for Recurrent Squamous Cell Carcinoma of the Head and Neck (Scchn)." Journal of Clinical Oncology 28.15 (2010) | no info on re-irradiation technique, no endpoint of interest stated (only median OS , median PFS), Results from patient with adjuvant and definitive radiotherapy were not reported separately, hence no comparison between aIMRT and dIMRT could be made |
| Nathalie | "Prospective study of concomitant stereotaxic reirradiation and mitomycin with cetuximab for recurrent head and neck cancer" | only including unresected cancer, treated with SBRT |
| Nedzi | "Toxicity of Continuous Course Re-Irradiation Concurrent with Weekly Cisplatinum and Cetuximab for Recurrent Squamous Cell Carcinoma of the Head and Neck: Report of a Phase 2 Trial." International Journal of Radiation Oncology Biology Physics 87.2 (2013) | only states toxicity but Results from patient with adjuvant and definitive radiotherapy were not reported separately, hence no comparison between aIMRT and dIMRT could be made |
| Ng | "Patterns of Failure after Definitive Reirradiation for Patients with Head and Neck Cancer." International Journal of Radiation Oncology Biology Physics 105.1 (2019) | only includes definitive IMRT, no comparison to aIMRT possible |
| Ng | "Patterns of Failure after Imrt and Proton Re-Irradiation for Patients with Recurrent Head and Neck Cancer." International Journal of Radiation Oncology Biology Physics 111.3 (2021) | only includes definitive IMRT, no comparison to aIMRT possible, only described pattern of failure |
| Nguyen | "Patient-Reported Quality of Life Immediately After Head and Neck Reirradiation With Highly Conformal Radiation Therapy" International Journal of Radiation Oncology Biology Physics 99.2 (2017) | compares QoL outcomes between SBRT and IMRT, no endpoint of interest stated, Results from patient with adjuvant and definitive radiotherapy were not reported separately, hence no comparison between aIMRT and dIMRT could be made |
| Noronha | "Long-Term Update of a Phase 3 Randomized Study Comparing Once-a-Week Versus Once-Every-3-Weeks Cisplatin Along With Radiation in Head and Neck Cancer" Int J Radiat Oncol Biol Phys (2024) | only initial diagnosis / primary cancers included, no re-irradiation applied |
| Obermeier | "Lymph Nodal Recurrence in Levels Iv and V in Oral Squamous Cell Carcinoma after Neck Dissection." ANZ J Surg 93.6 (2023) | analysis of recurrence pattern, no endpoint of interest stated, As no separate outcome values for aIMRT and dIMRT-cohort were stated/ provided, no comparison between outcomes of dIMRT and aIMRT made |
| Ohnleiter | "Factors Improving the Outcome of Patients Re-Irradiated with Intensity-Modulated Radiotherapy (Imrt) for Relapse or New Head and Neck Cancer Developed in Irradiated Areas." Chin Clin Oncol 7.6 (2018) | surgery was significant factor for PFS and LRC but no 1-year or 2-year values stated |
| Ooishi | "Patterns of Failure after Postoperative Intensity-Modulated Radiotherapy for Locally Advanced and Recurrent Head and Neck Cancer." Jpn J Clin Oncol 46.10 (2016) | outcome of primary therapy provided, no statement about recurrence treatment |
| Orlandi | "Long-Term Outcome of Re-Irradiation for Recurrent or Second Primary Head and Neck Cancer: A Multi-Institutional Study of Airo-Head and Neck Working Group." Head Neck 41.10 (2019) | 30% Nasopharyngeal cancer (max 20%), Results from patient with adjuvant and definitive radiotherapy were not reported separately, hence no comparison between aIMRT and dIMRT could be made |
| Parashar | "Importance of Contouring the Cervical Spine Levels in Imrt Radiation for Head and Neck Cancers: Implications for Re-Irradiation." Radiotherapy and Oncology 88 (2008) | dosimetry study only, no endpoint of interest stated |
| Park | "Outcome of Cetuximab in Recurrent/Metastatic Head and Neck Squamous Cell Carcinoma (R/M Hnscc) Post-Immune Checkpoint Inhibitor (Ici) Treatment." International Journal of Radiation Oncology Biology Physics 118.5 (2024) | no radiotherapy applied |
| Patel | "High Dose Re-Irradiation Using 3D-Crt and Imrt for Recurrent and Second Primary Head and Neck Cancers." European Journal of Cancer 49 (2013) | no endpoint of interest stated, no info about tumor localisation, Results from patient with adjuvant and definitive radiotherapy were not reported separately, hence no comparison between aIMRT and dIMRT could be made |
| Peponi | "Reirradiation for Recurrent Head and Neck Carcinoma." J buon 17.3 (2012) | no info on re-irradiation technique used, only 6 patients received post-operative radiation (at least 10 required) |
| Phan | "Reirradiation with SBRT, IMRT and Proton Therapy for Recurrent Oropharynx Squamous Cell Carcinoma: Efficacy and Toxicity Outcomes" International Journal of Radiation Oncology Biology Physics 106.5 (2020) | Results from patient with adjuvant and definitive radiotherapy were not reported separately, hence no comparison between aIMRT and dIMRT could be made, no info if surgery was performed |
| Phan | "Reirradiation for Locally Recurrent Head and Neck Cancer: State-of-the-Art and Future Directions" Seminars in Radiation Oncology (2025) | review only, no original patient data presented |
| Phuong | "Challenges in the Re-Irradiation of Locally Advanced Head and Neck Cancers: Outcomes and Toxicity from a Single Institution." International Journal of Radiation Oncology Biology Physics 101.2 (2018) | only 7 patients received definitive re-irradiation (at least 10 required), no difference in toxicity or survival found |
| Phuong | "Challenges in the Re-Irradiation of Locally Advanced Head and Neck Cancers: Outcomes and Toxicities." Journal of Radiation Oncology 8.3 (2019) | only 8 patients received definitive re-irradiation (at least 10 required), no difference in toxicity or survival found |
| Platteaux | "Outcome after Re-Irradiation of Head and Neck Cancer Patients." Strahlenther Onkol 187.1 (2011) | only 19% of patients received IMRT-based re-irradiation, (at least 70% required) |
| Pollard | "Prospective Analysis of Patient Reported Symptom Burden After Head and Neck Reirradiation With Intensity Modulated and Stereotactic Body Radiation Therapy" International Journal of Radiation Oncology Biology Physics 99.2 (2017) | compares QoL between IMRT and SBRT, no endpoint of interest stated, Results from patient with adjuvant and definitive radiotherapy were not reported separately, hence no comparison between aIMRT and dIMRT could be made |
| Pollard | "Clinical outcomes after local field conformal reirradiation of patients with retropharyngeal nodal metastasis" Head Neck 39.10 (2017) | only 7 patients received IMRT (at least 10 required for each arm) |
| Popovtzer | "The Pattern of Failure after Reirradiation of Recurrent Squamous Cell Head and Neck Cancer: Implications for Defining the Targets." Int J Radiat Oncol Biol Phys 74.5 (2009) | unclear how many patients received IMRT |
| Porosnicu | "Phase 1 Clinical Trial of Re-Irradiation with Pemetrexed and Erlotinib Followed by Maintenance Erlotinib for Patients with Recurrent and Second Primary Squamous Cell Carcinoma of the Head and Neck- Management of Recurrent Hea-and-Neck Squamous Cell Carcinoma." International Journal of Radiation Oncology Biology Physics 88.2 (2014) | only definitive re-irradiation was used, no comparison to aIMRT was possible |
| Rades | "Re-Irradiation with 36 Gy (1.5 Gy Twice Daily) Plus Paclitaxel for Advanced Recurrent and Previously Irradiated Scchn Is Feasible." Anticancer Res 38.1 (2018) | only 4 patients included, (at least 10 required) |
| Rades | "Hyperfractionated or Accelerated Hyperfractionated Re-Irradiation with ≥42 Gy in Combination with Paclitaxel for Secondary/Recurrent Head-and-Neck Cancer." Anticancer Res 38.6 (2018) | 2 patient case study (at least 10 required) |
| Rades | "A New Scoring-System for Estimating Overall Survival after Radiotherapy of Recurrent Head and Neck Cancers." Anticancer Res 38.3 (2018) | unclear how many patients had IMRT re-irradiation, tumor localisation and how many irradiated in overlapping field not stated, Results from patient with adjuvant and definitive radiotherapy were not reported separately, hence no comparison between aIMRT and dIMRT could be made |
| Rahmati | "Outcomes in Recurrent Head and Neck Cancer after Salvage Surgery and Flap Reconstruction with Postoperative Reirradiation (Re-Rt)." International Journal of Radiation Oncology Biology Physics 81.2 (2011) | 100% adjuvant radiation, no comparison to dIMRT possible |
| Ramasamy | "Clinical Outcomes Following Reirradiation in Head and Neck Cancers in the Intensity Modulated Radiation Therapy Era." International Journal of Radiation Oncology Biology Physics 93.3 (2015) | Results from patient with adjuvant and definitive radiotherapy were not reported separately, hence no comparison between aIMRT and dIMRT could be made, 1 patient had brachytherapy (not allowed co-intervention) |
| Ramprasad | "Quality of Life in Patients with Recurrent and Second Primary Head and Neck Cancer." Otolaryngol Head Neck Surg 168.2 (2023) | 45% of recurrences treated with surgery alone, maximum 55% overlapping fields (90% required) |
| Riaz | "A Nomogram to Predict Loco-Regional Control after Re-Irradiation for Head and Neck Cancer." Radiother Oncol 111.3 (2014) | eligible but overlap to Ward et al. 2018 as the same institution contributed to the MIRI collaborative and the inclusion periods overlap |
| Rivera | "Cetuximab in Metastatic or Recurrent Head and Neck Cancer: The Extreme Trial." Expert Rev Anticancer Ther 9.10 (2009) | no IMRT re-irradiation was used |
| Roesch | "Re-Irradiation for Patients with Head and Neck Squamous Cell Carcinoma - a Multicenter Analysis of 253 Patients of the Jdegro Trial Group." Strahlentherapie Und Onkologie 197.SUPPL 1 (2021) | definitive IMRT, no comparison to aIMRT possible |
| Roesch | "Re-Irradiation in Head & Neck Cancer - a Pooled Analysis of 253 Individual Cases." Radiotherapy and Oncology 161 (2021) | definitive IMRT, no comparison to aIMRT possible |
| Roesch | "Dose-Escalated Re-Irradiation Improves Outcome in Locally Recurrent Head and Neck Cancer - Results of a Large Multicenter Analysis." Radiother Oncol 181 (2023) | definitive IMRT, no comparison to aIMRT possible |
| Roman | "Re-Irradiation with Concurrent Chemotherapy for Recurrent Head and Neck Cancer." Journal of Clinical Oncology 24.18 (2006) | unclear, how many received adjuvant radiotherapy but only 13 patients included, (at least 10 should be in every arm) |
| Rosenberg | "A Phase I Trial of Nab-Paclitaxel-Based Induction Followed by Nab-Paclitaxel-Based Concurrent Chemotherapy and Re-Irradiation in Previously Treated Head and Neck Squamous Cell Carcinoma." Annals of Oncology 32 (2021) | only included unresected patients, no comparison to aIMRT was possible |
| Rosenberg | "Phase I Study of Nab-Paclitaxel-Based Induction Followed by Nab-Paclitaxel-Based Concurrent Chemotherapy and Re-Irradiation in Previously Treated Head and Neck Squamous Cell Carcinoma." Br J Cancer 127.8 (2022) | 39% adjuvant radiotherapy but Results from patient with adjuvant and definitive radiotherapy were not reported separately, hence no comparison between aIMRT and dIMRT could be made |
| Rudžianskas | "Reirradiation for Patients with Recurrence Head and Neck Squamous Cell Carcinoma: A Single-Institution Comparative Study." Medicina (Kaunas) 50.2 (2014) | 100% 3D Conformal therapy was used |
| Ruehle | "Re-Irradiation of Malignant Head and Neck Tumors: Oncological Results and Toxicities of a Large Monoinstitutional Cohort." Strahlentherapie Und Onkologie 196.SUPPL 1 (2020) | In this abstract Results from patient with adjuvant and definitive radiotherapy were not reported separately, hence no comparison between aIMRT and dIMRT could be made. The later published study was included as it stated the desired outcomes, separately. |
| Rühle | "The Value of Moderate Dose Escalation for Re-Irradiation of Recurrent or Second Primary Head-and-Neck Cancer." Radiat Oncol 15.1 (2020) | included |
| Rusthoven | "Initial Results of a Phase I Dose Escalation Trial of Concurrent and Maintenance Erlotinib and Re-Irradiation for Recurrent and New Primary Head and Neck Cancer." International Journal of Radiation Oncology Biology Physics 75.3 (2009) | unclear what method of re-irradiation was used, unclear how many patients received surgery, As no separate outcome values for aIMRT and dIMRT-cohort were stated/ provided, no comparison between aIMRT an dIMRt was possible |
| Rusthoven | "Initial Results of a Phase I Dose-Escalation Trial of Concurrent and Maintenance Erlotinib and Reirradiation for Recurrent and New Primary Head-and-Neck Cancer." Int J Radiat Oncol Biol Phys 78.4 (2010) | unclear what method of re-irradiation was used, unclear how many patients received surgery, As no separate outcome values for aIMRT and dIMRT-cohort were stated/ provided, no comparison between aIMRT an dIMRt was possible |
| Saba | "Ca209-9ky: Results of a Phase Ii Study of Intensity Modulated Radiotherapy (Imrt) Re-Irradiation and Concurrent/Adjuvant Nivolumab (Nivo) in Patients with Loco-Regionally Recurrent or Second Primary (Rspt) Head and Neck Squamous Cell Carcinoma (Hnscc)." Journal of Clinical Oncology 40.16 (2022) | unclear tumor localisation, % adjuvant IMRT, overlap to included article and Results from patient with adjuvant and definitive radiotherapy were not reported separately, hence no comparison between aIMRT and dIMRT could be made |
| Saba | "Ca209-9ky: Phase Ii Study of Imrt Re-Irradiation and Concurrent/Adjuvant Nivolumab (Nivo) in Patients with Loco Regionally Recurrent or Second Primary Head and Neck Squamous Cell Carcinoma (Hnscc) - Toxicity and Quality of Life (Qol) Results." International Journal of Radiation Oncology Biology Physics 111.3 (2021) | unclear tumor localisation, % adjuvant IMRT, overlap to included article and Results from patient with adjuvant and definitive radiotherapy were not reported separately, hence no comparison between aIMRT and dIMRT could be made |
| Saba | "Intensity-Modulated Reirradiation Therapy With Nivolumab in Recurrent or Second Primary Head and Neck Squamous Cell Carcinoma: A Nonrandomized Controlled Trial" JAMA Oncol (2024) | included |
| Sacco | "Phase 2 Trial of at-101 in Combination with Docetaxel for Recurrent, Locally Advanced or Metastatic Head-and-Neck Squamous Cell Carcinoma (Hnscc) Management of Recurrent Head-and-Neck Squamous Cell Carcinoma." International journal of radiation oncology biology physics 88.2 (2014) | no recurrent cancer included |
| Saint-Ghislain | "Hyperfractionated Reirradiation with Cetuximab for Recurrent Head and Neck Cancer: The Gortec 2008-01 Multicentric Phase Ii Study." Journal of Clinical Oncology 36.15 (2018) | only included unresected patients, no comparison to aIMRT was possible, no info on re-irradiation technique |
| Salama | "Phase I Study of Concomitant Chemoradiotherapy with Irinotecan, 5-FU and Hydroxyurea for Patients with Advanced and/or Recurrent Head and Neck Cancer." Cancer J 11.2 (2005) | 62% overlapping fields (at least 90% required), method of radiotherapy unclear |
| Salama and Vokes | "Concurrent Chemotherapy and Re-Irradiation for Locoregionally Recurrent Head and Neck Cancer." Semin Oncol 35.3 (2008) | Review/Comment, no original patient’s data presented |
| Salama | "Long-Term Outcome of Concurrent Chemotherapy and Reirradiation for Recurrent and Second Primary Head-and-Neck Squamous Cell Carcinoma." Int J Radiat Oncol Biol Phys 64.2 (2006) | no IMRT used |
| Saloura | "IMRRT for Locoregionally Recurrent Head and Neck Squamous Cell Carcinoma" GRANTS:15552651 (2020) | no full text available, only included unresected cancer NCT03803774, no comparison to aIMRT possible |
| Salunkhe | "Re-Irradiation in Head and Neck Cancers: A Single Institution Prospective Cohort Study." Radiotherapy and Oncology 122 (2017) | Compared surgery vs surgery and post-operative re-irradiation, no comparison dIMRT vs. aIMRT was made |
| Sarkar | "A Comparative Dose-Escalation Analysis for Reirradiated Cancer Patients with and without Appropriate Dose Mapping." Radiat Environ Biophys 63.1 (2024) | dosimetry study, no endpoint of interest stated |
| Sarkar | "A Comparative Dose-Escalation Analysis for the Head and Neck Reirradiation Patients with and without Appropriate Dicom Based Dose-Volume Information of Primary Radiotherapy." International Journal of Radiation Oncology Biology Physics 114.3 (2022) | dosimetry study, no endpoint of interest stated |
| Sato | "Induction Chemotherapy with Paclitaxel, Carboplaton and Cetuximab (Pce) Followed by Chemoradiotherapy for Unresectable Locoregional Recurrence after Curative Surgery in Patients with Squamous Cell Carcinoma of the Head and Neck." Frontiers in Oncology 14 (2024) | no prior radiotherapy applied, no overlapping fields irradiated |
| Saunders | "Re-Irradiation Strategies for Loco-Regional Recurrent Unresectable Disease in Squamous Cell Carcinoma of Head and Neck Cancer." Oral Oncology 1.1 (2005) | PET-CT signal study, no endpoint of interest stated |
| Schatteman | " A feasibility study on adaptive 18F-FDG-PET-guided radiotherapy for recurrent and second primary head and neck cancer in the previously irradiated territory “ Strahlentherapie und Onkologie (2018) | only 10 patients included, (at least 10 patients must be in each arm), only definitive IMRT |
| Schleifenbaum | "Oncologic Therapy Results of Head and Neck Tumor Patients with Local Recurrences - Analysis of Therapy Modality Re-Irradiation and Radiation Dose." Strahlentherapie Und Onkologie 198.SUPPL 1 (2022) | 37% 3D Conformal radiotherapy used, (max 30% allowed) |
| Schleifenbaum | "Optimising (re-)irradiation for locally recurrent head and neck cancer: impact of dose-escalation, salvage surgery, PEG tube and biomarkers on oncological outcomes—a single centre analysis" Radiation Oncology (2025) | 43% 3D-Conformal Radiotherapy used, (max 30% allowed) |
| Schoenfeld | "A Phase 2 Clinical Trial of Pembrolizumab with Radiation Following Progression on Anti-Pd-1 Therapy in Patients with Recurrent or Metastatic Squamous Cell Carcinoma of the Head and Neck." International Journal of Radiation Oncology Biology Physics 118.5 (2024) | 88% re-irradiation in overlapping fields (at least 90% required) |
| Scolari | "Re-irradiation for head and neck cancer: outcome and toxicity analysis using a prospective single institution database" Front Oncol (2023) | included |
| Seidl | "Prognostic Factors in Patients Irradiated for Recurrent Head-and-Neck Cancer." Anticancer Res 36.12 (2016) | Only 20% re-irradiated. (at least 90% required) |
| Seiwert | "A Phase I Dose Escalation Study of Ad Gv.Egr.Tnf.11d (Tnferadetmm Biologic) with Concurrent Chemoradiotherapy in Patients with Recurrent Head and Neck Cancer Undergoing Reirradiation." Annals of Oncology 24.3 (2013) | only including unresected cancer, no comparison to IMRT possible, unclear how many patients received IMRT for re-irradiation |
| Sharma | "Tolerability of Salvage Daily Intensity Modulated Radiation Therapy in Recurrent Head and Neck Cancers Previously Irradiated." American Journal of Clinical Oncology-Cancer Clinical Trials 32.1 (2009) | unclear how many received adjuvant-irradiation, only 14 patients included (at least 10 patients must be in each arm), Results from patient with adjuvant and definitive radiotherapy were not reported separately, hence no comparison between aIMRT and dIMRT could be made |
| Shaukat | "Clinical Outcomes of Definitive Radiotherapy Delivered by Helical Tomotherapy" Journal of the College of Physicians and Surgeons Pakistan (2025) | only definitive VMAT, 64% Head and neck cancer, no comparison to aIMRT possible |
| Sher | "Efficacy and Toxicity of Reirradiation Using Intensity-Modulated Radiotherapy for Recurrent or Second Primary Head and Neck Cancer." Cancer 116.20 (2010) | compares median survival between aIMRT and dIMRT, no 1-year or 2-year OS stated |
| Sher | "Re-Irradiation with Imrt for Head and Neck Cancer." International Journal of Radiation Oncology Biology Physics 66.3 (2006) | unclear how many received adjuvant radiotherapy |
| Shikama | "Validation of Nomogram-Based Prediction of Survival Probability after Salvage Re-Irradiation of Head and Neck Cancer." Jpn J Clin Oncol 43.2 (2013) | 71% received conventional re-irradiation (maximum 30% allowed), 50% nasopharyngeal carcinoma (maximum 20% allowed) |
| Shuja | "Clinical Outcomes after Re-Irradiation in Recurrent Head and Neck Cancers Treated with Intensity Modulated Proton and Photon Therapies." International Journal of Radiation Oncology Biology Physics 105.1 (2019) | unclear cancer histology, only 18 IMRT patients included (at least 10 patients must be in each arm), As no separate outcome values for aIMRT and dIMRT-cohort were stated/ provided, no comparison between aIMRT and IMRT was possible |
| Singh | "Re-Irradiation of Recurrent and/or Persistent Squamous Cell Carcinoma of Head and Neck Region." Radiotherapy and Oncology 99 (2011) | no info about re-irradiation technique, no info on tumor localisation, results not readable in pdf, Results from patient with adjuvant and definitive radiotherapy were not reported separately in the text, hence no comparison between aIMRT and dIMRT could be made |
| Sircar | "Location of Locoregional Failure after Salvage Re-Irradiation of Recurrent Head and Neck Cancer." Radiotherapy and Oncology 96 (2010) | 58% patients received IMRT (at least 70% required), overlap to Ward et al 2018 |
| Sircar | "Predicting Outcomes after Salvage Reirradiation of Recurrent Head and Neck Cancer." International Journal of Radiation Oncology Biology Physics 78.3 (2010) | Results from patient with adjuvant and definitive radiotherapy were not reported separately, hence no comparison between aIMRT and dIMRT could be made, but surgery was significant factor for OS (overlap to Ward et al 2018) |
| Skjotskift | "Early Toxicity Profile after Re-Irradiation with Dose Painting of Head and Neck Cancer." Radiotherapy and Oncology 127 (2018) | only states toxicities of dose painting, only includes unresected patients (authors correspondence) , no comparison to aIMRT possible |
| Skjøtskift | "Dose Painting for Re-Irradiation of Head and Neck Cancer." Acta Oncol 57.12 (2018) | only dosimetric, no endpoint of interest stated no comparison to aIMRT possible |
| Slater | "Reirradiation in Head and Neck Squamous Cell Carcinoma; Prognostic Indicators, Oncologic and Functional Outcomes." Am J Otolaryngol 45.6 (2024) | no info, how many patients received IMRT, Results from patient with adjuvant and definitive radiotherapy were not reported separately, hence no comparison between aIMRT and dIMRT could be made for the outcomes of interest. LRC was lower in surgery group although this was not significant |
| Solanki | "Pathologic Predictors for Outcome in Recurrent and Second Primary Head and Neck Cancer Patients Undergoing Surgery Followed by Concurrent Chemo-Reirradiation." International Journal of Radiation Oncology Biology Physics 72.1 (2008) | 100% adjuvant radiotherapy, unclear if IMRT used, no comparison to dIMRT possible |
| Soliman | "Utility of Chemotherapy in Intensity Modulated and Proton Beam Reirradiation Therapy of Recurrent Head and Neck Squamous Cell Malignancies." American Journal of Clinical Oncology-Cancer Clinical Trials 44.10 (2021) | Results from patient with adjuvant and definitive radiotherapy were not reported separately, hence no comparison between aIMRT and dIMRT could be made |
| Spencer | "Final Report ofradiotherapyog 9610, a Multi-Institutional Trial of Reirradiation and Chemotherapy for Unresectable Recurrent Squamous Cell Carcinoma of the Head and Neck." Head Neck 30.3 (2008) | no IMRT used (at least 70% required) |
| Stewart | "Long-Term Survival and Local Control after Reirradiation for Locally Recurrent Carcinoma of the Head and Neck." International Journal of Radiation Oncology Biology Physics 75.3 (2009) | no info about adjuvant radiotherapy and method of re-irradiation, Results from patient with adjuvant and definitive radiotherapy were not reported separately, hence no comparison between aIMRT and dIMRT could be made |
| Studer | "Outcome in recurrent head neck cancer treated with salvage-IMRT" Radiat Oncol 3 (2008) | no initial radiotherapy, no overlapping fields |
| Stuschke | "Re-Irradiation of Recurrent Head and Neck Carcinomas: Comparison of Robust Intensity Modulated Proton Therapy Treatment Plans with Helical Tomotherapy." Radiat Oncol 8 (2013) | planning study, 7 patients (at least 10 required in each arm) |
| Suh | "Reirradiation after Salvage Surgery and Microvascular Free Flap Reconstruction for Recurrent Head and Neck Carcinoma." Otolaryngol Head Neck Surg 139.6 (2008) | 100% postoperative-irradiation, no comparison to dIMRT possible |
| Sulman | "IMRT Re-Irradiation for Locoregionally Recurrent or in-Field Second Primary Head and Neck Cancer." International Journal of Radiation Oncology Biology Physics 66.3 (2006) | In this abstract, Results from patient with adjuvant and definitive radiotherapy were not reported separately, hence no comparison between aIMRT and dIMRT could be made The later published full text article was included as it stated the desired outcomes, separately. |
| Sulman | "IMRT reirradiation of head and neck cancer-disease control and morbidity outcomes" Int J Radiat Oncol Biol Phys (2009) | included |
| Tahara | "Cisplatin/5-Fu 100/1000 in Japanese Patients with Recurrent/ Metastatic Scchn Management of Recurrent Head-and-Neck Squamous Cell Carcinoma." International journal of radiation oncology biology physics 88.2 (2014) | no radiotherapy applied |
| Takiar | "Reirradiation of Head and Neck Cancers with Intensity Modulated Radiation Therapy: Outcomes and Analyses." Int J Radiat Oncol Biol Phys 95.4 (2016) | only compares median OS, and 5-year OS, 5-year LRC and long-term toxicity between aIMRT and dIMRT |
| Takiar | "Reirradiation of Head and Neck Cancers with Imrt: Updated Outcomes and Analysis." Oral Oncology 49 (2013) | Results from patient with adjuvant and definitive radiotherapy were not reported separately, hence no comparison between aIMRT and dIMRT could be made |
| Takiar | "Reirradiation of Head-and-Neck Cancers: An Md Anderson Update." International Journal of Radiation Oncology Biology Physics 87.2 (2013) | Results from patient with adjuvant and definitive radiotherapy were not reported separately, hence no comparison between aIMRT and dIMRT could be made |
| Talapatra | "Hypofractionated Image Guided Re-Irradiation in Head - Neck Cancers; an Indian Experience." Radiotherapy and Oncology 152 (2020) | no info about histology, only definitive IMRT was used, no comparison to aIMRT was possible |
| Tamari | "Treatment Outcome of Definitive Re-Irradiation by Intensity-Modulated Radiation Therapy for 214 Patients; Can Definitive Re-Irradiation Can Prolong Survival of Patients with Recurrence in Previously Irradiated Site?" International Journal of Radiation Oncology Biology Physics 105.1 (2019) | only 23,8% cancers in the head and neck were included |
| Tanvetyanon | "Effect of Co-Morbidity Burden on Survival Following Salvage Re-Irradiation for Patients with Recurrent or Second Primary Squamous Cell Carcinoma of the Head and Neck." Journal of Clinical Oncology 26.15 (2008) | IMRT and 3D-conformal radiotherapy used in only 51% of the patients, (at least 70% IMRT required) |
| Tanvetyanon | "Prognostic Factors for Survival after Salvage Reirradiation of Head and Neck Cancer." J Clin Oncol 27.12 (2009) | no info on radiotherapy technique, no comparison for adjuvant and definitive therapy on endpoints of interest |
| Tao | "Twice Daily Reirradiation with Cetuximab Vs Once Daily Chemort after Surgery in Head and Neck Cancer." Radiotherapy and oncology 127 (2018) | only surgically treated patients with no margins included, no comparison to definitive treatment possible, no info, if IMRT was used |
| Tao | "Randomized Trial Comparing Two Methods of Re-Irradiation after Salvage Surgery in Head and Neck Squamous Cell Carcinoma: Once Daily Split-Course Radiotherapy with Concomitant Chemotherapy or Twice Daily Radiotherapy with Cetuximab." Radiother Oncol 128.3 (2018) | only surgically treated patients with no margins included, no comparison to definitive treatment possible, no info, if IMRT was used |
| Tie | "Enhancing the Management of Locally Advanced Head and Neck Malignancies and Cases with Local/Neck Recurrence and Metastasis through the Integration of Anlotinib with Concurrent Radiochemotherapy." Anti-Cancer Drugs 35.8 (2024) | 28% recurrences only, (90% overlapping fields required) |
| Tortochaux | "Randomized Phase III Trial (GROTEC 98-03) Comparing Re-Irradiation Plus Chemotherapy Versus Methotrexate in Patients with Recurrent or a Second Primary Head and Neck Squamous Cell Carcinoma, Treated with a Palliative Intent." Radiother Oncol 100.1 (2011) | 3D Conformal or conventional radiotherapy only (max 30% allowed) |
| Toya | "Radiation Therapy Oncology Group 8502 “QUAD shot” regimen using volumetric modulated arc therapy for incurable head and neck cancer" | no surgery for recurrence, no comparison to dIMRT possible |
| Travancinha | "Re-Irradiation with Intensity Modulated Radiotherapy for Recurrent or Second Primary Head and Neck Cancer." Radiotherapy and Oncology 111 (2014) | 31% Nasopharyngeal cancer (maximum 20% allowed) |
| Van Waes | "Inhibition of Nuclear Factor-Κb and Target Genes During Combined Therapy with Proteasome Inhibitor Bortezomib and Reirradiation in Patients with Recurrent Head-and-Neck Squamous Cell Carcinoma." International Journal of Radiation Oncology Biology Physics 63.5 (2005) | most patients were not treated with IMRT (at least 70% required) |
| Vargo | "Optimal Treatment Selection of Stereotactic Body Radiation Therapy and Intensity Modulated Radiation Therapy for Reirradiation of Head and Neck Cancer: A Multi-Institution Comparison." International Journal of Radiation Oncology Biology Physics 96.2 (2016) | Only including definitively treated patients. No comparison to aIMRT possible |
| Vargo | "A Multi-Institutional Comparison of Sbrt and Imrt for Definitive Reirradiation of Recurrent or Second Primary Head and Neck Cancer." Int J Radiat Oncol Biol Phys 100.3 (2018) | definitive treatment only, no comparison to aIMRT possible |
| Velez | "Reirradiation for Recurrent and New Primary Head and Neck Cancer: A Single-Institutional Report." International Journal of Radiation Oncology Biology Physics 94.4 (2016) | Results from patient with adjuvant and definitive radiotherapy were not reported separately, hence no comparison between aIMRT and dIMRT could be made |
| Velez | "Re-irradiation for recurrent and second primary cancers of the head and neck" Oral Oncol (2017) | included |
| Velez | "Prognostic Significance of Hpv Status in the Re-Irradiation of Recurrent and Second Primary Cancers of the Head and Neck." Am J Otolaryngol 39.3 (2018) | Results from patient with adjuvant and definitive radiotherapy were not reported separately, hence no comparison between aIMRT and dIMRT could be made |
| Vermorken | "Platinum-Based Chemotherapy Plus Cetuximab in Head and Neck Cancer." New England Journal of Medicine 359.11 (2008) | no IMRT used |
| Vikash Babu | "Locally unresectable ongoing head and neck cancer’s, reirradiation with concurrent chemotherapy" Journal of cardiovascular disease research 14.5 (2023) | no info on re-irradiation technique, only unresected cancers included, no comparison to aIMRT possible |
| von der Grun | "Re-Irradiation with Concurrent Nivolumab in Locally Recurrent Head and Neck Cancer." Radiotherapy and Oncology 161 (2021) | no info on re-irradiation technique, only unresected cancers included, no comparison to aIMRT possible |
| von der Grün | "Second Infield Re-Irradiation with a Resulting Cumulative Equivalent Dose (Eqd2(Max) ) of >180 Gy for Patients with Recurrent Head and Neck Cancer." Head Neck 41.4 (2019) | case study (at least 10 patients required in each arm) |
| Vormittag | "Re-Irradiation Combined with Capecitabine in Locally Recurrent Squamous Cell Carcinoma of the Head and Neck. A Prospective Phase Ii Trial." Strahlenther Onkol 188.3 (2012) | most patients did not receive IMRT |
| Wada | "Feasibility and Effectiveness of Palliative Intensity-Modulated Radiotherapy for Carotid Sinus Syndrome Secondary to Recurrent Head and Neck Cancer." BMJ Case Rep 13.6 (2020) | case report only (at least 10 required in each arm) |
| Wald | "Intraoperative electron beam radiotherapy for locoregionally recurrent head and neck cancer" Radiotherapy and Oncology 123 (2017) | intra-operative radiotherapy and comparison post-op EBRT vs. no EBRT, no comparison dIMRT vs aIMRT |
| Waldron | "Complications and Toxicity of Re-Irradiation Following Total Laryngectomy for Laryngeal Cancer." Journal of Radiation Oncology 8.4 (2019) | all adjuvant radiotherapy, no comparison to dIMRT possible |
| Wang | "Patterns of Failure (Pof) after Salvage Reirradiation (Re-Rt) for Recurrent Head-and-Neck Cancer (Hnc): Implications for Field Design and Consolidation Therapy." International Journal of Radiation Oncology Biology Physics 84.3 (2012) | Results from patient with adjuvant and definitive radiotherapy were not reported separately, hence no comparison between aIMRT and dIMRT could be made |
| Wang | "Temporal and Spatial Patterns of Recurrence in Oral Squamous Cell Carcinoma, a Single-Center Retrospective Cohort Study in China." BMC Oral Health 23.1 (2023) | study on recurrence pattern, Results from patient with adjuvant and definitive radiotherapy were not reported separately, hence no comparison between aIMRT and dIMRT could be made for endpoints of interest |
| Ward | "Re-Irradiation of Recurrent or Second Primary Head and Neck Cancer after Prior Radiation: Initial Findings of an American Radium Society™ (Ars) Appropriate Use Criteria Systematic Review." American Journal of Clinical Oncology-Cancer Clinical Trials 44.10 (2021) | Systematic review, no original patient’s data provided |
| Ward | "A Nomogram to Predict Severe Late Toxicity after Definitive Reirradiation for Squamous Carcinoma of the Head and Neck." International Journal of Radiation Oncology Biology Physics 99.2 (2017) | only definitive re-irradiation was used, no comparison to aIMRT was possible |
| Ward | "A Competing Risk Nomogram to Predict Severe Late Toxicity after Modern Re-Irradiation for Squamous Carcinoma of the Head and Neck." Oral Oncol 90 (2019) | Results from patient with adjuvant and definitive radiotherapy were not reported separately, hence no comparison between aIMRT and dIMRT could be made for 1-year and 2-year OS, overlap to Ward et al. 2018 |
| Ward | "Multi-Institution Analysis of Intensity Modulated Radiation Therapy-Based Reirradiation for Head and Neck Cancer: Prognostic Factors and Recursive Partitioning Analysis for Overall Survival." International Journal of Radiation Oncology Biology Physics 96.2 (2016) | meeting formal inclusion criteria, excluded because of overlap to Ward et al 2018 |
| Ward | "Refining Patient Selection for Reirradiation of Head and Neck Squamous Carcinoma in the IMRT Era: A Multi-institution Cohort Study by the MIRI Collaborative" Int J Radiat Oncol Biol Phys (2018) | included |
| Watkins | "Toxicity and Survival Outcomes of Hyperfractionated Split-Course Reirradiation and Daily Concurrent Chemotherapy in Locoregionally Recurrent, Previously Irradiated Head and Neck Cancers." Head Neck 31.4 (2009) | unclear how many patients received IMRT, Results from patient with adjuvant and definitive radiotherapy were not reported separately, hence no comparison between aIMRT and dIMRT could be made |
| Wei | "An open, multicenter, exploratory study of apatinib mesylate maintenance therapy for recurrent/metastatic head and neck squamous cell carcinoma (ChiCTR1800019375)" Head Neck 46.4 (2024) | 15% re-irradiation only (minimum 90% required) |
| Weinberger | "Double trouble: A cohort study of re-irradiation and laryngectomy - Severity of and risk for pharyngocutaneous fistula" Oral Oncol 134 (2022) | 100% adjuvant radiotherapy, no comparison to dIMRT possible |
| Wojcieszek | "Radical Reirradiation in Recurrent Head and Neck Cancer Patients." Strahlentherapie Und Onkologie 183 (2007) | no info on method used for radiotherapy, Results from patient with adjuvant and definitive radiotherapy were not reported separately, hence no comparison between aIMRT and dIMRT could be made |
| Wong | "Reirradiation and Concurrent Chemotherapy after Salvage Surgery: Pay Now or Pay Later." J Clin Oncol 26.34 (2008) | Editorial comment, no original patient data provided |
| Woods | "Automated Non-Coplanar Vmat for Dose Escalation in Recurrent Head and Neck Cancer Patients." Cancers (Basel) 13.8 (2021) | study on pre-treatment, no patient outcomes reported |
| Woods | "A Prospective Phase Ii Study of Automated Non-Coplanar Vmat for Recurrent Head and Neck Cancer: Initial Report of Feasibility, Safety and Patient-Reported Outcomes." Cancers (Basel) 14.4 (2022) | unclear, how many had salvage surgery, Results from patient with adjuvant and definitive radiotherapy were not reported separately, hence no comparison between aIMRT and dIMRT could be made |
| Wozniak | "Long term effectiveness of intraoperative radiotherapy given as a boost in adjuvant treatment for oral cavity cancers" Scientific reports (2025) | No recurrent cancer treated (at least 90% required) |
| Xu | "Beyond Reirradiation: Efficacy and Safety of Three or More Courses of Radiation for Head and Neck Malignancies." Clin Transl Radiat Oncol 23 (2020) | only 33% in OC, OP, L, HP and neck |
| Yamazaki | "Reirradiation for Recurrent Head and Neck Cancers Using Charged Particle or Photon Radiotherapy." Strahlenther Onkol 193.7 (2017) | IMRT with doses >3Gy per day was excluded |
| Yamazaki | "Re-Irradiation for Isolated Neck Recurrence in Head and Neck Tumor: Impact of Rn Category." Sci Rep 14.1 (2024) | definitive SBRT only |
| Yamazaki | "Reirradiation for local recurrence of oral, pharyngeal, and laryngeal cancers: a multi-institutional study" Sci Rep 13.1 (2023) | SBRT used |
| Yang | "Adding Hyperthermia to Salvage Concurrent Chemoradiotherapy for Previously Irradiated Unresectable Recurrent Head and Neck Cancer: A Phase Ii Clinical Trial." International Journal of Radiation Oncology Biology Physics 108.3 (2020) | Hyperthermia used; thus, treatment regimen does not fit inclusion criteria |
| Yang | "Phase II clinical trial assessing the addition of hyperthermia to salvage concurrent chemoradiotherapy for unresectable recurrent head and neck cancer in previously irradiated patients" Radiation Oncology (2025) | Hyperthermia used; thus, treatment regimen does not fit inclusion criteria |
| Yang | "Prognostic Factors and Risk-Stratification Model of Recurrent or Metastatic Head and Neck Squamous Cell Carcinoma Treated with Cetuximab Containing Regimen." BMC Cancer 24.1 (2024) | unclear how many received re-irradiation in overlapping fields, Results from patient with adjuvant and definitive radiotherapy were not reported separately, hence no comparison between aIMRT and dIMRT could be made |
| Yao | "Significant Association of Young Age and Salvage Surgery with Overall Survival in Patients with Recurrent Head and Neck Cancer Treated with Reirradiation and Concurrent Chemotherapy: Preliminary Results from a Phase 2 Multicenter Trial." International Journal of Radiation Oncology Biology Physics 93.3 (2015) | no info how many received adjuvant IMRT, overlap to Awan et al. 2018, Results from patient with adjuvant and definitive radiotherapy were not reported separately, hence no comparison between aIMRT and dIMRT could be made |
| Yao | "Phase 2 Trial of Continuous Course Re-Irradiation Concurrent with Weekly Cisplatinum and Cetuximab for Recurrent Squamous Cell Carcinoma of the Head and Neck: Preliminary Report." International Journal of Radiation Oncology Biology Physics 90 (2014) | no info how many received adjuvant IMRT, overlap to Awan et al. 2018, Results from patient with adjuvant and definitive radiotherapy were not reported separately, hence no comparison between aIMRT and dIMRT could be made |
| Yarbrough | "De-Escalated Therapy and Early Treatment of Recurrences in Hpv-Associated Head and Neck Cancer: The Potential for Biomarkers to Revolutionize Personalized Therapy." Viruses-Basel 16.4 (2024) | Review, no original patient data presented |
| Yoshimura | "Efficacy and safety of diffusing alpha-emitter radiation therapy (DaRT) for head and neck cancer recurrence after radiotherapy" Oral Radiology (2025) | Alpha emitter therapy in 11 patients |
| Yoshida | "Combined external radiotherapy and single-fraction palliative high-dose-rate interstitial brachytherapy for a patient with a base of tongue cancer who had a previous radiation history" Oral Radiology (2025) | Case report of Brachytherapy and IMRT |
| Zakem | "A Safety and Efficacy Comparison of High Dose Definitive (&Gt; 70 Gy) Versus Postoperative Reirradiation Using Intensity Modulated Radiation Therapy (Imrt) in Recurrent or Second Primary Head and Neck Cancer." International Journal of Radiation Oncology Biology Physics 93.3 (2015) | only statement for 1y local control between aIMRT and dIMRT |
| Zandberg | "A Phase Ii Trial of Reirradiation Combined with Pembrolizumab in Patients with Locoregional Inoperable Recurrence or Second Primary Squamous Cell Carcinoma of the Head and Neck (Hnscc)." Journal of Clinical Oncology 42.16 (2024) | definitive IMRT only, no comparison to post-operative IMRT |
| Zhang | "Association of tumor growth rate with overall survival and recurrence among patients with laryngeal squamous cell carcinoma" Head Neck (2025) | Study about impact of cancer growth on survival, no info about re-irradiation and survival rates among these patients |
| Zhang | "Development and validation of a nomogram to predict overall survival in patients with External auditory canal cancer" Radiotherapy and Oncology (2025) | only initial diagnosed cancer, no re-irradiation applied |
| Zhang | "Identification and management of recurrent oral squamous cell carcinoma in the clinical presentation of osteoradionecrosis: a single-center case series for treatment experience sharing" Bmc Oral Health (2025) | Case series only including 6 patients |
| Zwicker | "Reirradiation with Intensity-Modulated Radiotherapy in Recurrent Head and Neck Cancer." Head Neck 33.12 (2011) | Results from patient with adjuvant and definitive radiotherapy were not reported separately, hence no comparison between aIMRT and dIMRT could be made |
| Zwicker | "Combined Radio Immunotherapy with Cetuximab for Re-Irradiation of Cancer Recurrence in the Skull/Neck Area." Strahlentherapie Und Onkologie 185 (2009) | 40% adjuvant IMRT, only 10 patients included (at least 10 patients required in each arm) |
| Zwicker | "Imrt Reirradiation with Concurrent Cetuximab Immunotherapy in Recurrent Head and Neck Cancer." Strahlenther Onkol 187.1 (2011) | 40% adjuvant IMRT, only 10 patients included (at least 10 patients required in each arm) |
|  |  |  |

*Supplementary Table A.1: Summarized assessment for the retrieved studies.
dIMRT = definitive IMRT, aIMRT = adjuvant IMRT, OC = Oral cavity, OP = Oropharynx, L = Larynx, HP = Hypopharynx , SBRT = Stereotactic body radiotherapy*
