# Supplementary material for: Challenges in recurrent head and neck squamous cell cancer treatment: systematic review and meta-analysis comparing efficacy and toxicity between post-operative and definitive IMRT-based reirradiation
Source: Clin Transl Radiat Oncol. 2025 Oct 25;56:101061. doi: 10.1016/j.ctro.2025.101061 (PMC12630038; doi:10.1016/j.ctro.2025.101061)
Supplement: Supplementary Data 11 [file mmc11.docx]

| Population | - recurrent or second primary HNSCC (at least 70% of the study population must have histologically proven squamous cell as primary cancer) in a previously irradiated field - older than 18 years - not diagnosed with nasopharyngeal cancer (trials with more than 20% nasopharyngeal cancer will be excluded) - at least 50% of the irradiated cancers should be located in the following regions: oral cavity, oropharynx, hypopharynx, larynx and neck - at least 10 patients included in each arm |
| --- | --- |
| Intervention | Adjuvant IMRT   - patients must have undergone surgery for their recurrence (at least 90% post-operative reirradiation) - at least fifteen planned fractions of at least 1 Gy / maximum 3Gy each and a median cumulative dose >50Gy - at least 70% reirradiated with IMRT - Delivered in a previously irradiated field (>90% of the study population must have received radiotherapy in overlapping fields) - with or without concurrent chemotherapy - no additional brachytherapy or SBRT |
| Control intervention | Definitive IMRT   - at least fifteen planned fractions of at least 1 Gy each and a median cumulative dose >50Gy - at least 70% reirradiated with IMRT - delivered in a previously irradiated field (>90% of the study population must have received radiotherapy in overlapping fields) - with or without concurrent chemotherapy - no preceding surgery other than biopsy for recurrence - no additional brachytherapy or SBRT |
| Outcome | Critical (primary) outcome:   - 1-and- 2-year overall survival   Secondary outcomes:   - 1-and 2-year locoregional control - 1-and 2-year progression-free survival, - radiotherapy-related toxicities - treatment related death |

Supplementary Table A.2: PICO criteria
HNSCC= Squamous cell carcinoma of the Head and Neck, SBRT = Stereotactic Body Radiotherapy, IMRT = Intensity modulated radiotherapy.
