# Supplementary material for: Challenges in recurrent head and neck squamous cell cancer treatment: systematic review and meta-analysis comparing efficacy and toxicity between post-operative and definitive IMRT-based reirradiation
Source: Clin Transl Radiat Oncol. 2025 Oct 25;56:101061. doi: 10.1016/j.ctro.2025.101061 (PMC12630038; doi:10.1016/j.ctro.2025.101061)
Supplement: Supplementary Data 12 [file mmc12.docx]

|  | Patient characteristics | | | Previous therapy | | postOP Risk  factors (Pat no) | Primary sites | | | | Recurrence sites | | | | |  | | |  |
| --- | --- | --- | --- | --- | --- | --- | --- | --- | --- | --- | --- | --- | --- | --- | --- | --- | --- | --- | --- |
| Author, year | Gender (Pat no) | Additional risk factors (Pat no) | HPV p16 (Pat no) | % previous systemic therapy (Pat no) | % prvious surgery (Pat no) |  | OP | OC | L/HP | NP | OP | OC | Neck | L/HP | NP | GTV (cm^3^) | PTV (cm^3^) |  |  |
| Awan, 2018 | 76% male (34) | NR | NR | 100% (Cetuximab OR Cisplatin) | NR | R1: 100% | 51% (23) | 18% (8) | 27% (12) | 0% | 38%(17) | 13% (6) | 29% (13) | 15.6% (7) | 0% | NR | NR |  |  |
| Biagioli,  2007 | 90% male (37) | NR | NR | NR | NR | R1/2: 100% | 29%(12) | 12% (5) | 32%(13) | 2%(1) | 29%(12) | 12% (5) | 32%(13) | 2%(1) | 0% | NR | NR |  |  |
| Chen, 2022 | 95% male (79) | betel nut chewing: 83% Smoking, 80% Drinking: 70%  (57% all three) | NR | NR | NR | NR | 0% | 100% | 0% | 0% | 0% | 70% (58) | 30% (25) | 0% | 0% | 200 (18-906) | NR |  |  |
| Curtis, 2016 | aIMRT 81% male (34) dIMRT 69% male (27) | Alcohol: aIMRT 33% (14) dIMRT: 26% (10) Smoking aIMRT 74% (30) dIMRT: 66% (26) | NR | NR | NR | R1:  26%,(11),  Pn1:  24% (10),  ECS:  45% (19),  LVI:  24% (10) | aIMRT: 24% (10), dIMRT: 36% (14) | aIMRT: 17% (7), dIMRT 8% (3) | aIMRT: 36% (15), dIMRT 44% (17) | 0% | aIMRT 9% (2-4), dIMRT 35% (14) | aIMRT 14% (6) dIMRT 23% (9) | aIMRT 26% (11) dIMRT 30% (12) | aIMRT  36% (15) dIMRT 15%(6) | 0% | NR |  |  |  |
| Rühle, 2020 | 81% male (39) | Smoking 60% (29) | NR | NR | NR | R1: 47% (8),  Close 12% (2),  ECS 6% (1) | NR | NR | NR | NR | 8.3% (3) | 4,2% (2) | 25% (12) | 41.5% (18) | 17% (8) | NR | 105.1 (16.7-905.3) |  |  |
| Saba, 2024 | 82% male (43) | NR | pos: 12% (6) neg: 18% (9) | NR | NR | NR | 27% (14) | 31% (16) | 25% (13) | 12% (6) | NR | NR | NR | NR | NR | NR | NR |  |  |
| Scolari, 2023 | 92% male (56) | Smoking 74% (45), Current 39% (24) | NR | 30% (18) | 80% (49) | R1: 46% (14),  R2: 37% (11), ECS: 61% (8) | NR | NR | NR | NR | 29.5% (18) | 26 (16) | 16% (10) | 18% (11) | 5% (3) | NR | NR |  |  |
| Sulman, 2009 | 70% male (52) | NR | NR | 36% (26) | NR | R1 20% (4) | NR | NR | NR | NR | 41% (30) | 7% (5) | 0% | 3% (2) | 16% (12) | 64.1 (2.9–425.4) | NR |  |  |
| Velez, 2017 | 59% male (45) | NR | NR | NR | NR | NR | 18% (14) | 26% (20) | 12% (9) | 13% (10) | NR | NR | 40.8% (31) | NR | NR | 56 (0.7–220) | NR |  |  |
| Ward, 2018 | 73% male (301) | current smoker 23% (94) | NR | 44% (182) | 45% (186) | NR | NR | NR | NR | NR | 27% (112) | 16% (66) | 21 (88) | 17 (68) | 10% (42)^a^ | 29 (2.4–515)^b^ | NR |  |  |

*Supplementary Table A.3: Additional study characteristics
NR= Not reported, dIMRT= definitive IMRT-based therapy, aIMRT= adjuvant (post-operative) IMRT-based therapy, Pat.no = Patient number, postOP risk factors: post-operative (pathological)risk factors, OP= Oropharynx carcinoma, OC= Oral cavity carcinoma, L/HP= Laryngeal or Hypopharynx Carcinoma, Neck= isolated neck recurrence, GTV = Gross tumor volume, PTV = planned target volume, R1= R1 resection margin, R2 = R2 resection margin, ECS= Extra-capsular spread, Pn1 = Perneural invasion, LVI: lymphovascular invasion
a. including skull-base
b. known for 147 patients, only*
