# Supplementary material for: Challenges in recurrent head and neck squamous cell cancer treatment: systematic review and meta-analysis comparing efficacy and toxicity between post-operative and definitive IMRT-based reirradiation
Source: Clin Transl Radiat Oncol. 2025 Oct 25;56:101061. doi: 10.1016/j.ctro.2025.101061 (PMC12630038; doi:10.1016/j.ctro.2025.101061)
Supplement: Supplementary Data 13 [file mmc13.docx]

| Author, Year | Performance status % (Pat no.) | stage of recurrence  % (Pat no.) |
| --- | --- | --- |
| Awan et al., 2018 | NR | NR |
| Biagioli et al., 2007 | NR | NR |
| Chen et al., 2022 | ECOG: 0-1: 73% 2-3: 37% | AJCC (7th edition)  I: 2% (2) II: 11% (9)  III: 10% (8) IVA: 41% (34) IVB: 36% (30) |
| Curtis et al., 2016 | NR | NR |
| Rühle et al., 2020 | ECOG:  0: 25% (12), 1: 66,7% (32) 2: 8% (4) | TMN  rT0: 49% (23)  rT1: 8% (4) rT2: 4% (2) rT3: 10% (5) rT4: 29% (14) rN0: 56% (27) rN1: 21% (10) rN2: 19% (9) rN3: 4% (2) M1: 29% (14) |
| Saba et al., 2024 | ECOG: 0: 25% (13) 1: 71% (36) 2: 4% (2) | NR |
| Scolari et al., 2023 | Carlson Comorbidity index:  1: 29.5% (18) 2: 29.5% (18) 3 or more: 41% (25) | TMN  rT0: 16% (10)  rT1: 5% (3) rT2: 16% (10) rT3: 12% (7) rT4: 51% (31) rN0: 54% (33) rN1: 6% (4) rN2: 36% (22) rN3: 3% (2) M1: 0% (0) |
| Sulman et al., 2009 | NR | NR |
| Velez et al., 2017 | Karnofsky-Index:  <80 21.1% (16) > 80 77.6% (59) | NR |
| Ward et al., 2018 | Carlson Comorbidity index:   0: 59.2% (244)  1: 19.7% (81)  2 or more: 20.9% (86) | NR |

*Supplementary Table A.4: Performance status of the included studies
NR = not reported*
