# Supplementary material for: Challenges in recurrent head and neck squamous cell cancer treatment: systematic review and meta-analysis comparing efficacy and toxicity between post-operative and definitive IMRT-based reirradiation
Source: Clin Transl Radiat Oncol. 2025 Oct 25;56:101061. doi: 10.1016/j.ctro.2025.101061 (PMC12630038; doi:10.1016/j.ctro.2025.101061)
Supplement: Supplementary Data 14 [file mmc14.docx]

| Author, year | Judgement | Rationale |
| --- | --- | --- |
| Awan, 2018 | no notable concern about conflict of interest | none stated or otherwise identified |
| Biagioli, 2007 | no notable concern about conflict of interest | none stated or otherwise identified |
| Chen, 2022 | no notable concern about conflict of interest | none stated or otherwise identified |
| Curtis, 2016 | no notable concern about conflict of interest | none stated or otherwise identified |
| Rühle, 2020 | no notable concern about conflict of interest | none stated or otherwise identified |
| Saba, 2024 | **Notable concern about Conflict of Interest** | Bristol-Meyers-Squibb funded study, was involved in trail design, review and approval for publication. The lead author (Saba) reported grants from BMS during his work on the study, as well. However, BMS was not involved in data collection and analysis. We have not identifed considerable missing data. |
| Scolari, 2023 | no notable concern about conflict of interest | none stated or otherwise identified |
| Sulman, 2009 | no statement on funding or competing interest was identified | none stated |
| Velez, 2017 | no notable concern about conflict of interest | none stated or otherwise identified |
| Ward, 2018 | no notable concern about conflict of interest | none stated or otherwise identified |

Supplementary Table A.5 judgment about conflict of interest.
