# Supplementary material for: Challenges in recurrent head and neck squamous cell cancer treatment: systematic review and meta-analysis comparing efficacy and toxicity between post-operative and definitive IMRT-based reirradiation
Source: Clin Transl Radiat Oncol. 2025 Oct 25;56:101061. doi: 10.1016/j.ctro.2025.101061 (PMC12630038; doi:10.1016/j.ctro.2025.101061)
Supplement: Supplementary Data 15 [file mmc15.docx]

| Author, year | 1-year PFS | | 2-year PFS | | 1-year LRC | | 2-year LRC | |
| --- | --- | --- | --- | --- | --- | --- | --- | --- |
|  | dIMRT | aIMRT | dIMRT | aIMRT | dIMRT | aIMRT | dIMRT | aIMRT |
| Awan, 2018 | NR | NR | NR | NR | NR | NR | NR | NR |
| Biagioli, 2007 | NR | NR | NR | NR | NR | NR | NR | NR |
| Chen, 2022 | NR | NR | NR | NR | **34%^a^** | **45^a^** | **11%^a^** | **34%^a^** |
| Curtis, 2016 | NR | NR | NR | NR | **62%^a^** | **74%^a^** | **51%^a^** | **68%^a^** |
| Rühle, 2020 | **31%** | **51%** | **24%** | **38%** | NR | NR | NR | NR |
| Saba, 2024 | **75%** | **57%** | **64%** | **36%** | NR | NR | NR | NR |
| Scolari, 2023 | **22%** | **29%** | **22%** | **13%** | **34%** | **46%** | **34%** | **24%** |
| Sulman, 2009 | **75%** | **76%** | **71%** | **55%** | **84%** | **78%** | **76%** | **64%** |
| Velez, 2017 | NR | NR | NR | NR | NR | NR | NR | NR |
| Ward, 2018 | NR | NR | NR | NR | **60%^b^** | **69%^b^** | **54%^b^** | **60%^b^** |

*Supplementary Table A.6: Secondary endpoints. PFS= Progression-free-survival, LRC = Locoregional control, NR = not reported
dIMRT= definitive IMRT-based therapy, aIMRT= adjuvant (post-operative) IMRT-based therapy
a stated locoregional progression free survival.
b reported locoregional failures. We calculated 100%- locoregional-failure for LRC.*
