# Supplementary material for: Challenges in recurrent head and neck squamous cell cancer treatment: systematic review and meta-analysis comparing efficacy and toxicity between post-operative and definitive IMRT-based reirradiation
Source: Clin Transl Radiat Oncol. 2025 Oct 25;56:101061. doi: 10.1016/j.ctro.2025.101061 (PMC12630038; doi:10.1016/j.ctro.2025.101061)
Supplement: Supplementary Data 16 [file mmc16.docx]

| Data provided | | | | | | |
| --- | --- | --- | --- | --- | --- | --- |
| Study | **1-year  overal survival** | **2-year overal survival** | **1-year locoregional-control** | **2-year locoregional-control** | **1-year progression free survival** | **2-year progeression free survival** |
| Awan | Directly stated | Directly stated | Not reported | Not reported | Not reported | Not reported |
| Biagioli | Graphical | Graphical | Not reported | Not reported | Not reported | Not reported |
| Chen | Graphical | Graphical | Graphical | Graphical | Not reported | Not reported |
| Curtis | Graphical | Directly stated | Graphical | Graphical | Not reported | Not reported |
| Rühle | Via correspondence | Via correspondence | Not reported | Not reported | Via correspondence | Via correspondence l |
| Saba | Directly stated in Supplements | Directly stated in Supplements | Not reported | Not reported | Directly stated in Supplements | Directly stated in Supplements |
| Scolari | Directly stated | Directly stated | Directly stated in Supplements | Directly stated in Supplements | Directly stated in Supplements | Directly stated in Supplements |
| Sulman | Graphical | Graphical | Graphical | Graphical | Not reported | Not reported |
| Velez | Graphical | Graphical | Not reported | Not reported | Not reported | Not reported |
| Ward | Graphical | Directly stated | Graphical | Directly stated | Not reported | Not reported |

Supplementary Table 4: Source for used values
