# Supplementary material for: Challenges in recurrent head and neck squamous cell cancer treatment: systematic review and meta-analysis comparing efficacy and toxicity between post-operative and definitive IMRT-based reirradiation
Source: Clin Transl Radiat Oncol. 2025 Oct 25;56:101061. doi: 10.1016/j.ctro.2025.101061 (PMC12630038; doi:10.1016/j.ctro.2025.101061)
Supplement: Supplementary Data 17 [file mmc17.docx]

| Authors | 1) | 2) | 3) | 4) | 1a) | 1b) | | 1) | 2) | 3) | NOS stars | AHRQ standards | Explanation |
| --- | --- | --- | --- | --- | --- | --- | --- | --- | --- | --- | --- | --- | --- |
| Awan et al. (2018) | * | ***** | * | * | **X** | ***** | | * | * | * | 8 | **Good** | Did not control for time to recurrence. Excluded distant metastases at baseline. |
| Biagioli et al. (2007) | * | ***** | * | ***** | **X** | ***** | | * | * | * | 8 | **Good** | Did not control for time to recurrence. Excluded distant metastases at baseline. |
| Chen et al. (2022) | * | ***** | * | ***** | **X** | ***** | | * | * | * | 8 | **Good** | Did not control for time to recurrence. Excluded distant metastases at baseline. |
| Curtis et al. (2016) | * | ***** | * | ***** | **X** | * | | * | * | * | 8 | **Good** | performed univariate Cox Proportional Hazard analysis for disease free interval <24 months and above, this was insignificant for the entire cohort (dichotomous regression was deemed insufficient). Excluded distant metastases at baseline. |
| Rühle et al. (2020) | * | ***** | * | * | **X** | ***** | | * | * | * | 8 | **Good** | Did not control for time to recurrence. Included distant metastases at baseline but we only analysed patients without distant metastasis, so this was also controlled for. |
| Saba et al. (2024) | * | ***** | * | ***** | **X** | * | | * | * | * | 8 | **Good** | Did not control for time to recurrence. Excluded distant metastases at baseline. |
| Scolari et al. (2023) | * | ***** | * | * | **X** | * | | * | * | * | 8 | **Good** | performed multivariate log-regression for disease free interval <24 months and above, this was insignificant for the entire cohort (dichotomous regression was deemed insufficient). Excluded distant metastasis. |
| Sulman et al. (2009) | * | ***** | * | ***** | **X** | * | | * | * | * | 8 | **Good** | performed univariate log-rank test and multivariate Cox Proportional Hazard analysis for time to re-treatment < 46.7 months and above, this was insignificant for the whole cohort (dichotomous regression was deemed insufficient). Excluded distant metastases. |
| Velez et al. (2017) | * | ***** | * | ***** | ***** | ***** | | * | * | * | 9 | **Good** | performed linear univariate and multivariate Cox Proportional Hazard analysis for time to recurrence; this was found to be significant. Excluded distant metastases |
| Ward  et al. (2018) | * | * | * | * | ***** | ***** | | * | * | * | 9 | **Good** | performed a linear and non-linear univariate and multivariate Cox Proportional Hazard analysis for time to recurrence; this was found to be significant. Excluded distant metastases. |
|  | Selection | | | | Comparability | |  | Outcome | |  |  |  |  |

Supplementary Table A.8: Results of Risk of Bias assessment for overall survival
NOS stars= Newcastle Ottawa scale stars/rating
Comparability: 1a): Controlled for time to recurrence. When using regression for control, only continuous analysis (linear or non-linear) was deemed sufficient 1b) Controlled for distant metastasis at baseline
