# Supplementary material for: Challenges in recurrent head and neck squamous cell cancer treatment: systematic review and meta-analysis comparing efficacy and toxicity between post-operative and definitive IMRT-based reirradiation
Source: Clin Transl Radiat Oncol. 2025 Oct 25;56:101061. doi: 10.1016/j.ctro.2025.101061 (PMC12630038; doi:10.1016/j.ctro.2025.101061)
Supplement: Supplementary Data 18 [file mmc18.docx]

| **Certainty assessment** | | | | | | | **№ of patients** | | **Effect** | | **Certainty** | **Importance** |  |
| --- | --- | --- | --- | --- | --- | --- | --- | --- | --- | --- | --- | --- | --- |
| **№ of studies** | **Study design** | **Risk of bias** | **Inconsistency** | **Indirectness** | **Imprecision** | **Other considerations** | **definitive IMRT** | **adjuvant IMRT** | **Relative (95% CI)** | **Absolute (95% CI)** |  |  |  |
| **1-year overall survival** | | | | | | | | | | | | | |
| 10 | non-randomised studies | serious^a^ | not serious | not serious | Not serious | publication bias strongly suspected^c^ | 281/511  (55.2%) | 314/461 (68.1%) | **RR 0.84** (0.76 to 0.93) | **11 fewer per 100** (from 16 fewer to 5 fewer) | ⨁◯◯◯ Very low^a,b,c^ | Critical Outcome |  |
| **2-year overall survival** | | | | | | | | | | | | | |
| 10 | non-randomised studies | serious^a^ | serious^d^ | not serious | serious^b^ | none | 205/511 (46.9%) | 216/461 (52.3%) | **RR 0.85** (0.65 to 1.12) | **7 fewer per 100** (from 16 fewer to 6 more) | ⨁◯◯◯ Very low^a,b,c,d^ | Critical Outcome |  |
| **1-year progression free survival** | | | | | | | | | | | | | |
| 4 | non-randomised studies | serious^a^ | serious^e^ | not serious | very serious^f^ | none | 68/129 (52.7%) | 53/105 (50.5%) | **RR 1.05** (0.85 to 1.31) | **3 more per 100** (from 8 fewer to 16 more) | ⨁◯◯◯ Very low^a,e,f^ | Additional Outcome |  |
| **2-year progression free survival** | | | | | | | | | | | | | |
| 4 | non-randomised studies | serious^a^ | serious^e^ | not serious | very serious^f^ | publication bias strongly suspected^c^ | 60/129 (46.5%) | 35/105 (33.3%) | **RR 1.29** (0.92 to 1.81) | **10 more per 100** (from 3 fewer to 27 more) | ⨁◯◯◯ Very low^a,f^ | Additional Outcome |  |
| **1-year locoregional control** | | | | | | | | | | | | | |
| 5 | non-randomised studies | serious^a^ | serious^g^ | not serious | serious^b^ | none | 225/386 (58.3%) | 211/325 (64.9%) | **RR 0.89** (0.78 to 0.997) | **7 fewer per 100** (from 13 fewer to 0 fewer) | ⨁◯◯◯ Very low^a,b,^ | Additional Outcome |  |
| **2-year locoregional control** | | | | | | | | | | | | | |
| 5 | non-randomised studies | serious^a^ | serious^g^ | not serious | very serious^f^ | none | 194/386 (50.3%) | 172/325 (52.9%) | **RR 0.93** (0.78 to 1.12) | **4 fewer per 100** (from 12 fewer to 6 more) | ⨁◯◯◯ Very low^a,f,g^ | Additional Outcome |  |
| **Severe acute and late radiotoxicity** | | | | | | | | | | | | | |
| 2 | non-randomised studies | very serious^a^ | very serious^h^ | not serious | extremely serious^i^ | all plausible residual confounding would reduce the demonstrated effect | No conclusive findings can be reported on serious radiotoxicity between definitive and adjuvant IMRT-based re-irradiation | | | | ⨁◯◯◯ Very low^a,h,i^ | Additional Outcome |  |

Supplementary Table A.9 GRADE evidence profile

CI: confidence interval; RR: risk ratio
a. No randomisation was used. Important confounders were not controlled for.
b. Wide 95% Confidence interval that cannot be attributed to heterogeneity among studies alone
c. Visual inspection of funnel plot shows asymmetry
d. Visually inconsistency and statistical analyses also showing heterogeneity.
e. Visual inconsistency. Variation in chemotherapy regimen used. Too few studies identified.
f. Very wide 95% Confidence interval. Only few studies with too few results identified.
g. Visual inconsistency
h. Contrary results in narrative review
i. Imprecise methods and reporting
