# Supplementary material for: Challenges in recurrent head and neck squamous cell cancer treatment: systematic review and meta-analysis comparing efficacy and toxicity between post-operative and definitive IMRT-based reirradiation
Source: Clin Transl Radiat Oncol. 2025 Oct 25;56:101061. doi: 10.1016/j.ctro.2025.101061 (PMC12630038; doi:10.1016/j.ctro.2025.101061)
Supplement: Supplementary Data 19 [file mmc19.docx]

| Authors | 1) | 2) | 3) | 4) | 1a) | 1b) | | 1) | 2) | 3) | NOS stars | AHRQ standards | Explanation |
| --- | --- | --- | --- | --- | --- | --- | --- | --- | --- | --- | --- | --- | --- |
| Chen et al. (2022) | * | ***** | * | ***** | **X** | * | | * | * | * | 8 | **Good** | Did not use any systemic therapy. Excluded distant metastases. |
| Curtis et al. (2026) | * | ***** | * | ***** | ***** | * | | * | * | * | 9 | **Good** | Performed univariate performed Cox proportional hazard model for use of chemotherapy and Cisplatin/Cetuximab, both were found significant. Excluded distant metastases |
| Scolari et al. (2023) | * | ***** | * | * | **X** | * | | * | * | * | 8 | **Good** | Performed log-regression for chemotherapy indicated but not administered, early terminated and fully administered but 4 patients had no indication and were not included in this regression, thus this approach was deemed insufficient |
| Sulman et al. (2009) | * | ***** | * | ***** | ***** | **X** | | * | * | * | 8 | **Good** | Performed univariate log-rank test and multivariate cox proportional hazard model analysis of chemotherapy used, this was not significant. Includes Kaplan-Meyer-Curve for curative and palliative intent but does not adjust for this confounder. |
| Ward et al. (2028) | * | * | * | * | ***** | ***** | | * | * | * | 8 | **Good** | Performed univariate and multivariate Cox proportional hazard analysis for systemic therapy; this was insignificant. Excluded distant metastases |
|  | Selection | | | | Comparability | |  | Outcome | |  |  |  |  |

Supplementary Table A.10: Results of Risk of Bias assessment for locoregional control
NOS stars= Newcastle Ottawa scale stars/rating
Comparability: 1a): Controlled for systemic therapy used 1b) Controlled for curative intent radiotherapy
