# Supplementary material for: Challenges in recurrent head and neck squamous cell cancer treatment: systematic review and meta-analysis comparing efficacy and toxicity between post-operative and definitive IMRT-based reirradiation
Source: Clin Transl Radiat Oncol. 2025 Oct 25;56:101061. doi: 10.1016/j.ctro.2025.101061 (PMC12630038; doi:10.1016/j.ctro.2025.101061)
Supplement: Supplementary Data 20 [file mmc20.docx]

| Authors | Year | 1) | 2) | 3) | 4) | 1a) | 1b) | 1) | 2) | 3) | NOS stars | AHRQ standards | Explanation |
| --- | --- | --- | --- | --- | --- | --- | --- | --- | --- | --- | --- | --- | --- |
| Rühle et al. (2020) | 2020 | * | ***** | * | * | ***** | ***** | * | * | * | 8 | **Good** | Performed univariate Cox Proportional Hazard analysis for chemotherapy use, both was insignificant for the entire cohort. We only analysed subgroup without distant metastasis at baseline, so this was also controlled for |
| Saba et al. (2024) | 2024 | * | ***** | * | ***** | ***** | * | * | * | * | 9 | **Good** | Excluded distant metastases at baseline. Treated all patients with the same immunotherapy |
| Scolari et al. (2023) | 2023 | * | ***** | * | * | * | **X** | * | * | * | 8 | **Good** | Excluded distant metastases at baseline. Performed log-regression for chemotherapy indicated but not administered, early terminated and fully administered but 4 patients had no indication and were not included in this regression, thus this approach was deemed insufficient. |
| Sulman et al. (2009) | 2009 | * | ***** | * | ***** | ***** | ***** | * | * | * | 8 | **Good** | Excluded distant metastases at baseline. Performed univariate log-rank test and multivariate cox proportional hazard model analysis of chemotherapy used, this was not significant. Includes Kaplan-Meyer-Curve for curative and palliative intent but does not adjust for this confounder. |
|  |  | Selection | | | | Comparability |  | Outcome | |  |  |  |  |

Supplementary Table A.11: Results of Risk of Bias assessment for progression free survival
NOS stars= Newcastle Ottawa scale stars/rating
Comparability: 1a): Controlled for distant metastasis 1b) Controlled for systemic therapy
