# Supplementary material for: Challenges in recurrent head and neck squamous cell cancer treatment: systematic review and meta-analysis comparing efficacy and toxicity between post-operative and definitive IMRT-based reirradiation
Source: Clin Transl Radiat Oncol. 2025 Oct 25;56:101061. doi: 10.1016/j.ctro.2025.101061 (PMC12630038; doi:10.1016/j.ctro.2025.101061)
Supplement: Supplementary Data 21 [file mmc21.docx]

| Authors | Year | 1) | 2) | 3) | 4) | 1a) | 1b) | | 1) | 2) | 3) | NOS stars | AHRQ standards | Explanation |
| --- | --- | --- | --- | --- | --- | --- | --- | --- | --- | --- | --- | --- | --- | --- |
| Awan et al. | 2018 | * | ***** | * | * | ***** | **X** | | * | * | * | 8 | **Good** | At least 45Gy pre-irradiated. Did not control for time to recurrence. |
| Biagioli et al. | 2007 | * | ***** | * | ***** | **X** | **X** | | * | * | * | 7 | **Poor** | Did not control for previous radiation dose. Did not control for time to recurrence |
| Chen et al. | 2022 | * | ***** | * | ***** | ***** | **X** | | * | * | * | 8 | **Good** | At least 45Gy pre-irradiated. Did not control for time to recurrence. |
| Curtis et al. | 2016 | * | ***** | * | **X^a^** | **X** | ***** | | * | * | * | 7 | **Good** | Did not control for previous radiation dose. Did not control for time to recurrence. |
| Rühle et al. | 2020 | * | ***** | * | * | **X** | **X** | | * | * | * | 7 | **Poor** | Did not control for previous radiation dose. Did not control for time to recurrence |
| Saba et al. | 2024 | * | ***** | * | ***** | **X** | **X** | | * | * | * | 7 | **Poor** | Did not control for previous radiation dose. Did not control for time to recurrence. |
| Scolari et al. | 2023 | * | ***** | * | * | * | **X** | | * | * | * | 8 | **Good** | At least 56Gy pre-irradiated. Did not control for time to recurrence. |
| Sulman et al. | 2009 | * | ***** | * | ***** | **X** | **X** | | * | * | * | 7 | **Poor** | Did not control for previous radiation dose. Did not control for time to recurrence. |
| Velez et al. | 2017 | * | ***** | * | ***** | ***** | ***** | | * | * | * | 9 | **Good** | performed linear univariate and multivariate Cox Proportional Hazard analysis for time between radiation courses and initial radiation dose. Both were insignificant. |
| Ward et al. | 2018 | * | * | * | * | ***** | **X** | | * | * | * | 8 | **Good** | At least 40Gy pre-irradiated. Did not control for time to recurrence. |
|  |  | Selection | | | | Comparability | |  | Outcome | |  |  |  |  |

Supplementary Table A.12: Results of Risk of Bias assessment for radiotoxicity
NOS stars= Newcastle Ottawa scale stars/rating
Comparability: 1a): Controlled for initial radiation dose 1b) Controlled for time between radiation courses. When using regression for control, only continuous analysis (linear or non-linear) was deemed
a. not clearly stated, if late complications were absent before re-irradiation
