# Supplementary material for: Challenges in recurrent head and neck squamous cell cancer treatment: systematic review and meta-analysis comparing efficacy and toxicity between post-operative and definitive IMRT-based reirradiation
Source: Clin Transl Radiat Oncol. 2025 Oct 25;56:101061. doi: 10.1016/j.ctro.2025.101061 (PMC12630038; doi:10.1016/j.ctro.2025.101061)
Supplement: Supplementary Data 22 [file mmc22.docx]

| Author, year | Overall Radiotoxicity | | | Acute Radiotoxicity | | | | Late Radiotoxicity | | | | Scoring system used |
| --- | --- | --- | --- | --- | --- | --- | --- | --- | --- | --- | --- | --- |
|  | Patient number available^x^ | **≥Grade 3**  (Pat no) | **Grade 5** (Death)^*^ | Patient number available^x^ | **≥Grade 3** | **Grade 3** | **Grade 4** | Patient number available^x^ | **≥Grade 3** | **Grade 3** | **Grade 4** |  |
| Awan, 2018 | 45 | **76% (34)** | **0%** | 45 | **64% (29)** | **62% (28)** | **2% (1)** | 45 | **11% (5)** | **11% (5)** | **0%** | **CTCAE V3.0** |
| Biagioli, 2007 | 41 | **35% (14)** | **0%** | 41 | **20% (8)** | NR | NR | 41 | **15% (6)** | **NR** | **NR** | **RTOG** |
| Chen, 2022 | 83 | NR | **0%** | 83 | NR | NR | NR | 83 | **42% (35)** | **34% (29)** | **7% (6)** | **CTCAE V3.0** |
| Curtis, 2016 | 81 | **NR** | **0%** | 81 | **88% (71)** | **NR** | **NR** | NR | **NR** | **NR** | **NR** | **None stated** |
| Rühle, 2020 | 48 | **29% (14)** | **0%** | 48 | **10% (5)** | **10% (5)** | **0%** | 48 | **19% (9)** | **16% (7)** | **4% (2)** | **CTCAE V4.03** |
| Saba, 2024 | 51 | NR | **0%** | 51 | **12% (6)** | NR | NR | 51 | NR | NR | NR | **CTCAE V4.03** |
| Scolari, 2023 | 54 | **111% (60)** | **0%** | 54 | **100% (54**) | NR | NR | 41 | **15% (6)** | NR | NR | **CTCAE different veriants^a^** |
| Sulman, 2009 | 74 aIMRT:20 dIMRT:54 | **20% (15) aIMRT: 15% (3) dIMRT: 22% (12)** | **1% (1) (aIMRT)** | 74 | NR | NR | NR | NR | NR | NR | NR | **No system used^b^** |
| Velez, 2017 | 76 | NR | **0%** | 76 | NR | NR | NR | 76 aIMRT:31 dIMRT:45 | **33% (25) aIMRT: 29% (9)  dIMRT: 36% (16)** | **18% (14)** | **14% (11)** | **RTOG/EORT** |

Supplementary Table A.13:: Reported Radiotoxicities
Note: This table only included toxicities likely to be caused by radiation like Dysphagia, Dermatitis, Mucositis, Xerostomia, Osteocranionecrosis, Carotid blowout, Fistula, Pain, Dysgeusia, Hoarseness, Nausea, Trismus, Oesophagus stricture, feeding tube, tracheostomy (not attributed to surgery). Patient number available is the stated number of examined patients (If nothing is explicitly stated, the whole cohort was used to calculate the percentages)
NR = not reported, Pat no = Number of patients, CTCAE = Common Terminology Criteria for Adverse Events, RTOG/EORT = RTOG/EORT Morbidity scoring schema

x. Patient number available is the number of participants with complete data on toxicity. If the article didn´t specify, the whole cohort´s number was used to calculate relative toxicity rates
*Treatment related deaths (Grade 5) are included in Overall RTox ≥Grade 3 and acute/ late Rtox ≥Grade 3 , if stated as such
a. no specific information provided, what versions used
b^.^ the authors counted: “Severe reirradiation-related toxicity was defined as toxic events resulting in hospitalization, corrective surgery, or patient death”
c. all counted as acute toxicities
