# Supplementary material for: Challenges in recurrent head and neck squamous cell cancer treatment: systematic review and meta-analysis comparing efficacy and toxicity between post-operative and definitive IMRT-based reirradiation
Source: Clin Transl Radiat Oncol. 2025 Oct 25;56:101061. doi: 10.1016/j.ctro.2025.101061 (PMC12630038; doi:10.1016/j.ctro.2025.101061)
Supplement: Supplementary Data 23 [file mmc23.docx]

| Author, year | Overall Radiotoxicity | | | Acute Radiotoxicity | | | | Late Radiotoxicity | | | | Scoring system used |
| --- | --- | --- | --- | --- | --- | --- | --- | --- | --- | --- | --- | --- |
|  | Patient number available^x^ | **≥Grade 3**  (Pat no) | **Grade 5** (Death)^*^ | Patient number available^x^ | **≥Grade 3** | **Grade 3** | **Grade 4** | Patient number available^x^ | **≥Grade 3** | **Grade 3** | **Grade 4** |  |
| Awan, 2018 | 45 | **276% (106)** | **0** | 45 | **235% (98)** | **213% (88)** | **22% (10)** | 45 | **18% (8)** | **18% (8)** | **0%** | **CTCAE V3.0** |
| Biagioli, 2007 | 41 | **68% (28)** | **0** | 41 | **53% (22)** | NR | NR | 41 | **15% (6)** | **NR** | **NR** | **RTOG** |
| Chen, 2022^t^ | 83 | NR | **0** | 83 | NR | NR | NR | 83 | **42% (35)** | **34% (29)** | **7% (6)** | **CTCAE V3.0** |
| Curtis, 2016 | 81 | **NR** | **0** | 81 | **103% (84)** | **NR** | **NR** | NR | **NR** | **NR** | **NR** | **None stated** |
| Rühle, 2020^t^ | 48 | **29% (14)** | **0** | 48 | **10% (5)** | **10% (5)** | 0 | 48 | **19% (9)** | **16% (7)** | **4% (2)** | **CTCAE V4.03** |
| Saba, 2024 | 51 | **67%(34)** | **0** | 51 | **55% (28)** | NR | NR | 51 | **12% (6)^d^** | NR | NR | **CTCAE V4.03** |
| Scolari, 2023^t^ | 54 | **111% (60)** | **0** | 54 | **100% (54**) | NR | NR | 41 | **15% (6)** | NR | NR | **CTCAE different variants^s^** |
| Sulman, 2009^t^ | 74 aIMRT:20 dIMRT:54 | **20% (15) aIMRT: 15% (3) dIMRT: 22% (12)** | **1% (1) (aIMRT)** | 74 | NR | NR | NR | NR | NR | NR | NR | **No system used^b^** |
| Velez, 2017^t^ | 76 | NR | **0** | 76 | NR | NR | NR | 76 aIMRT:31 dIMRT:45 | **33% (25) aIMRT: 29% (9)  dIMRT: 36% (16** | **18% (14)** | **14% (11)** | **RTOG/EORT** |
| Ward, 2018^t^ | 358 | 38% (134) | **1% (5)^c^** | 358 | **22% (78)** | NR | NR | 358 | **16% (56**) | NR | NR | **CTCAE V4.0** |

Supplementary Table A.14: Reported radiotoxicities of the included studies. Note that some studies reported all occurring toxicities while other only stated those, likely attributed to radiation
NR = not reported, Pat no = Number of patients, CTCAE = Common Terminology Criteria for Adverse Events, RTOG/EORT = RTOG/EORT Morbidity scoring schema
x. Patient number available is the number of participants with complete data on toxicity. If the article didn´t specify, the whole cohort´s number was used to calculate relative toxicity rates
* Treatment related deaths (Grade 5) are included in Overall RTox ≥Grade 3 and acute/ late Rtox ≥Grade 3 , if stated as such
t. this study only reported re-irradiation associated toxicities
a. no specific information provided, what versions used
b. the authors counted: “Severe reirradiation-related toxicity was defined as toxic events resulting in hospitalization, corrective surgery, or patient death”
c. all counted as acute toxicities
d. Only treatment (Nivolumab) related late toxicities
